# The immune gene repertoire of an important viral reservoir, the Australian black flying fox

**DOI:** 10.1186/1471-2164-13-261

**Published:** 2012-06-20

**Authors:** Anthony T Papenfuss, Michelle L Baker, Zhi-Ping Feng, Mary Tachedjian, Gary Crameri, Chris Cowled, Justin Ng, Vijaya Janardhana, Hume E Field, Lin-Fa Wang

**Affiliations:** 1 Bioinformatics Division, The Walter and Eliza Hall Institute of Medical Research, Parkville, Melbourne, VIC, 3052, Australia; 2 Department of Mathematics and Statistics, University of Melbourne, Melbourne, VIC, 3010, Australia; 3 CSIRO Livestock Industries, Australian Animal Health Laboratory, PO Bag 24, Geelong, VIC, 3220, Australia; 4 Center for Evolutionary and Theoretical Immunology, Department of Biology, The University of New Mexico, Albuquerque, NM, 87131, USA; 5 Department of Medical Biology, University of Melbourne, Melbourne, VIC, 3010, Australia; 6 Australian Biosecurity Cooperative Research Centre for Emerging Infectious Diseases, Brisbane, Australia; 7 Queensland Primary Industries and Fisheries, Biosecurity Queensland, Brisbane, Australia

## Abstract

**Background:**

Bats are the natural reservoir host for a range of emerging and re-emerging viruses, including SARS-like coronaviruses, Ebola viruses, henipaviruses and Rabies viruses. However, the mechanisms responsible for the control of viral replication in bats are not understood and there is little information available on any aspect of antiviral immunity in bats. Massively parallel sequencing of the bat transcriptome provides the opportunity for rapid gene discovery. Although the genomes of one megabat and one microbat have now been sequenced to low coverage, no transcriptomic datasets have been reported from any bat species. In this study, we describe the immune transcriptome of the Australian flying fox, *Pteropus alecto*, providing an important resource for identification of genes involved in a range of activities including antiviral immunity.

**Results:**

Towards understanding the adaptations that have allowed bats to coexist with viruses, we have *de novo* assembled transcriptome sequence from immune tissues and stimulated cells from *P. alecto*. We identified about 18,600 genes involved in a broad range of activities with the most highly expressed genes involved in cell growth and maintenance, enzyme activity, cellular components and metabolism and energy pathways. 3.5% of the bat transcribed genes corresponded to immune genes and a total of about 500 immune genes were identified, providing an overview of both innate and adaptive immunity. A small proportion of transcripts found no match with annotated sequences in any of the public databases and may represent bat-specific transcripts.

**Conclusions:**

This study represents the first reported bat transcriptome dataset and provides a survey of expressed bat genes that complement existing bat genomic data. In addition, these data provide insight into genes relevant to the antiviral responses of bats, and form a basis for examining the roles of these molecules in immune response to viral infection.

## Background

Bats make up approximately 20% of the extant mammalian diversity and are the second most species rich mammalian lineage after rodents [1]. The order Chiroptera is divided into two suborders: the Megachiroptera and Microchiroptera. These two lineages are estimated to have diverged approximately 58 million years ago [2]. Megachiroptera consists of a single family, the old world fruit bats, while Microchiroptera includes 17 families of echolocating bats. Bats have a wide geographic distribution and exploit a variety of environmental niches, being absent only from the polar regions. Bats are also hosts to numerous viruses, many of which are highly pathogenic to humans and other mammals yet appear to cause no clinical consequences in bats [3-8]. This group of mammals also shares a variety of unique characteristics that likely facilitate the persistence and spread of the viruses they carry. Highly social species, bats live at much higher densities than other mammals. They are the only mammals capable of powered flight and have long lifespans relative to their body size [9]. Despite their diversity, unique characteristics and role as natural reservoirs for viruses, bats are also the least studied of all mammalian taxa and there is little information available on antiviral immunity in any bat species.

Bats are the natural reservoir hosts of more than 80 viruses, with new viruses or viral sequences of bat origin being discovered each year [9,10]. RNA viruses account for the overwhelming majority of known bat viruses, many of which are among the most deadly known to man, including Ebola, Hendra, Nipah and SARS-like coronaviruses [9]. Many of these viruses, which cause severe morbidity and mortality in humans and other mammals, appear to cause no clinical diseases in bats under natural or experimental infection. The most studied example is the henipaviruses (Hendra and Nipah viruses) which are members of the family Paramyxoviridae*.* Nipah virus has a mortality rate of 40-90% in humans and close to 100% in experimental animal models (cats and hamsters). Yet, infection of *Pteropus vampyrus* (the natural reservoir host of Nipah virus in Malaysia) and *P. poliocephalus* (a related bat species native in Australia) by a high dose of Nipah virus, failed to result in clinical signs of disease [7,8,11]. Other examples of experimental infections of bats including Ebola Zaire, Japanese encephalitis and St. Louis encephalitis viruses have not resulted in any symptoms of disease despite the presence of viral RNA in tissues [3-6]. Experimental infections of *P. poliocephalus* with Nipah virus have demonstrated the presence of serum antibody and viral shedding in the absence of clinical symptoms of disease [11]. The only viruses that have been demonstrated to cause clinical symptoms of disease in bats are rabies virus and the closely related Australian bat lyssavirus [12,13]. However, results of experimental infections are inconsistent with only a small proportion of bats succumbing to infection, and rates of sero-conversion and virus recovery from tissues were reported to be very low [13]. The long co-evolutionary history of bats with viruses has likely resulted in the adaptation of the bats immune system to cope with viral infection. One hypothesis is that the innate immune system rapidly controls viral replication to very low levels that cause no clinical consequences to bats, but still results in viral shedding and subsequent spillover to other species. However, as little information currently exists on any aspect of bat immunology and few bat-specific reagents exist, this hypothesis remains untested.

Recent years have seen a surge in the availability of whole genome sequence data. Bats were among the organisms sequenced as part of the US National Institutes of Health (NIH)-funded Mammalian Genome Project. These genomic resources are an important step forward in identifying the genes that are involved in antiviral immunity in bats and in providing insights into other unique life history characteristics. There are currently two publicly available bat genome sequences: one from the megabat *P. vampyrus* and a second from the microbat *Myotis lucifugus*. Both bat genomes were initially sequenced to low coverage (2.6X for *P. vampyrus* and 1.7X for *M. lucifugus*, though a draft quality assembly of the *M. lucifugus* genome based on 7X coverage sequencing is now available). Additionally, the annotations were predominantly based upon comparative data. Despite these shortcomings, these projects have an important role to play in revealing the mechanisms that have evolved to allow bats to remain asymptomatic to so many viral diseases.

In order to understand bat-virus interactions, we are developing the Australian black flying fox, *P. alecto,* as a model bat species. *P. alecto* belongs to the family Pteropodidae and is closely related to *P. vampyrus*[14]. These two species are reservoirs for a variety of closely related viruses, the most important of which include the henipaviruses, Hendra virus in *P. alecto* and Nipah virus in *P. vampyrus*[10]. A number of important resources have now been developed for *P. alecto*, including cell lines from a variety of tissues [15]. We have also begun to identify some of the genes involved in immune responses in this species and carry out functional studies in bat cells [16-21]. To begin to characterise the immune gene repertoire of *P. alecto*, we sequenced the transcriptome of bat immune tissues and mitogen-stimulated cells using the Illumina platform. To our knowledge, this study represents the first analysis of the transcriptome of any species of bat. Our analysis of the *P. alecto* transcriptome provides information on a variety of immune genes not previously identified in any bat species and represents an important starting point for examining the antiviral activity of these molecules.

## Results and discussion

### Overview of the bat transcriptome

Two separate transcriptomic datasets were generated and raw sequences from each database were submitted to the Sequence Read Archive [SRA: SRR350710.3 and SRR351237.2]. The first was obtained using total RNA extracted from a juvenile male flying fox thymus. Due to its role in central tolerance, the thymus expresses a large proportion of the proteome and therefore allows for the identification of a broad range of genes, including those involved in the immune response. To enrich for sequences corresponding to cytokines and innate immune genes, the second dataset was derived from pooled total RNA obtained from mitogen-stimulated spleen, white blood cells and lymph node and unstimulated thymus and bone marrow obtained from one pregnant female and one adult male flying fox. Cells were stimulated with lipopolysaccharide (LPS) and Ionomycin, which stimulate the production of pro-inflammatory cytokines; PolyIC, a TLR3 ligand; PHA, which triggers T cell activation and PMA, which activates T and B cells.

About 12.5 million 65 bp long reads were obtained from the thymus dataset, while 23.9 million 76 bp long reads were generated from the stimulated pooled sample. Prior to assembly, the raw reads were trimmed of low quality sequence and polyA/T tails, uninformative strings of ‘N’ and primer/adapter contaminants were cleaned. The filtered dataset consisted of 12,399,095 reads from the thymus (between 20-63 bp) and 22,577,294 reads from the stimulated pooled dataset (between 20-73 bp). The filtered reads were *de novo* assembled using the software packages velvet and oases. The resulting oases assemblies consisted of 247,909 contigs (N50 1244 bp) from the thymus and 313,641 contigs (N50 733 bp) from the pooled samples. The largest contigs in the thymus and pooled samples were 11.8 kb and 8.9 kb respectively, both of which correspond to the DNA-dependent protein kinase catalytic subunit (DNA-PKcs) which is represented by a 12.4 kb transcript in other species, including horse. For comparative purposes, an assembly using MIRA was also generated. Summary statistics from the velvet, oases and MIRA assemblies are listed in Additional file 1: Table S1. All subsequent analyses were performed using the oases assemblies.

To identify orthologues of known mammalian protein coding genes, the bat contigs were used to search the KEGG and NCBI non-redundant (NR) protein databases with BLASTX (E-value < 0.001). Of the 247,801 contigs longer than 50 bp in the thymus sequence assembly, about 46% matched annotated proteins in the NR database. For the pooled dataset, about 51% of the 313,528 loci matched proteins in NR. Similar results were obtained for both assembled libraries against the KEGG database.

**Figure 1:**
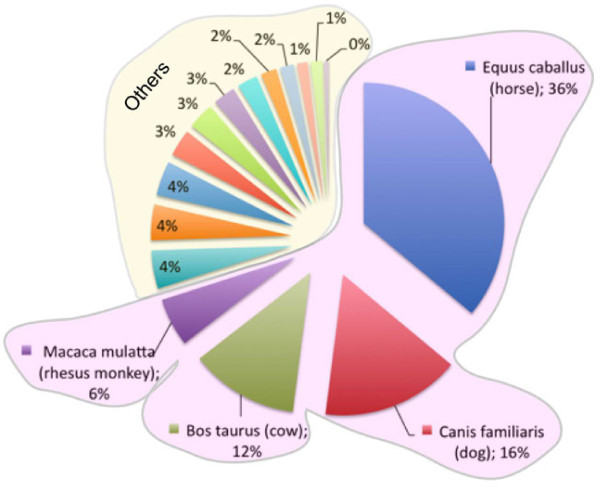
**Bat transcripts are most closely related to horse genes.** Summary of 183,788 hits from the thymus dataset to annotated genes from other species in the KEGG databases. Others from left to right in the pie chart include *Homo sapiens* (human), *Pan troglodytes* (chimpanzee), *Sus scrofa* (pig), *Monodelphis domestica* (opossum), *Rattus norvegicus* (rat), *Mus musculus* (mouse), *Taeniopygia guttata* (zebra finch), *Ornithorhynchus anatinus*, (platypus), *Gallus gallus* (chicken), *Danio rerio* (zebrafish), *Xenopus tropicalis* (western clawed frog), *Xenopus laevis* (African clawed frog).

Of the assembled thymus transcripts annotated using KEGG, 36% of all transcripts were more similar to horse sequences than to any other species, followed by dog (16%) and cow (12%) (Figure 1). Similar results were obtained for the pooled tissue dataset (not shown). This result is consistent with the now generally accepted view that bats belong within Laurasiatheria, which includes Carnivora, Cetartiodactyla (whales and even toed ungulates), Eulipotyphla (moles and shrews), Pholidota (scaly anteater) and Perissodactyla (odd toed ungulates) [22-27]. However, until recently, the phylogenetic relationships within Laurasiatheria have been controversial. Conflicting results have been reported using complete mitochondrial genome sequences to infer phylogenetic relationships with support for a sister relationship between Chiroptera and Fereungulata (Carnivora, Pholidota, Perissodactyla and Cetartiodactyla) or a relationship between Chiroptera and Eulipotyphla [28-30]. Analysis of the nuclear gene, protamine P1, as well as large genomic datasets, has provided evidence that bats are sister to a clade containing Perissodactyla, Carnivora, and Cetartiodactyla [31,32]. The volume of sequence data generated by transcriptome sequencing provides the opportunity for larger scale sequence comparisons than previously possible using the few full length bat genes available or by comparison with the limited whole genome sequence data. Our results support the comparative analysis of retroposon loci which has also demonstrated that bats share a sister relationship with horses, forming a clade with Carnivora [27].

**Figure 2:**
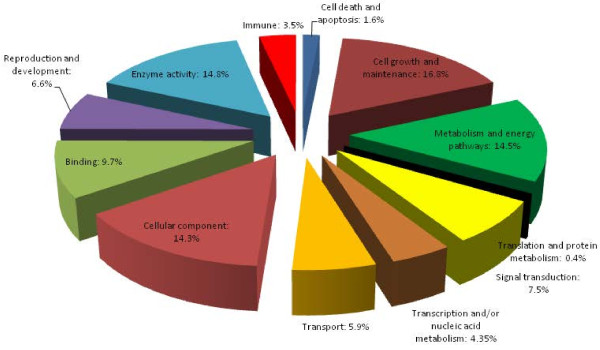
The distribution of 178,554 transcripts that have mapped to human genes from the bat thymus based on GO terms.

Alignment of contigs from the thymus and pooled datasets to the KEGG database identified 178,554 and 285,268 contigs respectively with homology to 16,863 and 16,927 unique human proteins. To explore gene function, Gene Ontology (GO) terms were used. Of contigs that matched proteins in the KEGG database, 86% were assigned GO terms and 78% could be mapped to GO slim terms using GO Term Mapper (Additional file 2: Figure S1). Genes with GO slim terms were further classified into twelve selected classes (Figure 2). The most abundant GO terms found in the thymus dataset were involved in cell growth and maintenance (16.8%), enzyme activity (14.8%), cellular components (14.3%) and metabolism and energy pathways (14.5%). Similar results were obtained for the pooled tissue dataset (data not shown). The GO classification demonstrates that a diverse range of genes were identified in each of our two datasets providing a broad survey of bat genes.

### Immune-related transcripts

**Figure 3:**
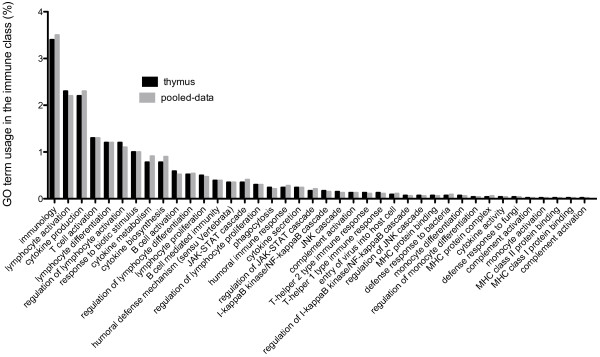
**The distribution of the immune genes from the thymus and pooled tissue datasets annotated at the GO slim level based on the CateGOrizer.** The number of genes in each category is shown as a percent of 904 and 1056 immune genes identified in the thymus and pooled dataset respectively.

A goal of the present study was to identify immune transcripts, particularly those that may play a role in antiviral immunity. Only 3.5% of the bat transcribed genes from each of the datasets showed homology to genes associated with immune function. This represents about 500 different immune-related genes (Figure 2). The bat immune transcripts were further categorised using GO terms to annotate the transcripts into 40 immune categories. Represented in the datasets were genes involved in a broad range of immune activities with lymphocyte activation, cytokine production and T cell activation making up the largest proportions of immune transcripts (Figure 3). Using KEGG codes to identify immune genes, our data revealed 70 genes involved in Toll-like receptor (TLR) cascades, 50 genes involved in B cell activation, 79 involved in T cell activation, 72 involved in natural killer cell cytotoxicity and 41 involved in antigen presentation. Additional immune genes not identified in the KEGG database were obtained by searching sequences from the NR database. The sequences of all genes described in the text are provided in the Additional file 3.

### Innate immunity

One hypothesis for the ability of bats to resist the pathological effects of viral infection is that they are able to rapidly control viral replication early in the immune response through innate antiviral mechanisms. The bat transcriptome contained representatives of a variety of immune genes including pattern recognition receptors, interferons, interferon stimulated genes and natural killer cell receptors.

### Pattern recognition receptors

Pattern recognition receptors (PRR) including TLRs, RIG-I like helicases (RLHs) and nucleotide oligomerisation domain (NOD) like receptors (NLRs) recognise conserved molecular patterns associated with a broad range of pathogens. Both TLRs and RLHs initiate signalling pathways that result in the induction of similar immune and inflammatory responses but are expressed in different locations within the cell and differ in the pathogens they recognise. TLRs are transmembrane proteins expressed by the plasma membrane or endosome and recognise a broad range of pathogens including viruses, bacteria and fungi. Of eleven previously identified *P. alecto* TLR genes [18], only TLR5 was absent from the oases assemblies, however it was present in the MIRA assembly, which used a lower coverage cut-off and is useful for identifying genes with low expression levels. RLHs are expressed in the cytoplasm where they recognise viral RNA and DNA [33,34]. Three bat RLH genes, retinoic-acid-inducible protein I (RIG-I), melanoma-differentiation-associated gene 5 (MDA5) and laboratory of genetics and physiology 2 (LGP2) were identified in our transcriptome datasets and have recently been described in *P. alecto*[17]. These results provide further evidence that bats are able to recognise a broad range of pathogens, similar to other species.

NLRs are a diverse family of cytoplasmic PRRs involved in the activation of a variety of signalling pathways. NLRs are primarily involved in bacterial recognition, although more recently, evidence for recognition of viral RNA and DNA by some members of the NLR family has been reported [35-37]. The only NLRs identified in the bat transcriptome datasets were NOD-like receptor family CARD domain containing 5 (NLRC5) and NLR family, pyrin domain containing 3 (NLRP3). NLRC5 is a recently identified NLR proposed to function as a positive and negative regulator of antiviral immune responses [36]. NLRP3 (also known as NALP3) is activated by a variety of danger signals including viral and bacterial infections and environmental irritants. Activation of NLRP3 in turn activates caspase-1 in the inflammasome which proteolytically cleaves the cytokines IL-1β and IL-18 into active mature peptides [37]. The identification two NLRs with associations with antiviral immunity in the bat transcriptome is remarkable and provides a starting point for understanding the role of NLRs in antiviral immunity in bats.

### Interferons and interferon stimulated transcripts

The interferon (IFN) response is a key component of the innate immune system and the first cytokines induced against viral infection. Since the IFN response is important in the control of viral replication in other mammals, we searched the bat transcriptome for IFNs and IFN stimulated genes (ISGs) that may be critical to the ability of bats to remain asymptomatic to viral infections. Type I (including IFNα and β) and III (IFNλ) IFNs are induced directly in response to viral infection and play a role in the earliest stages of the innate immune response. Type I (α) IFN and its receptor (IFNAR1 and IFNAR2) were identified in the bat transcriptome datasets (Additional file 3). Although type III IFNs, IFNλ1 and IFNλ2 are up-regulated in stimulated bat cells [21], neither of these genes were identified in our datasets, likely reflecting a low expression level in our samples. The IL-10R2 chain of the type III IFN receptor was present in the bat transcriptome, but its partner chain IFNLR1 was not found. Both IL-10R2 and IFNLR1 were recently described in *P. alecto* and IFNLR1 was demonstrated to act as a functional receptor for IFNλ [20].

The induction of type I and type III IFNs results in the transcription of hundreds of ISGs including PRRs that detect viral RNA, transcription factors that result in the amplification of the IFN response and a small number of proteins that are directly responsible for inducing an antiviral state. The ISGs, myxovirus resistance (Mx) GTPases, Protein kinase R (PKR), 2’-5’ oligoadenylate synthetases (OAS), ribonuclease L (RNaseL) and ISG15 are among the proteins with confirmed antiviral activity in other mammals [38]. The bat transcriptome datasets contained genes orthologous to mammalian Mx1, Mx2, OAS1, OAS2, OAS3, OAS-like (OASL), PKR, RNaseL and ISG15 consistent with the presence of an ISG repertoire in bats that is similar to that of other species. These results provide the first evidence that the pathways activated by the IFN response are likely similar in bats to those described in other mammals.

**Figure 4:**
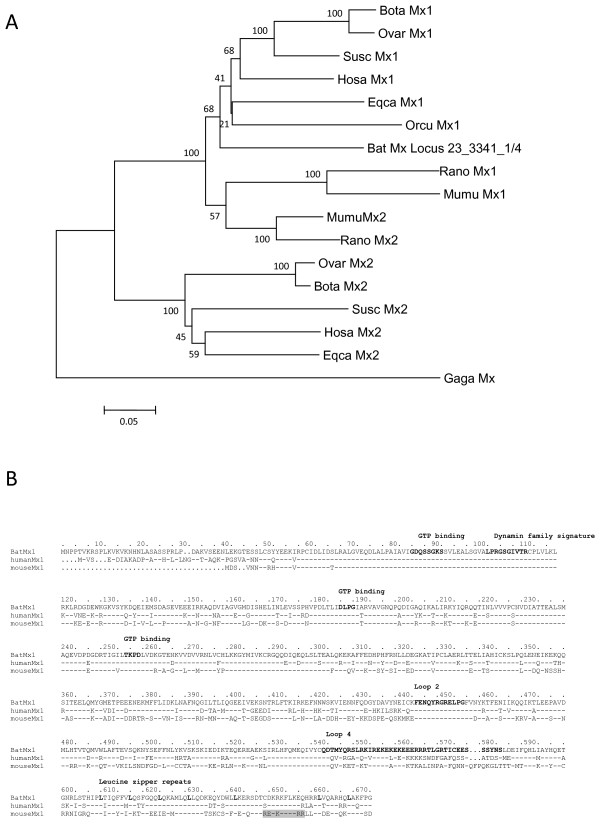
**a. Phylogenetic analysis based on amino acid alignments of bat Mx with representative vertebrate species.** Branch support is indicated as the percentage of 1000 bootstrap replicates and is shown where support is >60%. Species names are: *B. taurus* (Bota), *E. caballus* (Eqca), *G. gallus* (Gaga), *H. sapiens* (Hosa), *M. musculus* (Mumu), *O. aries* (Ovar), *O. cuniculus* (Orcu), *R. norvegicus* (Rano), *S. scrofa* (Susc). Phylogenetic analysis was performed based on alignment of amino acid position 49 to the end of the *P. alecto* Mx sequence with the corresponding region of Mx from other species. **b**. Alignment of deduced amino acid sequences of bat Mx with human and mouse Mx sequences. The conserved tripartite GTP binding domain, dynamin family signature, leucine residues and Loops 2 and 4 are shown in bold. The nuclear localisation signal present in mouse Mx1 is shaded. Dashes indicated similarity and dots indicate gaps.

The Mx gene family is among the best characterised ISGs, first identified as antiviral proteins following the observation that the sensitivity of many inbred mouse strains to orthomyxovirus was solely due to mutations within the Mx locus [39]. The Mx family of GTPases trap essential viral components, and in so doing prevent viral replication at early time points. Although the full spectrum of Mx antiviral activity is unknown, representatives of both RNA and DNA viruses have been shown to be sensitive to the effects of Mx [40]. A full length transcript, encoding a 667 amino acid protein was identified in our bat transcriptome datasets and found to be orthologous to Mx1 based on comparison with known mammalian Mx1 and Mx2 family members (Figure 4a and data not shown). Bat Mx1 contained the highly conserved tripartite GTP-binding domain found in all mammalian Mx proteins. In addition, a dynamin family signature and putative leucine zipper motif were found near the C terminal end, represented by a stretch of evenly spaced leucine residues. The bat protein was also conserved in the region identified as the stalk of human MxA including Loop 2 which is associated with antiviral activity. Consistent with other species, Loop 4 of the MxA stalk is the least conserved region of the bat Mx protein [41]. Loop 4 has been reported to be proteinase K sensitive and may play a role in lipid binding [42,43] (Figure 4b). Bat Mx1 does not contain the stretch of basic amino acids (K/R) near the C terminal end associated with nuclear localisation of mouse Mx1, consistent with the bat protein remaining localised within the cytoplasm [44]. The conservation of key residues important in antiviral activity is consistent with the bat Mx1 playing a role in antiviral immunity similar to other species. The identification of the sequences of important ISGs will now allow us to determine whether functional differences in the initiation and regulation of these proteins account for the differences in susceptibility of bats to viral infections compared to other mammals.

### Natural killer cell receptors

Natural killer (NK) cells are an important component of the innate immune response, providing a first line of defence against viruses and tumours. To our knowledge, no investigations of NK cell receptors from any species of bat have been reported previously. NK cells express cell surface receptors that recognise major histocompatibility complex (MHC) class I or class I like molecules on the surface of cells and lyse infected or abnormal cells by cytotoxicity. Two families of NK receptors that bind classical MHC class I ligands have been identified: the killer immunoglobulin like receptors (KIRs), which are encoded by genes in the leukocyte receptor complex (LRC), and the killer cell lectin like receptors (KLRs), which are encoded by genes in the natural killer complex (NKC). Different lineages of mammals make use of genes from the two different superfamilies to carry out analogous functions. KIRs are used preferentially by primates, cattle, domestic cats, dogs and pigs [45,46]. Similarly, the KIR-like receptors, marsupial immunoglobulin-like receptors (MAIRs) and chicken immunoglobulin-like receptors (CHIRs), have expanded in marsupials and chickens respectively [47,48]. Although CHIR-AB binds IgY, the ligand for the majority of CHIRs is unknown and the presence of a charged transmembrane residue and a cytoplasmic immunoreceptor tyrosine-based inhibition motif (ITIM), are consistent with the possibility that they play a role in NK activity [49]. Rodents, horses and platypus are the only species so far described that have expanded the KLRs, represented by the Ly49 family [50-52]. In the bat transcriptome dataset, no transcripts with homology to KIRs or Ly49 receptors were identified. In bony fish, novel immune type receptors (NITRs) which contain an N terminal variable domain and a C terminal Ig domain have been identified as the primary activating and inhibitory receptors expressed by NK cells [53]. NITRs were also used to search the bat transcriptome but failed to identify any orthologous transcripts. The failure to find KIR or Ly49 like receptors in the bat transcriptome may reflect low expression levels of these genes resulting in their absence from our datasets. However, BLAST searches of the publicly available whole genome sequence of the closely related pteropid bat, *P. vampyrus* revealed no evidence of KIRs or Ly49 receptors. As this is a low coverage genome (2.63X), further work is required to determine whether pteropid bats have KIR and/or Ly49 receptors. Overall, the absence of these important NK receptors from our datasets warrants further investigation into the nature of NK cells in bats.

**Figure 5:**
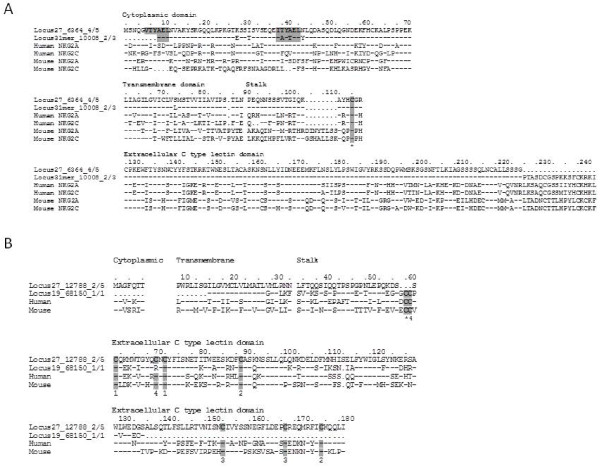
**a. Alignment of deduced amino acid sequences of bat NKG2 with human and mouse NKG2A and NKG2C.** Sequences are divided into cytoplasmic, transmembrane, stalk, and lectin domains. The predicted ITIM motifs in the cytoplasmic domain are shaded. The conserved cysteine residue in the stalk predicted to be involved in interchain disulphide bond formation with CD94 is shaded and indicated with an asterisk. Dashes indicate similarity and dots indicate gaps. **b**. Alignment of the deduced amino acid sequences of bat CD94 with the human and mouse orthologues. Sequences are divided into cytoplasmic, transmembrane, stalk, and lectin domains. Conserved cysteines predicted to be involved in disulphide bond formation are shaded. Cysteine pairs are indicated by identical numbers below the cysteine. The cysteine predicted to form a disulphide bond with NKG2 is indicated with an asterisk.

NK cells in a wide range of mammalian species additionally express CD94/NKG2 (also called KLRD1/KLRC) lectin-like receptor heterodimers. Unlike the KIR and Ly49 receptors, which bind (classical) MHC class Ia ligands, the CD94/NKG2 heterodimer binds the (non-classical) MHC class Ib ligands HLA-E and Qa-1 in humans and mice respectively [54]. The CD94/NKG2A heterodimer generates inhibitory signals whereas the CD94/NKG2C heterodimer generates activating signals within NK cells. Both CD94 and NKG2A were identified in the bat transcriptome, however NKG2C transcripts were not identified, possibly reflecting the low abundance of transcripts of this gene in our datasets. Two and 37 NKG2A transcripts were identified in the thymus and pooled datasets respectively and six transcripts corresponding to CD94 were identified in the pooled dataset. Two of the longest NKG2A sequences were aligned with NKG2A and NKG2C sequences from human and mouse. As shown in Figure 5a, the bat genes display highest conservation with other NKG2A genes including the presence of conserved ITIM motifs in their cytoplasmic domains, designated by I/V/L/SxYxxL/V indicating that they are likely functional inhibitory receptors [55]. The more divergent NKG2D, which binds MHC class I chain-related genes, MICA/B, and the UL16 binding proteins (ULBPs) in human [46], was also detected.

Two distinct bat CD94 contigs were identified, one of which is missing two conserved cysteines in the stalk region, the first of which forms an interchain disulfide bond with NKG2 and the second which forms an intrachain disulphide bond. The second bat CD94 sequence is missing a conserved cysteine in the extracellular domain that forms an intrachain disulphide bond (Figure 5b). The absence of key cysteines in both of the bat CD94 sequences may have implications for the formation of heterodimers with NKG2 and for the unique folding of the CD94 chain. Combined with our failure to detect KIRs or Ly49 receptors, our data may provide the first evidence for the presence of atypical NK cell responses in bats. However, confirmation of the nature of the NK response and the composition of receptors used by bat NK cells awaits further investigation.

Other NK receptors were also identified in our datasets including CD244 which acts as an activating or inhibitory receptor on human and mouse NK cells respectively [56] and the natural cytotoxicity receptors expressed by NK cells. Co-receptors including CD16 and CD56 expressed by subsets of NK cells in other species were also identified in the bat transcriptome. Identification of NK cell receptors and co-receptors provides information for the development of reagents to identify bat NK cells and paves the way for further studies of NK cell function during viral infection in bats.

### Adaptive immunity

Genes involved in the adaptive immune system, including MHC class I and II genes and T and B cell receptors and co-receptors were highly represented in both the thymus and pooled datasets providing evidence that bats have all of the components necessary to mount an adaptive immune response.

### Major histocompatibility complex class I genes

MHC class I molecules play an important role in the initiation of the adaptive immune response through recognition of endogenously-derived peptides from viruses and other pathogens. In the thymus dataset, 46 contigs had homology to mammalian MHC class I proteins, while 24 were homologous in the pooled data. Other transcripts in the MHC class I antigen-loading and presentation pathway were also identified, including beta-2-microglobulin, transporter associated with antigen processing 1 (TAP1), calnexin and tapasin. Class I-related genes were also present in the bat transcriptome dataset including CD1a, CD1b, CD1d, MR1, HFE, FcRn and ULBPs, which have a variety of immune and non-immune functions. The presence of ULBPs is consistent with the expression of NKG2D, but orthologues of MICA/B or Mill were not observed. The presence of NKG2D suggests bats should have a MIC homologue, but these may not be detected possibly due to low or tissue-specific expression. To our knowledge, these sequences provide the first class I and class I-associated transcripts from any species of bat.

**Figure 6:**
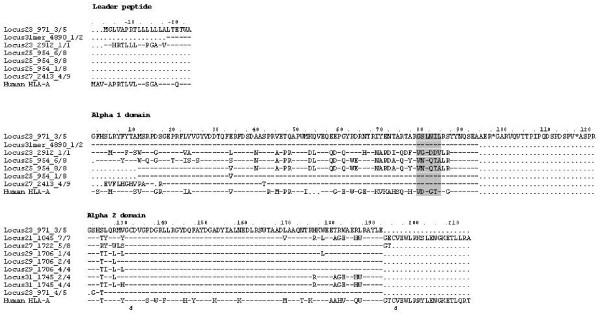
**Alignment of deduced amino acid sequences of the α1 and α2 domains of bat MHC class I loci with human HLA-A for comparison.** The shaded residues in the α1 domain correspond to a putative natural killer (NK) receptor-binding region. Other designations: d, cysteines in the α2 domains likely to form intrachain disulphide bonds. Dashes indicate nucleotide identity, dots indicate nucleotide gaps and asterisks indicate stop codons.

Of the 46 contigs with homology to MHC class I genes in the thymus dataset, 29 contained in-frame stops. These may be expressed pseudogenes, represent assembly or sequencing errors or result from reading frame shifts due to the presence of unprocessed transcript. As the sequences were obtained from multiple individuals, it is not possible to confidently distinguish between alternative isoforms, alleles and in some cases, loci. However, clustering the remaining contigs with open reading frames (ORFs), there are clearly at least 9 distinct MHC class I genes expressed. The majority of class I contigs contained the α1 or α2 domain or partial sequence corresponding to both domains and were used for further sequence analysis. The deduced amino acid sequence of contigs with the most complete α1 or α2 domains were aligned with human HLA-A (Figure 6). All of the bat class I sequences contained a unique three amino acid insertion in the α1 domain that appears to be bat specific. As shown in Figure 6, the bat transcripts display amino acid variation in their α1 and α2 domains, corresponding to the peptide binding region. However, they appear to be remarkably conserved from residues 131 to 175 of the α2 domain. These results may indicate that bats contain a very closely related class I gene repertoire that have coevolved with the specific viruses they carry.

Some of the class I transcripts represented in the thymus and pooled datasets contained an 84 bp insertion at the end of the α1 domain. The longest of these transcripts corresponded to the leader peptide through to 71 amino acids of the α2 domain and is shown in Figure 6. The insertion at the end of the α1 domain is not present in class I sequences from other mammals and includes two in frame stop codons that would prevent translation beyond the α1 domain (Figure 6). This sequence was confirmed by RACE PCR and transcripts were detected in a variety of tissues including lymph node, spleen, liver, lung, heart, kidney, small intestine, brain and salivary glands, thus providing evidence that they are not an artefact of the transcriptome assembly (data not shown). Comparison with the closely related *P. vampyrus* whole genome sequence available in Ensembl revealed that the 84 bp insertion is identical to the beginning of intron 2 of a *P. vampyrus* class I gene. MHC class I splice variants that retain intron sequence and result in the translation of a truncated protein have been identified in other mammals, including soluble splice variants of human HLA-G that plays a role in immunoregulation at the feotal-maternal interface [57]. Further investigation will be required to determine whether the bat gene encodes a soluble protein corresponding only to the α1 domain or whether it represents a transcribed pseudogene. However, given the abundance of this transcript in our datasets it is possible that it plays a role in immune regulation in *P. alecto*.

### Major histocompatibility complex class II genes

Unlike class I molecules, which are ubiquitously expressed, class II molecules are expressed only by antigen presenting and B cells and present exogenously derived peptides to T cells. The MHC class II molecules are composed of an α and a β chain encoded by A and B genes respectively [58,59]. Eutherians have three main classical class II gene clusters: DP, DR, and DQ, as well as the nonclassical DM and DN/DO gene clusters [60,61]. Sequences corresponding to exon 2 of MHC class II DRB genes have been described in four species of microbats [62-64]. However, prior to the present study no class II genes have been reported from any species of megabat. Sequences corresponding to genes involved in the class II antigen processing and presentation pathway were also identified in our datasets including the class II invariant (CD74) chain and cathepsin S (Additional file 3).

**Figure 7:**
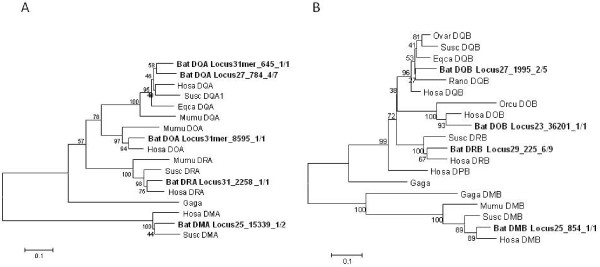
**Phylogenetic Trees of the MHC Class II Genes. a**. MHC class II A gene phylogeny based on alignment of 240 nucleotides corresponding to the end of the β2 domain through to the cytoplasmic region of bat MHC class II sequences with class II sequences from other mammals. Branch support is indicated as the percentage out of 1000 bootstrap replicates and is shown where support is greater than 50%. **b**. MHC class II B gene phylogeny based on alignment of 567–594 nucleotides corresponding to the signal peptide through to the α2 domain of bat MHC class II sequences with class II sequences from other mammals. Branch support is indicated as the percentage out of 1000 bootstrap replicates and is shown where support is greater than 50%. Species names are: *E. caballus* (Eqca), *G. gallus* (Gaga), *H. sapiens* (Hosa), *M. musculus* (Mumu), *O. aries* (Ovar), *O. cuniculus* (Orcu), *R. norvegicus* (Rano), *S. scrofa* (Susc).

In the *P. alecto* thymus and pooled datasets we identified 78 and 238 contigs respectively that were homologous to class II sequences. Phylogenetic analysis revealed that the alpha chain sequences were homologous to DMA, DOA, DQA and DRA from other mammals (Figure 7a) and the beta chain sequences were homologous to DMB, DOB, DQB and DRB (Figure 7b). These results are consistent with orthologous relationships between the bat class II genes and those from other mammals.

### T and B cell receptors and co-receptors

T cell receptor (TCR) genes corresponding to all four chains of the T cell receptor were present in our datasets, consistent with bats having both αβ and γδ T cells. Sequences corresponding to the constant and variable domains of the TCR were identified including many TCRα related contigs, TCRβ related contigs, a few TCRγ and TCRδ chain related contigs. In humans and mice approximately 95% of circulating T cells express the αβ T cell receptor. In contrast, γδ T cells account for up to 70% of circulating T cells in young ruminants, rabbits and chickens [65,66]. The low abundance of TCRγ and TCRδ related transcripts in our datasets is consistent with the possibility that αβ T cells may be the predominant TCR present in bats. In addition, a variety of T cell co-receptors, including the accessory TCRζ chain, CD3, CD4, CD8 and CD28 were identified in our datasets.

We previously described the immunoglobulin heavy chain diversity of *P. alecto*, revealing the presence of a highly diverse variable region gene repertoire [16]. Sequences encoding the variable and constant domains of immunoglobulin heavy and light chains were represented in our datasets. These included heavy chain genes encoding IgA, IgG, IgM and IgE, which have previously been described in the megabat, *Cynopterus sphinx*. No evidence for the transcription of IgD was observed in the *P. alecto* transcriptome, a result which is consistent with *C. sphinx*[67]. The two light chain subtypes, kappa and lambda and a variety of B cell co-receptors including CD19, CD22, CD72, CD79a and CD79b were also identified in our datasets (Additional file 3).

### Conservation of bat immune genes

Many of the bat immune transcripts showed high levels of sequence similarity compared to homologues from other mammals. Among the most conserved bat innate immune genes were the PRRs; the TLRs, RIG-I helicases and NLRs, which displayed >80% amino acid identity with homologues. This likely reflects their roles in the recognition of conserved pathogen motifs. Members of the OAS family were also highly conserved, in particular OAS1 which shared 87% amino acid identity with the dog OAS1 sequence. In addition, the NK co-receptor, CD56 shared 93% amino acid identity with mouse, hamster, guinea pig and human sequences. Among the adaptive immune genes, MHC associated proteins, calnexin, TAP1 and cathepsin S shared 89-95% identity with corresponding sequences from other mammals reflecting their conserved roles in the antigen processing and presentation pathway. Several members of the MHC class I and II families were also highly conserved, including CD1b and CD1d which shared 88 and 89% amino acid identity with horse and chimp sequences respectively. The bat MHC class II DOA and DRA shared 91 and 89% amino acid identity with orthologous sequences in other mammals. The T cell co-receptor, CD28 shared 90% identity with the rhinoceros CD28 sequence and the constant domain of IgM shared 92% identity with camel IgM.

### Unannotated transcripts

There were >77,000 unannotated contigs in the thymus and pooled datasets. Only about 3% of these contigs matched predicted cDNAs from the *P. vampyrus* genome sequence, which are annotated using orthologous sequences from other species [68]. The unannotated contigs contained a total of 3266 open reading frames (ORFs) longer than 300 bp. Of these, 92.6% (E-value < 10^-3^) aligned to the closely related *P. vampyrus* whole genome sequence and represent highly divergent homologues or bat specific genes. The remaining loci represent either misassembled contigs or bat-specific transcripts that are located in sequencing gaps in the low coverage *P. vampyrus* genome sequence. The 3266 long (>300nt) ORFs were searched for conserved domains using profile hidden Markov models with hmmscan (HMMer v3; http://hmmer.org/) obtained from the Pfam database [69]. This identified 345 ORFs containing 214 unique domains, including several defensins, antimicrobial peptides and DNA-binding domains. Searches using domain models from the Pfam-B database, identified a further 437 unique, predicted-conserved domains in 733 ORFs. A further 2188 ORFs remained unannotated. A high proportion of these were rich in cysteine, tryptophan and proline, and prolines frequently appeared in low complexity regions (additional file 4: Figure S2a and b). Further characterisation of these unannotated transcripts will provide insight into whether they are functionally significant and in particular whether any unique bat specific transcripts are involved in the antiviral immune response.

## Conclusion

Bats are a highly diverse, species rich group of mammals that have evolved a variety of distinctive characteristics since their divergence from other mammals [70]. Despite the central importance of bats in harbouring a variety of viruses with the potential to spillover to other species, very little is known about antiviral immunity in bats. Next generation sequencing provides the opportunity to survey genes that are conserved between distantly related species as well as to provide insights into novel adaptations through the identification of previously unidentified transcripts. To identify genes involved in the immune response, we carried out a transcriptome analysis of thymus and immune cells and tissues of the Australian black flying fox, *P. alecto*. This study represents the first survey of expressed bat immune genes and complements existing low coverage bat genome sequences. Our analysis provides a broad overview of the bat transcriptome and contains representatives of all of the major classes of immune genes. The results are consistent with bats having all of the components of the immune system present in other mammals. The majority of these correspond to genes that have not previously been described in any species of bat and thus represent an important resource for future investigations into antiviral immunity in bats.

## Methods

### Animals

*P. alecto* bats used in this study were wild caught from East Brisbane, Queensland, Australia. Bats were handled and euthanised as previously described [15]. All experiments were approved by the Australian Animal Health Laboratories animal ethics committee (protocol AEC1281).

### Preparation of tissues and cells

The thymus was removed from a juvenile male bat and immediately stored in RNA*later* (Ambion) for RNA extraction. The spleen, lymph nodes (LN), thymus, bone marrow and peripheral blood were collected from one adult male and one pregnant female bat. Single cells were extracted from the spleen, thymus and LN by tissue extrusion through a 70 μM sterile sieve (BD Biosciences) in the presence of DMEM supplemented with 15 mM L-glutamine, 100 units/ml penicillin and 100 units/ml streptomycin (Invitrogen). Splenocytes and peripheral blood lymphocytes (PBMLs) were isolated by density centrifugation over lymphoprep (Nycomed, Oslo) as described previously [21]. Cells were resuspended in DMEM with 10% FCS, 15 mM L-glutamine, 100 units/ml penicillin and 100 units/ml streptomycin and cell numbers were determined using a haemocytometer with trypan blue exclusion. The thymus and bone marrow cells were stored in RNA*later* (Ambion) for RNA extraction and the spleen, thymus and LN were cultured with a variety of stimulants.

### Culture of cells

The isolated splenocytes, LN and PBMLs from each bat were pooled and were then seeded at 1 x 10^7^ cells per well in 24 well tissue culture plates (Nunc) with PHA (10ug/ml; Sigma) and LPS (10 μg/ml; Sigma); PMA (50 μg/ml; Sigma) and Ionomycin (2nM/ml; Sigma); or polyIC (30 μg/ml; InvivoGen) and incubated in a humidified atmosphere of 5% CO_2_ in air at 37°C. Cells were harvested in RLT buffer (Qiagen) at 0, 1, 4 and 18 hours and homogenised using a QIAshredder (Qiagen) following the manufacturer’s instructions. The lysate was then stored at −80°C and total RNA extracted the next day (0, 1, and 4 hours) or processed immediately (18 hours).

### Preparation of total RNA

RNA extraction was carried out as previously described using the RNeasy mini kit (Qiagen) with removal of genomic DNA with DNase I digestion [16]. Total RNA from the thymus of a juvenile male bat was used for Illumina sequencing separately from all other samples. Total RNA obtained from the stimulated and unstimulated cells from the two adult bats was pooled as follows: 22% thymus total RNA (11% from each bat) and 78% pooled total RNA from the rest of the mitogen stimulated and unstimulated cells/tissues (~3.45% for each sample; total of 22 samples).

### Sequencing

mRNA isolation from total RNA, library preparation and single-end read sequencing was performed by Geneworks Pty Ltd, Thebarton South Australia using the Illumina Genome Analyser IIx sequencing platform. Library preparation was performed as per Illumina’s mRNA sequencing sample preparation guide (Part # 1004898 Rev. D) except 5 μg of total RNA was used for mRNA selection using poly-T oligo-attached magnetic beads. The thymus library was run on a single lane of a flow cell resulting in more than 12.5 million 65-base sequences for a total of about 0.82 Gigabases (Gb) of sequence. The pooled library consisted of 4 lanes resulting in 24 million 76 bp sequences for a total of about 1.8Gb of sequence.

### Sequence pre-processing and de novo assembly

The quality of the sequences were evaluated using FastQC [71]. Sequences were pre-processed in two stages. First, all bases at the 3’ end of the reads with quality scores of 3 or lower were removed. Second, poly A/T tails, uninformative sequences (Ns) and primer/adaptor contaminants were trimmed using SnoWhite (version 1.1.3) [72], a cleaning pipeline for next-generation cDNA sequences, which includes Seqclean [73] and TagDust [74]. We ran SnoWhite with two runs of Seqclean and one run of TagDust and a final minimal length cutoff of 20 bp was used.

The pre-processed sequences were *de novo* assembled using two different approaches. (1) The reads were assembled with velvet (version 1.0.12) [75] using individual kmers from 19 bp to 31 bp. Next, the contigs produced by velvet were processed using oases (version 0.1.15) [76]. Oases loci were then merged using cd-hit-est (version 4.0) [77] with a global sequence identity threshold of 1.0. Finally, a length cutoff was set to 50 bp and the default coverage cutoff of 3 was used. We term the final result of this process a contig (2). The reads were also assembled using MIRA 3 (V3.2.0rc3) [78] with default setting for EST and Illumina reads assembly, i.e. maximum front and end gap clip is 2 bp, maximum length of the possible vector leftover allowed is 18 bp, minimum quality score, window length and read length were all set to 20, allowed to clip poly A/T at ends, and minimum read coverage per contig was 2.

### Annotation of the conserved protein coding transcripts and untranslated regions (UTRs)

The bat contigs were firstly annotated by using the best hits of BLASTX [79] search against NR protein database and KEGG pathway database with an E-value cutoff of 0.001 for annotating the protein coding contigs that were conserved with other species. Then the unannotated contigs were further annotated by using BLASTN search against Refseq_rna database with an E-value cutoff of 10^-5^ for the contigs containing conserved UTRs and without significant protein coding regions. The contigs not annotated by the above two steps were further analysed by using BLASTN against the cDNAs from megabat (*P. vampyrus*) and microbat (*M. lucifugus*).

### Identification of open reading frames

We translated the un-annotated transcripts into protein sequences from 6 frames, extracted the ORFs longer than 300 bp. This was performed separately for the 2 datasets. These ORFs were searched against Pfam-A and Pfam-B databases to identify conserved domains. The two sets of long ORFs were pooled and clustered based on cd-hit with sequence identity of 50% [77]. The amino acid compositions were further analysed for the non-redundant longer ORFs with Composition Profiler [80].

### Annotation of the gene ontology (GO) terms

All the KEGG IDs of the human proteins identified by the BLASTX searches were extracted from the annotation process and were mapped to UniProt IDs. Then the GO analysis for the UniProt proteins (UniProtKB-GOA: gene_association.goa_human) was used to assign the GO terms for the transcripts. The number of genes in categories of the GO slim database was counted using the GO term classification counter, CateGOriser [81,82] and the immune category of the bat genes was annotated using the Generic Gene Ontology Term Mapper [83]. The GO classifications were further grouped into twelve broad categories as follows: Cell death and apoptosis (death, GO:0016265; cell death GO:0008219; apoptosis GO:0006915). Cell growth and maintenance (cell, GO:0005623; cell cycle, GO:0007049; cell differentiation, GO:0030154; cell proliferation, GO*:*0008283; growth, GO:0040007; cell homeostasis, GO:0019725; organelle organisation and biogenesis, GO:0006996; cytoskeleton organisation and biogenesis, GO:0030036; biosynthesis, GO:0009058; morphogenesis, GO:0009653; response to stress, GO:0006950; response to external stimulus, GO:0009605; protein modification, GO:0006464; response to abiotic stimulus, GO:0009628; response to biotic stimulus, GO:0009607; structural molecule activity, GO:0005198 and antioxidant activity, GO:0016209). Metabolism and energy pathways (metabolism, GO:0008152; generation of precursor metabolites and energy, GO:0006091; protein metabolism, GO:0019538; lipid metabolism, GO:0006629; oxygen binding, GO:0019825 and catabolism, GO:0009056). Translation and protein metabolism (protein biosynthesis, GO:0006412, translation regulator activity, GO:0045182 and translation factor activity, GO:0008135). Signal transduction (cell communication, GO:0007154; signal transduction, GO:0007165; signal transducer activity, GO:0004871 and cell to cell signaling, GO:0007267). Transcription and/or nucleic acid metabolism (nucleobase, nucleoside, nucleotide and nucleic acid metabolism, GO:0006139; transcription, GO:0006350; transcription regulator activity, GO:0030528; DNA metabolism, GO:0006259 and transcription factor activity, GO:0003700) Transport (transport, GO:0006810; ion transport, GO:0006811; protein transport, GO:0015031 and transporter activity, GO:0005215). Cellular component (intracellular, GO:0005622; cytoplasm, GO:0005737, plasma membrane, GO:0005886; cytoskeleton, GO:0005856; extracellular region, GO:0005576, cytoplasmic membrane bound vesicle, GO:0016023; endoplasmic reticulum, GO:0005783; extracellular matrix, GO:0005578; ribosome, GO:0005840; nucleolus, GO:0005730; extracellular space, GO:0005615; chromosome, GO:0005694; endosome, GO:0005768; cytosol, GO:0005829; nucleus, GO:0005634; mitochondrion, GO:0005739; cell, GO:0005623 and nucleoplasm, GO:0005654). Binding (binding, GO:0005488; protein binding, GO:0005515; nucleic acid binding, GO:0003676; DNA binding, GO:0003677; nucleotide binding, GO:0000166; cytoskeletal protein binding, GO:0008092; receptor activity, GO:0004872; actin binding, GO:000377; calcium ion binding, GO:0005509; chromatin binding, GO:0003682; carbohydrate binding, GO:0030246 and RNA binding, GO:0003723). Reproduction and Development (development, GO:0007275; reproduction, GO:0000003 and embryonic development, GO:0009790). Enzyme activity (catalytic activity, GO:0003824; hydrolase activity, GO:0016787; transferase activity, GO:0016740; enzyme regulator activity, GO:0030234; kinase activity, GO:0016301; protein kinase activity, GO:0004672 and phosphoprotein phosphatase activity, GO:0004721). Immune Response (immunology, immune response, GO:0006955).

### Sequence and phylogenetic analysis

Mx protein sequences and MHC and NK receptor nucleotide sequences were aligned using ClustalX [84]. Nucleotide sequences were aligned and gapped manually using Bioedit software version 7.0.9 (Tom Hall, Ibis Biosciences, Carlsbad, CA), based on the protein alignment to retain codon positions. Based on the nucleotide and protein alignments, phylogenetic trees were constructed by the neighbour joining method [85], maximum parsimony and minimum evolution using the MEGA4 program [86].

The GenBank accession numbers for sequences used in the sequence and phylogenetic analysis are as follows: MHC class I: (CAP58485) HLA-A; MHC class IIA: Human, *Homo sapiens* (Hosa) DMA (NM_006120), DOA (M26039), DQA (M26041), DRA (NM_019111); Mouse, *Mus musculus* (Mumu) DOA (M95514), DQA (M21931), DRA (U13648); Horse DQA (L33909); Pig, *Sus scrofa* (Susc) DQA (NM_001130224), DRA (NM_001113706), DMA (NM_001004039); Chicken, *Gallus gallus* (Gaga) B-LA (AY357253); MHC class IIB: Hosa DQB (M20432), DRB (NM_021983), DOB (L29472), DPB (M57466), DMB (U15085); Rat, *Rattus norvegicus* (Rano) DQB (X56596); Mumu DMB (U35332); Horse, *Equus caballus* (Eqca) DQB (L33910); Susc DQB (AY102478), DMB (DQ431246), DRB (AY191776); Sheep, *Ovis aries* (Ovar) DQB (L08792); Rabbit, *Oryctolagus cuniculus* (Orcu) DOB (XP_002714654); Gaga B-LBII (M29763), DMB (AB426148); NKG2A: Hosa (NM_002259), Mumu (NM_001136068); NKG2C: Hosa (NM_002260), Mumu (AF106010); CD94: Hosa (AJ000001), Mumu (AF030312). Mx1: Rano (NP_775119.2); Eqca (NP_001075961.1); Orca (XP_002723805.1); Susc (BAD11809.1); Bota (NP_776365.1); Ovar (P33237.1); Mumu (NP_034976.1); Hosa (AF135187_1); Mx2: Rano (NP_599177); Eqca (XP_001491517); Susc (BAF76735); Bota (DAA33026); Ovar (AAW51454); Mumu (NP_038634); Hosa (NP_002454); Avian Mx: Gaga (NP_989940).

### Analysis of repetitive or low complexity regions

Low complexity regions in protein sequences were detected with the seg program with default parameters [87].

### Quantitative reverse transcription PCR

The transcription of a bat MHC class I gene was examined using quantitative PCR (qPCR) as described previously [18]. Briefly, total RNA was prepared from lymph node, spleen, liver, lung, heart, kidney, small intestine, brain and salivary glands using the RNeasy mini kit (Qiagen) as described above. cDNA was generated using a Quantitect reverse transcription kit for RT-PCR (Qiagen). qPCR primers were designed using Primer Express 3.0 (Applied Biosystems) with default parameter settings (5’-ACGACTCCTATTCCCCAGGATAG-F and 5’-GAAAGCCACTGGTACCTGTGAGA-R). Reactions were carried out using EXPRESS SYBR® GreenER^TM^ qPCR Supermix Universal (Invitrogen) and an Applied Biosystems 7500 Fast Real-Time qPCR instrument.

## Competing interests

The authors declare that they have no competing interests.

## Authors’ contributions

Conceived and designed the experiments: L-FW, HF, MT, MLB, CC. Performed the experiments: MT, VJ, JN, GC. De novo assembly and annotation: Z-PF. Analysed the data: Z-PF, ATP, MT, MLB. Contributed reagents/materials/analysis tools: HF. Wrote the paper: MLB, ATP, Z-PF, MT. All authors read and approved the final manuscript.

## Supplementary Material

Additional file 1 **Table S1.** Summary of additive multiple-kmer velvet/oases/Mira3 assembly. Click here for file

Additional file 2 **Figure S1.** Overview of the bat transcriptome. The distribution of 178,554 and 285,268 transcriptome sequences that have mapped to human orthologues from *P. alecto* thymus and pooled tissue datasets based on GO slim terms. Sequences within the three areas of Gene Ontology: molecular function, biological process and cellular component are further divided into subgroups at the GO Slim level. Click here for file

Additional file 3 Sequences of all genes described in the manuscript. Click here for file

Additional file 4 **Figure 2.** Amino acid composition of large unannotated ORFs. The horizontal axis shows amino acids sorted by flexibility index [88].**a**. Amino acid composition of 1656 large unannotated non-redundant ORFs relative to proteins in the SwissProt database [89]. The amino acids Trp, Cys and Pro have twice the abundance in unannotated ORFs compared to SwissProt proteins.**b**. Amino acid composition of 1195 low complexity regions in unannotated ORFs relative to 1656 unannotated non-redundant ORFs. Prolines are abundant in low complexity regions, but Trp and Cys are not. Click here for file
