# Supplementary material for: The immune gene repertoire of an important viral reservoir, the Australian black flying fox
Source: BMC Genomics. 2012 Jun 20;13:261. doi: 10.1186/1471-2164-13-261 (PMC3436859; doi:10.1186/1471-2164-13-261)
Supplement: Additional file 1 — Table S1. Summary of additive multiple-kmer velvet/oases/Mira3 assembly. [file 1471-2164-13-261-S1.pdf]

Summary of additive multiple-kmer velvet/oases/mira3 assembly

	SRA: SRR350710.3 (Thymus)			SRA: SRR351237.2 (Pooled)		
	velvet additive 21-31mer cov>1	Oases additive 19-31mer Cov>3	Mira3 Cov>2	velvet additive 21-31mer cov>1	Oases additive 19-31mer Cov>3	Mira3 Cov>2
**# contigs /loci**	956,834	247,909	497,582	740,279	313,641	780,325
Longest **transcripts** (kb)	4.8	11.8	8.0	1.3	8.9	7.5
N50	164	1244	131	122	733	103
Coverage 1~3	64.8%	0	/	51.7%	0	/
% reads used in assembly	73.5	74.0	81.6	48.2	50.0	69.8
