# Supplementary figures and images for: The immune gene repertoire of an important viral reservoir, the Australian black flying fox
Source: BMC Genomics. 2012 Jun 20;13:261. doi: 10.1186/1471-2164-13-261 (PMC3436859; doi:10.1186/1471-2164-13-261)

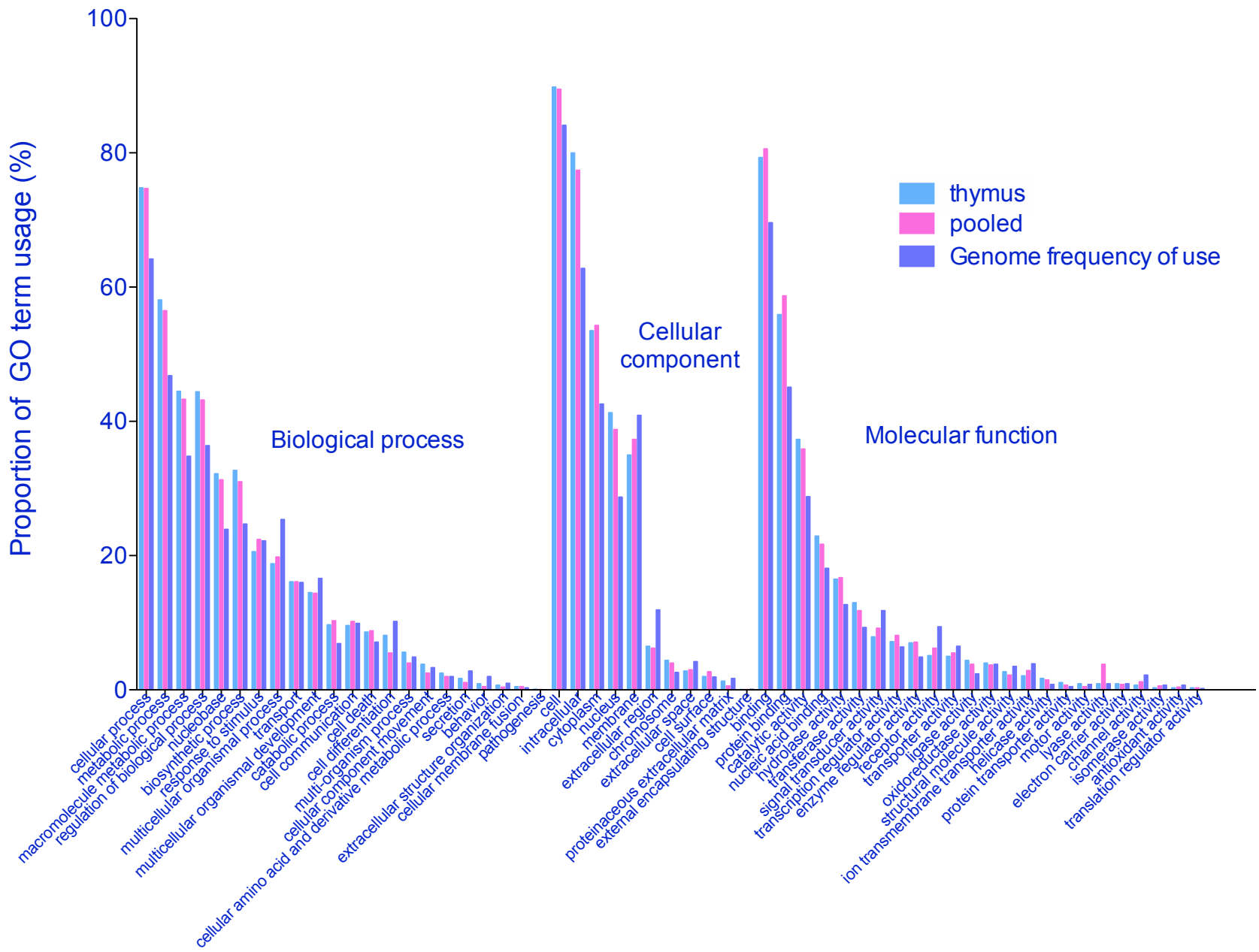

Supplement: Additional file 2 — Figure S1. Overview of the bat transcriptome. The distribution of 178,554 and 285,268 transcriptome sequences that have mapped to human orthologues from P. alecto thymus and pooled tissue datasets based on GO slim terms. Sequences within the three areas of Gene Ontology: molecular function, biological process and cellular component are further divided into subgroups at the GO Slim level. [file 1471-2164-13-261-S2.pdf]

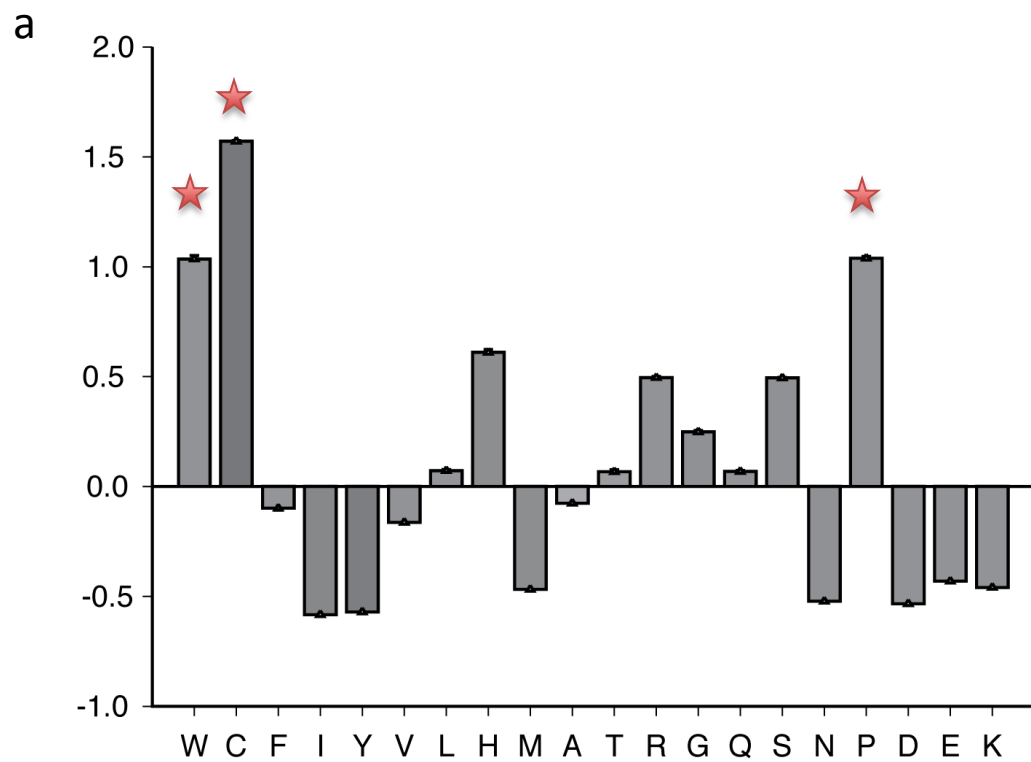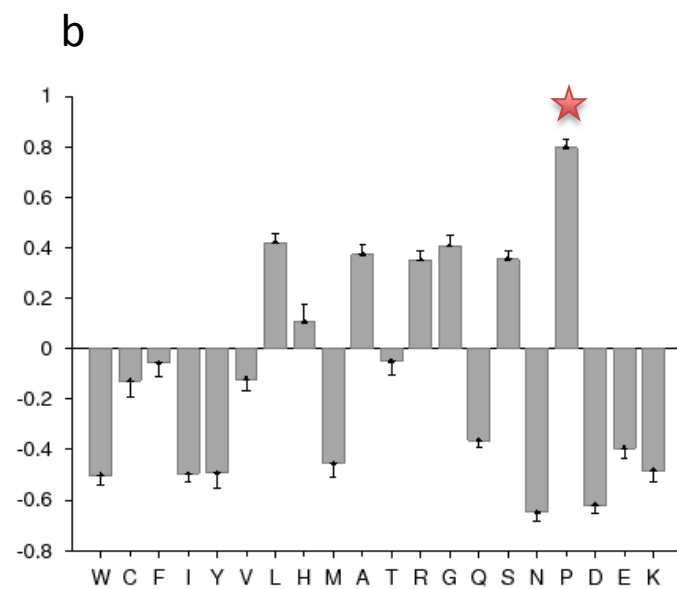

Supplement: Additional file 4 — Figure 2. Amino acid composition of large unannotated ORFs. The horizontal axis shows amino acids sorted by flexibility index [88].a. Amino acid composition of 1656 large unannotated non-redundant ORFs relative to proteins in the SwissProt database [89]. The amino acids Trp, Cys and Pro have twice the abundance in unannotated ORFs compared to SwissProt proteins.b. Amino acid composition of 1195 low complexity regions in unannotated ORFs relative to 1656 unannotated non-redundant ORFs. Prolines are abundant in low complexity regions, but Trp and Cys are not. [file 1471-2164-13-261-S4.pdf]
