# Supplementary material for: The immune gene repertoire of an important viral reservoir, the Australian black flying fox
Source: BMC Genomics. 2012 Jun 20;13:261. doi: 10.1186/1471-2164-13-261 (PMC3436859; doi:10.1186/1471-2164-13-261)
Supplement: Additional file 3 — Sequences of all genes described in the manuscript. [file 1471-2164-13-261-S3.docx]

**Innate Immune genes**

**Pattern recognition receptors**

>Locus25_26898_Transcript_1/1_TLR1

CTCTTCCGATCTTTGATCTCCATTCTAACAAAATAAGGAACATCCCTAAAGATATCGCCG

GTCTAGAAGATTTGCAAGAACTCAACATTGCTTCCAATGATTTAGCCCACCTTCCTGGGT

GTGGTAGCTTTAGCAGCCTTTCTGTACTGATCATTGACAATAATGCAATTTCCAACCCAT

CCGCTCATTTCTTCCAGAGCTGTCAGAAGGTTAAGTCCGTAAAAGCGGGGAACAATCCAT

TCCATTGTACATGTGAGCTAAGAGAATTTATCCAAAGGATAGGCCAAGTATCAAGTGAAG

TGGTAGAGGGTTGGCCTGACTCTTACAAGTGTGACTATCCAGAAAGCTATAAGGGAAGCC

TACTGAAGGACTTTCACGTGTCTCCGTTATCCTGCAGCACAGCTCTGCTGATTGTCACCA

TTGGGGTCATAGGGCTGGTGTTGGCTGTTACTCTGACTGTCCTCTGCATCTACTTTGATC

TGCCCTGGTATCTCA

>Locus21_15649_Transcript_1/1_TLR2

GCTCTTCCGATCTCAGTTTAGTAGTTTTAGTAAAACAAGTCCTCAAATGACAGTACATTC

TTGCGGCTTCCATACAATTGTGGCACAATGGACTGAAATACAACTACTGATACAAAGACC

ACCAGCAGACCAAGACTGGCCTTTAATGAAGGAGCTTAGGACTTTATTGCAGTTCTCAAA

TTTAACCAAAACCCCTCCTGCTGAGCTTCGTCGGTGGGCCACTCCAGGTAGGTCTTGGTG

TTCATCATCTTCCGTAGCTTACAGAAACGCTGGGGAATCGTCTTTTTCTCAATAGGCTCC

AGAAGAATGAGAATGGCAGCATCGTTGTTCTCATCAAAGAGACGAAAATGGGAGAAGTCC

AGTTCATACTTGCACCACTCACTCTTCACAAAGTTTTCAGAAAGCACAAAGACAGTTTTG

TGGCTCTTTTCGATGGAGTCAATGATATTGTCAATAATCCACTTGCCAGGAACGTAGTCT

CGCTTGTGAAGACACAGCTTGAAGGGAGGGTCGAAGTGCTCCAGCTCCTGGACCATCAGG

TTCTCCACCCAGTGGGAATCTCGTTCACTGTAAGACACGAAGGCGTCATAACAGATGTCC

CTGTGGGGAGCTTTCTTGGGCTTCCTCTTGGCCTGGAGCCAGGCCCACATCATTTTCATG

TACCACAATCCATGGAAATGGTGGCACAGAACTACAGTGAGCAGGATAAACAGGAAAAGG

GCACAGCACAAGGCAGACACTAGAGCTGTTCTGTGGCACTCGGAAATGGAGAGGTGAGTG

TCCTGAACACGGTGGCCCCGCACGTGGGACGGAGAGTCACAGAGGTAGTTTCCCGGCCAG

TCGATCAGGATCTGGGCCAGTGCCTGCTGCTCCTGCATGAAACGCAGGAATTCACAGGAG

CAAATGAAATTGTTGCCCCCAGCTT

>Locus23_72674_Transcript_1/1_TLR3

GCCAATTTTACAAGATATAGCCAACTTACTGTCTTGGATGGAGGATTTAATACCATCTCA

AAACTGGAGCCAGAATTGTG

>Locus19_14363_Transcript_1/1_TLR4

CAGGTGGTTCCTCACATTACCTACCAGTGCATGGAGCTGAATCTCTACAAAATCCCCAAC

AACATCCCCACATCAGTCAAGAAACTGGATCTGAGCTTTAACCCCCTGAGACGTCTAAGC

AGCCATATCTTCTCCAACTTCTCAGAATTGTGTGAAATTGAGATGATTGAAGATGATGCA

TATGAGGGTCTAAACCATCTCTCCACCTTGGTATTGACAGGAAACCCTATCCAGAGTTTA

GCCATGGGAGCCTTTTCTGGACTATCAAGTTTACAGACACTGGTGGCTGTGGAGATAAAC

CTAGTGTCTCTAGAGGACTTCCCCATTGGACACCTGAAAACCTTGAAGGAGCTTAATGTG

GCTCACAATCTTATTGATTCCTTCAAGTTACCTGAATATTTTTCTAACCTGTCCAACCTG

GAGCACTTAGACCTTTCCAATAACAAGATCCAAACTATTTGTCATAAAGACCTACAGGTT

CTACATCAAATGCCCCCATTCAAACTCTCTTTAGACTTGTCCCTGAACCCTTTAGACTTC

ATCCAACGAGGTGCCTTTAAAGAAATTAAGCTCCATGAACTAACTTTGAGAAGTAATTTT

AACAGTACAGATGTAATGAAAACTTGTATTCAAGGCCTCGCTGGCTTAAAAATCAATCGT

TTGGTTCTAGGAGAATTTAAAAATGAAAGAGCCATAAAACATTTTGACAAATCTGCCATG

GAGGGACTGTGCAATTTGACCATTGACGAATTCCGGATGACATACTTCGATGACTTCTCA

GAG

>s4_c280927 TLR5

GTCGCTGTACTGAGCGTCCAGGTGTCTGAGCAAAGCATTGTGCACCCACTCGAAGTCCTT

GCTACTGAAGCACAAATAGGCATCGTATGTGTACGTGTCGGAGCCTCTTCCCAGGG

>Locus19_29564_Transcript_1/1_TLR6

GACACGTGAAAGTCCTTCAGTAGGCTTCCCTTATAGCTTTCTGGATAGTCACACTTGTAA

GAGTCAGGCCAACCCTCTACCACTTCACTTGATACTTGGCCTATCCTTTGGATAAATTCT

CTTAGCTCACATGTACAATGGAATGGATTGTTCCCCGCTTTTACGGACTTAACCTTCTGA

CAGCTCTGGAAGAAATGAGCGGATGGGTTGGAAATTGCATTATTGTCAATGATCAGTACA

GAAAGGCTGCTAAAGCTACCACACCCAGGAAGGTGGGCTAAATCATTGGAAGCAATGTTG

AGTTCTTGCAAATCTTCTAGACCGGCGATATCTTTAGGGATGTTACTTATTTTGTTAGAA

TGAAGATCAAGAATCTTGACCCTAGGAGGTAAACATCTGAAAACAGATTCAGTAAGTATA

TTTGAAGACAAATTTAACATCACTATACTCTCAACCCAAGTGCAAGTTCTGTTATGTCTA

GTATATTCTAAAGAATTCCAGCTCACATCCAGTATTTCCAAAGATGGCATATCCTTAGTC

ATGAGACCCACTTTGAAAAGATCTTT

>Locus19_44463_Transcript_1/1_TLR7

TTTTGGGAAAGATCTAGTTCCCTAAGTTTTCTAACATTTTTAAACCATCTTTGGGGCACA

TGCCGAAGGGAGTTGCTGTGTAGACGTAAAACTTGTAATTCTGTCAATGCATCAAAAGCA

TTTTCATGGATCTGTAGGGGAGAATTATTTTCACAGGGTGTACAAGGAAATGGGACATTA

TAACAGCGAGGGCAGTTTCCACTTAGGTCAAGAACATGCAGTTGAGTGAGGTTATTAAAA

TCATCCTCTTGGATTTTTGCAATGATATTGTTGTAAAGATAGAGTTCTGTTAAAGTGGGT

GGCAAAACAGTGGGGACAGCTGAGATCG

>Locus19_29550_Transcript_1/1_TLR8

TTCGAGAATGGATGCATAAAAATCTACAGGTTACAATTCCTCGACTGACAGATGTCACTT

GTGCCAGCCCTGGGGATCAAAGAGGGAAGAGTATTATGACTCTAGAGCTAACCACCTGTG

TTCCAGACACCATCGCAGCCATATTGTGTTTCTTTACATCCTTCATCACCATCATGGTTA

TGTTGGCTGCCCTGGCTCACCACTGGTTTTACTGGGATGTTTGGTTTATATATCAAGTGT

GTTTAGCGAAAGTAAAAGGCTACAGGTCTCTTTCCACATCCCAAACTTTCTATGATGCTT

ACGTTTCTTATGACACCAAAGATGCCTCTGTTACGGACTGGGTGCTAAATGAGCTGCGCT

TCCACCTAGAAGAGAGTGAAGGGAAAAACGTGCTCCTCTG

>Locus19_21161_Transcript_1/1_TLR9

CCGATCTGGACAGGTCCACATGCCCCAGGCTGGGGAACAGCCCGAAGATGTGGAGCTGGG

CCTGGCTGATGAAGTTCATCTGCAGACTCAGGTTGTGGAGCAAGGGCAGGCGGGTCACGG

ACCGCAGCGTGTCCTCGCCTAGCGAGCGGAAGAAGAGGCCGTTCATGTTGAGCTCCTGCA

GGGCCTGCAGGCTCTCGAAGGAGGCTGCCAGACGCAGGTGGGCATAAGACACCCTCTTGT

GGTAATTGAAGGACAAGTTGAGTTTGCGCAGCTGCGTCAGGCCCCTGAAGGCTTTGGTGT

ACATGATGCAGTCGTACAGAAAGTTCTCACTCACGTCCAGCACCGAGAGGTTGCCCAGGC

CGCGGAACCAGTTGATGTCCAGCCAGCGGAGAGAGTTGTCCTTCAGCACGAGGCCTTCGA

GGTGCCGCAGGTGGCTGAAGGTGTCGGGGTGCAGGCTGGGGGAGCCCTTGGGGCACTCGA

CGCAGGGGTTGCGGGCGTGGTCACAGCGGCGACAGTTCCCGCCCACGTCGAGCACGCGCA

GGGCGGTGAGGTTGGCCAGGTCCTCGGGCCCCAGGGAGACGATGCGGTTGTAGGACAG

>Locus21_18912_Transcript_1/1_TLR10

TAATCTAGAGAAAGAAGATGGTTCCGTCTTAATTTGCCTTCATGAGGGAAACTTTGACCT

TGGCGAGACTGTTACTGAAAATATCCTAAACTGCATTGAAAAAAGCTACAAGTCCATCTT

CGTATTGTCTCCCAACTTTGTCCAGAGTGAGTGGTGCCATTATGAACTCTACTTTACCCA

TCACAATCTCTT

>Locus25_51326_Transcript_1/1_TLR13

ACTGCTTTGGCTCAGGTCAAGGGATAAAAGCCTGGTACAGTTGGCAAAGTGCTTTATGCC

TCTTGTGTCAGTGGCATTTTTCATAAAAACCAAAACCTCTAATATGGGCAAGTTTCTGAG

GAGGTG

>Locus23_1350_Transcript_1/2_RIG-I

CACATGGATTCCCCAGTCATGGCAGCAGTCTTGTCTGGCACAGAATATCTTTGCTTTCTT

CTCGAAACTCCCAAAATTCTTTGGTTTGGGGTGCAATTTACTCACAAAGCACTTCTTGAA

AGCATCTCCCACCACAGTGTAATGGCAGTCCTCAACCACTCTTATATCAGCTGTATAACA

AGCAAAGGCTTTGCACTTTCTGCAGAGAAGTTTTTTATTTTCCTTATCAAGTATAGGTTT

TACTTTACTTTGACTATCCCTGATGAATTTTTCATGAATCTGAATCTGGCGAACCTTTTC

TTTAAATACTGCTTCATCCCATGTCTGAAGTCTTAAAATGGAGTCATTCATCATTTTTTC

TTTGTATATGTTTATTTTTTCTTTTTCAATTACATCAGAATTACTAGTCAGAAGGAAGCA

CTTGCTACCTCTTGCTCTTCCTCTGCCTCTGGTTTGGATCATTTTGATGACATTGCCCAC

GTACTCATACAAGATGACCAGATTGCACTGAGCAATGTCAATGCCTTCATCAGCAACTGA

GGTGGCAATCAGAATCTTGTTGTCTCCACTGGTTTTGAATGTATGCAATACATCTTTCTG

TGCTGGGAGGGTCATTCCTATGTTCTGTTTTGTTCTGCCACGTCCAGTCAATATGCCAGG

TTTTAGAAAGCTGAGTTTAGGATTTTCTTCTATCCATTTCTTTAAAGCATCCACAAGTGC

TCTGGTTTTCACAAAGAGAATGGTTCTAGTCTCTGGGTTTAAGTGGTACTCCTCTTGCAA

GACGAAGCAGAGGTCTTTGAGTTTAGGGTTCTCGTTGCTGGGATCCATGGAAATGCTTTC

TAGTTCTTGCAGCTTTTCTTCAAATCTCCGAGTGAGATCTTGCTCAGTCTCATCAAAGCC

TGCTGTTCGGACATTACGGAAGAAGTCTTTCAAGTAATCCAGAGCATCTTTCATCCGTGC

ATGCTCACTGATGATGAGGGCATCATTAAATTTCCGCAAATGTGAAGTGTACAAAAACAG

TGCTTTACAAATTCTGCTCTCTTCATCTTTATCTGGCATCTTTAACACCATGCACTTTTT

CTGAACTGCAACAATCCACTGTTCATATTTCTGTGTGCCAAAATTCCTGTTTTGAATCTG

AGAGAAGTTTCCGAGAGTTATGGTACCAAGTGTGTCAAAGATACTCTTTGCTAGACTCTC

TGTCTCCTTCATCAGCTGAGAGATGATGCATTTAAATCTGTCAGTAGTCCGTGATTCCAC

TTTCCTGAAAAACTTCTGGGGCTTATAAACAACTTCCTCCAGTTCCTCCAAGTTGTCTTT

GACTGTTGCTACCACTGATATGTCAAGAGAAGCACACAGTTTACAGATATATTCCAAGGC

TTCACCTGTGCTTTTGGCATCCCCAACGCCAACCGAGGCAGTCAGCCCAACGACCTGAGG

CAGTGAGTCTGAAGATCCTCCAAGTTTCTGATCTAGATAATTAAACATGATCATATTGTA

AGGATGATGTTTACTGGTGTTGTGGCACTCGTCAAATATCATCAAAGTAAAGACTGAGAG

TGATGGAACAGTCCCATTTTTAAGGCTGTTCACAAGAATCTGTGGAGTTAAAATGATGAT

GTCATTGTCCTCAATAATCTGTTCCACGGAGACATTCTCAGCTGTTGCTCCAGAAATGCC

TGAAACTTTGTACCCGCGTCTTTCAAAATATTTTGAGAACACAGATTTCTGCTGTTCATA

CACTGGAACTTGAACAGCAAAAAACACAACCTTCCCTTTTTGTCCTTGTGGAAATTTTTG

AAGATGATGTTCACATATAAGAAGTGAAACAAAGGTTTTTCCACAACCAGTAGGAGCACA

TATTATTGTGTTTTTTCCGTTCTTAGCAGGCAAAGCAAGCTCTAGTTGGTAACCTCTTGG

CTTCATTGGGCTGTGAGTATGAGATACTTCTGAAGGTGGACATGAATTCTCACTAAGATT

CTGGCATTCTGGTTCTTCCTTGTAGAAAATCTGTACTTCGGAAGTTTTCATTTCCTCATC

CTCAAGGTCTTGCAATGCAACATCTTTTGCACCTTTCTCTGTAATCCACAGTTCACTGAA

CTTGCTTTCTTCTTTCTCCAAAGCAAGTTTCAAAGATTTGGGCCAGTTCTCCTTGTCTGA

TCTGAGAAGGCATTCAACCATTTTCTCTGCACCAGCCATCGGCCCCTTGTTAGAACAAAT

CTGTGTAATTTCTTCACATTCCTGATCAAGTAAACATTCAGATATGTCAGGAAGGATATC

ACTTGGGTTAATTCTGGTTTTAAATTCTGGTTGCAAACGCCTTAAAAGCAATCTATACTC

CTCCAACTTTTCAATTTTTTGGAAATCCCAATTTTCAATGGCTTCATAAAGGCCAGAATA

ACCTGCATGGTTAAGAGCATCCATAAATCCACGGAACCAGCCTTCCTCCTGGAGCTCCAA

TAGGAACCTGAGAAAAAGTGAGGCAGCCTCTACTGTACCCTTGTTGTTTTTCTCAGCCTG

AATATACTGCACTTCATCCTCCTTAAACCAGGGGGCCATGTAGCTCAAGATGTAGGTAGG

GTCCAGAATCTTCGTGACATAGTCCCGGAAAGTGTACAGATTCTGCCGCTCCTCGGCCGT

CATGCTCGCAGCCTTACCTGCGCTCCTCTCCCTTTAAAGCCCGCTGTGAGCCGGCAGTAT

CTGTACACTTTCCGGGACTA

>Locus19_14498_Transcript_1/1_MDA5

GCAAAAAGAGCATCCCCTGAGCCAGAACTGAACCTCAGGCCTTACCAAATGGAAGTTGCC

CAACCAGCCTTGGAGGGAAAGAATGTCATTATATGCCTCCCTACAGGGAGTGGGAAAACC

AGAGTAGCCGTTAAGATTGCCAAGGATCACTTGGACAAGAAGAAAAGAGCATCTGAACCA

GGAAAAGTTATAGTCCTTGTCAATAAGGTACCATTAGTTGAACAGCTCTTCCGCAAAGAG

TTTCAACCATTTTTGAAGAAATGGTATAATGTCATGGGATTAAGTGGTGATACTCAATTG

AAAATATCATTTCCAAAAGTTGTCAAATCCCATGATGTTATTATCAGTACAGCTCAAATC

CTTGAAAACTCCCTTTTAAACTCAGAAAAAGGAGAAGATGATGGTGTTCAGTTGTCAGAC

TTTTCTCTCATCGTCATTGATGAATGCCACCACACCAATAAAGAAGCAGTCTATAATAAC

ATCATGAGGCGTTATTTGAAACAGAAGTTGAGAAATAATAAGCTCAAGAAAGAAAATAAA

CCAGTAATTCCCCTACCTCAGATACTGGGATTAACAGCTTCACCA

>Locus19_36710_Transcript_1/1_LGP2

GTGGGAGGGGGGTCAGAGGGTGAGCTGTGGCCCTTCAGCTGCTTCCTCAGTGTGGGAGTC

AGGTAGCCTGGGCGCATGAGCTGGTGATTCAGCCAGGAGGGGTGTGCACCCAGGTTTTGC

TCAGAAGACAGTAGCTGTTGTGGATTCTCCTGCCCCCTGCAGCCTGAGCCAGGCACTGCA

GTGACAAGATGGTTAGGCCAGGGAGAGTTCTGACAGGTGCTGGGCACAGTGCTGCACGAA

GTTGAAGTCAGGCACGGGAAAGGGCACGCGGGACCACTTCTTGGGCTGGACCCGCCCTTG

TGGCGTCTCCAGCAGCATGCTGTGGACTTTGAGCACTGGCAGGTTCACCGACTTGTAGAT

CATCTGTAGACCCCATAGTTCCCCACAGTTCTTGCAGCTAATGGTGCCTCCAGGCCTCCA

GTCCTTGAAGACTTTGTCTATGACCACAGGCTTCTGCGAGACATTGTAGTAGATCGAGAA

GTTGGGGTTCACGTTGACATGGTGGGTACCCTCCACCTTCCGCAAGTCGCTCCCATGGCC

CACGGCCACCATGCAGTTGATGCAGAGGAGCTGCACCTGCTCCGCCAGGAACTGCTGCCG

TCTACTCTCCTGCTGGGCTGCCTGGGCTGCCCTCTTGACCAGGGCTGCCCTCTGCAGATC

TCGGATCTTGGCCTGG

**Interferons, Interferon receptors and interferon stimulated genes**

>Locus27_54452_Transcript_1/1_IFNA

ATCCTCCTTCCAACTGCTGATCCGTTCCAGTGAGGAATTTTAGCTCTTTATGCTGGAGAT

CACCACATGCAAGAAGAGGGGGCCTGAGATTTCTGGACCTGGCAGCCATCCAGCTCCCTC

TGACAAAACTTGCAAACATTCTTGTCCCTGAGACAGAAAACAAGCAAGATTCTCCCCATT

TGGTGCAGAAATGTGAAGCTCTCCTGCTTTGGGAGGCCATGGTTCTGGGCTAGGTCACAA

ATAAGGATGCAGACAGAGTTGAAAAACATGATTCTCG

>Locus31_278_Transcript_1/1_PKR

GACGATCTTTGATCAGGTTTCTTCTTAGAAGAACTTAATAAATACCATCAGAAGAACAAT

GTAGAAATTGAGTATCGTGAACTGTCTAAGAGAGGACCTCCACATGACTTAAGGTTTACC

TTTCAAGTTGTAATAGGTGAGAGAGAATTTCCAGAAGCTGAAGGTAAATCAAAGAAGGGA

GCAAAAAATGCTGCAGCCAAATTAGCTGTCGAAATACTTAATGAAGAAAAAGAGGAAGTT

AGTTCTTTATCACTGTCGACAACAGATACTTCAGATTTATCCATTGGGAATTATGTTGGC

CTTGTTAATAGGATTGCCCAGAAGGCAAAACTACCTGTAAATTATCAGTTGGGATTAGGT

GCAGGTGAACCCGGAAGATTTTATTATACATGCATAATTGGACAGAAAAAATACGACGTT

GCTGTGGGTTCTACTAAGCAGAAGGCAAAACAACTGGCTGCTAAACTCGCATATGAACAG

ATACTATCAGCAAAAACCTCAGTGGATGACTTAGCTTCTCTTGGTTCTCCCTCTGCTGCG

TCCAGTGACTATGGAAGTAACTCTTCAATGACAAACATGTGTGATTCTGAGTCACCATGT

GAAAATGGCTTTTTAGCAAATGGCTCAGAAAGAAATGATAACAGCGACAGTTTTAGCAAT

TTGTCTTCACCTTCTGTGAGCAGTGTTAGAAAAAGTCTTGGGAAGACGAAAATTTTTAGA

AATTTGGCACCTAATTTTAACTTGGTTGGAGCAGCAAGAAATGAGTACACTATGGACCAC

AGGTTTCTCGAGAATTTTCTAGAGATAACATCAATTGGCTCAGGTGGATATGGCCAAGTG

TTCAAAGCAAAACACAGAATTGATGGGGTGACTTATGTTATTAAACGTGTTAAATATGAT

AACGAGAAGGTAGTGCGTGAAGTAAAAGCTTTGGCAGCGCTTTCTCATCCAAATATTGTT

CGCTACTATAATTGTTGGGATGGAATTGATTATGCTTCTGAGGACAGCGATAATGGAAGA

TTCGAATCATGCACTAAGTGCCTTTTCATCCAAATGGAATTCTGTGAGGAAGGGACATTG

GAACAATGGATTAACAAGAGAAGAGACCAGAAAACGGACAAACATTTGTCTTTGGAATTA

TTTGAGCAAATAGCAAAAGGAGTGAATTTTATACATTCAAAAGGGTTAATTCATAGAGAT

CTTAAGCCAAGTAACATATTTTTAGTAGATACAAAAAAAATAAAGATTGGAGACTTTGGA

CTTGTAACATTCCTGAAAAATGATGAAAAACGGACAAATAACAGGGGAACTCCACGATAC

ATGAGCCCAGAACAGCTTTCTTCAACAGAATATGGAAATGAAGTGGACATCTATCCTTTA

GGGCTAATTCTTGCAGAGCTTCTTTACATATCCCGCACTCTTTTGGAAACATGTAAGATT

TTTGAAGATCTAAAGAAAGGCAAGGTCTTAGATGTATTTGATGACAAAGAAAAAATTCTT

CTACAGAAATTACTTTCAAAAGACCCCAAGAAACGACCTAACACATGTGAAATACTGACT

ACTTTGGAAGAGTGGAAGAATGTTGCAGAGAAAAAGAAACGAAACACATGTTAGAGCCTT

TCTAAAAAAGTATCCTGCTTCTAATGTTTGATTTTCCTGTAACTCTCTAAAATCTGCTAG

GGACTATCAATGGTATTTACCTGCTATTTTCATTTTCCCTTTGATTGATTCATTAATTAA

CATAAAAAAATGAGGATTTCCAC

>Locus23_3341_Transcript_1/4_Mx1

CGGCGCGCGGCGGCGGGGTGCTGGCCGGAGACAGGAAGCGTAAGAGAACAGCTCTGCATT

TCCATCCAACTCATCAGCGTTACATCGTCAAGTAAAAAGAAGGTGTATTAGAGCTGAACC

GATAAGGGAAGAAGATGAATCCTCCTACAGTGAAGAGGAGTCCTCTTAAAGTGAAAGTCA

AAAATCATAATTTGGCCTCTGCGTCCAGTCCTCGGTTACCAGATGCTAAGGTGTCTGAGG

AGAATCTGGAAAAAGGCACCGAGAGCAGCCTGTGCAGCTACTACGAGGAGAAAATCCGCC

CCTGCATCGACCTCATCGACTCTCTGCGGGCCCTGGGCGTGGAGCAGGACCTGGCCCTGC

CCGCCATCGCGGTCATCGGGGACCAGAGCTCGGGCAAGAGCTCCGTGCTGGAGGCCCTGT

CGGGCGTCGCCCTGCCCAGAGGCAGCGGTATCGTGACGAGGTGTCCTCTGGTGCTGAAGC

TGAGAAAACTGAGGGACGGGGACGAATGGAAAGGCAAAGTCAGTTACAAGGACCAGGAGA

TTGAGATGTCAGATGCTTCGGAGGTGGAAGAGGAAATCAGGAAAGCCCAGGATGTCATTG

CTGGGGTAGGAATGGACATCAGCCATGAGCTGATCAACCTGGAGGTCAGCTCCCCTCACG

TCCCAGACCTGACCCTCATCGACCTCCCCGGCATCGCCAGGGTGGCCGTGGGCAATCAGC

CCCAGGACATCGGGGCACAGATCAAGGCGCTCATCAGGAAGTACATCCAGAGGCAGCAGA

CCATCAACCTGGTGGTGGTGCCCTGCAACGTGGACATCGCCACCACGGAGGCCTTGAGCA

TGGCTCAGGAGGTGGACCCCGACGGAGACAGGACCATAGGAATCCTGACGAAGCCCGACC

TGGTGGACAAAGGCACCGAAAACAAGGTTGTCGACGTGGTGCGGAACCTGGTCTGCCACC

TGAAGAAGGGCTACATGATCGTCAAGTGCAGGGGCCAGCAGGACATCCAGGAGCAGCTGA

GCTTGACCGAGGCTCTGCAGAAAGAGAAGGCCTTCTTCGAGGACCATCCGCATTTCAGGA

ACCTCCTGGACGAAGGAAAGGCCACGATCCCCTGCCTAGCAGAGAGACTGACCACCGAGC

TCATCGCACACATCTGCAAATCTCTGCCCCAGTTAGAAAATGAAATAAAGGAGAAACAGC

AGAGTATCACAGAGGAGTTGCAGATGTATGGCATGGAGACGCCGGAAGAAGAAAACGAAA

AAATGTTCTTTCTGATAGATAAACTTAATGCATTTAATCAGGGCATCCTCACTTTAATCC

AAGGGGAGGAAATCGTGGAGAAGAGCAACACTCGGCTGTTTACCAAAATCCGAAAAGAAT

TCAACAACTGGAGTAAAGTGATTGAAAACAACTTCCAGGACGGTTATGATGCTGTATACA

ACGAGATCTGCAAATTCGAAAATCAGTATCGTGGCAGAGAGCTGCCAGGATTTGTGAACT

ACAAGACATTTGAGAATATCATAAAACAGCAAATCAAAACTCTGGAAGAGCCCGCTGTGG

ATATGCTGCACACCGTAACTCAAATGGTTTGGCTCGCCTTCACAGAAGTTTCCCAGAAAA

ATTACAGTGAATTTTTCAACCTCTACAAAGTCTCCAAGTCCAAAATCGAAGACATTAAAA

CCGAACAAGAAAGAGAAGCCGAGAAGTCCATCCGACTACACTTCCAAATGGAGCAGATCG

TGTACTGCCAAGACACGATGTATCAGAGATCGTTACGGAAAATCAGGGAGAAGGAAAAAG

AGAAGGAAGAAGAAAGGAGGAGAACATTAGGTCGGACGATCTGCGAAGAGAGTTCTTCGT

ATAACTCCCTAGATGAAATCTTTCAACACCTGATAGCCTACCACCAGGAGACCGGCAACC

GCCTGTCCACCCACATCCCCTTGACGATCCAGTTCTTCGTCCTTCAGTCATTCGGCCAAC

AGCTGCAGAAGGCCATGCTCCAGCTGCTGCAGGACAAGGAGCAGTACGACTGGCTCCTGA

AGGAACGCAGTGACACCTGTGACAAGAGGAAGTTCCTGAAGGAGCAGCATAGGCGGCTGG

TGCAGGCTCGGCACCAGCTGGCCAAGTTCCCCGGTTAGGTCGGGCTCCGTCCCCGCCGCG

GCCCCACCGCACAGGTCGCGCCCGTCCC

>Locus21_139_Transcript_4/5_Mx2

GCCCCCACCCCCGCTTTAGCAGGAGAATTTGTAGAGCGCGCGCCGAGCCTGGGTGAGCCG

GTAGATTTTCTCCTTCAGGAACTGCCTCTTGGCCGCTGTCTCGCTCTGCTCCTGCAGCAG

CCACGTGTAATGCTCCCTCTCTTGCAGCATCTGCATCATGGCCTTCTGCAGATAGTCGCT

ATTCTCTTGGAGCATAAAATACTGGATGATGAACGGGATCTGGTTGGCCAGGCGGTTGCT

GGTTTCCAAGAAGTAGGCATTCAGGTGCACCCCGATTTCAGTGATGGAGGAAACCGAAGC

CACGTCATTCGATAAAGGAAATTGCAACGGGGCAGTCTTGCCAGGCGAATTCGCGTCTTC

TCGGGCCTTTTCCAGAAGCCTGCTGTAAATCTGGTCTTGACAGTAGACCTGCTGCTCCAT

CCTGAACTGCAGGCGGATCACGGAGTCCGCGGTTTCCGCCCGTTTGATTTTTATTTCTTC

GATCCTGCTCTGGACTGTTTTATTAAGGTTGGAAAATTCGCCAAAATGGTTTCTGGCTGT

GTCGACGAAAGTTTGCCGGACGATTTCGACAGCCTTCTGCAGCATTTCCAGGGCGGGGTC

CACCAGCTGCTGCAAGTACTGCTGCACGATGATCTCGAATGTCTTGTAGTTGACAAACCC

CGGAAGCTCTTTGCCGCGATACTGCTTTTCGTATTTTGAAATTTCTTGGTGAACCGTATT

TTTAACTTTTTGGGTGTTGGCTGCGAGGACCAAGACCCAGGCTTTAAACTCCTCTCTGAG

TTTGTTGTATAAGCGAGTCTCTTTCTCCTTTACGACTTCCTCTCCTTCTACCAGCTTCTC

AATGTCCCGATTAAACATTTTAATTTTCTCAATCAGAAAGAACATTTTGTCGCTGTCACT

GCTGGGGATGTGCTCCCCGCACTGGCGCAGCTCGTCCGTCGCACTCTGGTGGCATTCCCG

TATTTGACTTTCCAGTAACGGGAGCGATTTAGTGATGTGCGTGATGAGCTCGATGGTAAG

TCTTTCCGCCAGACGGGGCACTGTGGCCTTTCCTTCCTCCAGGAGGGCTCTGAAATAGGG

GTGTGCCTGGAAGAACATCGTTTCTTTCCTGGTGGCCTCCGCCAAGCTCAGCCTGTCTGC

GACCTCCTGCTGGCCCCGGCACCTCACGATCATGTAGCCCTTCTTGAGGCGGTACGTGAG

GTTCTGCACCACGTTCACGACCATTTTTTCGGTGCCCTTGTCCACCAGGTCCGGCTTGGT

CAGGATCCCTATGGTCCTGTCTCCGTCGGGGTCTACCTCCTGAGCCATGCTCAAGGCCTC

CGTGGTGGCGATGTCCACGTTGCAGGGCACCACCACCAGGTTGATGGTCTGCTGCCTCTG

GATGTACTTCCTGATGAGCGCCTTGATCTGTGCCCCGATGTCCTGGGGCTGATTGCCCAC

GGCCACCCTGGCGATGCCGGGGAGGTCGATGAGGGTCAGATCGGGGACCTCGGGTGAGGT

GATCTCCAGGCTGATGAGCTCGTGGCTGATGCCAACACCGTTCCCGGCTATGATGTTCTG

GGCTCTGTGTATTTCCTTCTCCACCTGCAGCGGGGCCTGGATCTGGAGCTCCGTGTCCCG

GTAGCTGATCTTCCCGCTCCACGGGGCCTCGCACGAGAGCTGCTTCTTCAGCTTCAGCTC

CAGCGGACACCTGGTCGTGATTCCGCTGCCTCTGGGGAGGGCGACGCCCGACAGGGCCTC

CAGCACGGAGCTCTTGCCCGAGCTCTGGTCCCCGATGACCGCGATGGCGGGCAGGGCCAG

GTCCTGCTCCACGCCCAGGGCCCGCAGAGAGTCGATGAGGTCGATGCAGGGGCGGACCTT

ATCCTCGTAGTGGGCGAACAGGCTGCTCTCGGGGCCCTTCGTGTTTTCTTGGCTCCAATT

TTCTTCCGGCAGCGGAGGGTTCAGCGTCAGCGAGTTGATGTTCTTGCAGAGGTTAACGGG

GTCCTTGTCTCCCACCGACACGTTTGGGGGCATTTTCACGTGCCCTCTTGCTGAGGCGAA

GAGCAGCGAGTGTTGCTGGAAGAGATTCATTTCCTTTTTCGGACAGTGTTGGGAGGGCGC

CTGATTGTGCCTCTGTTGCAGCAAAGGCTTGTGGGACTTTGGCATTAGCCGGGAACCAGC

TGGGGCGGGGGGCGCCTTTCCCGCTGCTCGTCACTTCCGGAGATCG

>Locus21_52331_Transcript_1/1_Confidence_1.000 OAS1

CTTCTTGTAGTTCTGGACCAATTTCAAGACTGTCTGAAACCCCTGAGCTGTTCTGAAATA

CGTTTCCTTGTTTCCACGTTCCCAAGCGTAGACCGTCAGGAGCTCCAGGGCATACTGCGG

TGGCAGGGGCTTCCCAAGCTTCTCCTTACACTTTTGGTACCAGTGCTTGACCAGGCGGAT

GAGGCTCTTGA

>Locus25_6313_Transcript_1/1_OAS2

CGACAGCTTTATGGAGCTGATCCAAGAAAGTTTTATTGGGCTGGAGAAAGTCCTTGATGA

ACTTATCCAGAAGATGGCCTGGGGTCTCGGAGAGCGGTGCTGGCAGAACATTCCAAGATG

GTCCAGGGGACTCGTTCGAGCTGAGATAATTCAACCAGACTTGAGCTTCTCCATTTAGCA

GTTCCCAGCATGCCTTATCTTTGCTTACGTTATTGGTTGGGTCAGTTGGATCCAAGATTA

CTGGCCTCGGGGATCGGATCTGGCCCAGTATGATGTTCCGGACCGTCTCATCCTCAAAGT

TGTAGTTGAC

>Locus23_31186_Transcript_1/1_OAS3

GGCGGCTGTCAGGGGGGCGAGCACGCGGCCTGCTTCACAGAGCTGCGGAGAAACTTCGTG

AACACTCGCCCATCCAAGCTGAAGAACCTGATCATGCTGGTGAAGCACTGGTACCGCCAG

GTGTGCCCACGGGAGTCGAAGAAGGAGATGCCCCCAGCCTATGCTGAAGAACCTGATCCT

GCTG

>Locus21_22853_Transcript_1/2_OASL

CTTCCGATCTGTCGAATCTTCTCTTTAACCTTCTTTATGGACTCATAAGGGTTCACCCTG

AGGATCAAATCTGGGTAACCCCACTGCTCCACTGTCACCTGGATGTCTCGTGCCCTCTTT

ATGTTCCAGCTGGGGATTGGGTTCTCCTTGTCATCATAGCAACAGTCCTGTTTCAGGCAC

TGGTTGGCTCTCTGTGCGACTATGTCCCATCTGTATCCTTTCGCCACGTTGTGGGTGGGG

TCAGCTGGATCCAGGATGATGGGCCTTTCTCTTTTTAGCTGTTTTCTGACAAAGTCCTCG

ATGACTGGGTTCTGGAATGTGTAATACTTGGTCCAGTAGATACAGAGTAAGTCATACTCC

TGGAGCAGTTCCATCACAGTGGTGAGGCCTTCCTCCAACCTGAAATTCTTATCCTCCTGT

GTGCCCATTTCCCAGGCGTAAATGGTCAGTAGCTCGAGGGCATAGAGAGGGGGCAGCATG

GCCCTGGGGCACTTGGCTTTCACATACTGCAGGTACCAGTGTTTTACCAGCCGCAGGAGG

CTTTTCAGCTTGGTTGGCTGATGTTTGACGAAGTTTCTCTGCAGCTCACAGAAGGATGCA

GAGAAATTTGCAGGGTAACTGTTGCAGGCCTCGATCAGGCTCTCATAGACCACAGGATGT

GGCTGAGAATTGGGAATTGAATGTCCCATGGCCCTATAGGCAGGCACAATGGTGACAGTG

ATGGGCTCTGCAGTCTTCATAGTCTGAATGGTGAAGACAAAAGCATCAGGCGCTCCCTGG

ACCACTCCTATCACCTCGAGCCCAAGGGCCACCAGGTCATGGCAATGCCACAGCTTTTTC

CATATCAATCTCAAAACAGCGTGGTGGTGCTCGGCCTCCTCCTGGAAGCTGTGGAAACAA

CTCAGGAACACCACCAGCTCCACCTCCGTGCTGCTCCTGAGCGCTGTGCCATTCCCGAAG

GAGCCCACCTTGACCACCTTTAGCACCCGCACTTCCTGGTCCAGCCCATGTTCCCTCTGG

AAGGGCTCTTCCCTCAGGAACTGCTCCACAGTCCGCACAGCCTCCAACACCTCATCTTTC

CACTCCCGGCTGGGCTGTAGCCACTGAGCCACGAAGGA

>Locus29_19480_Transcript_1/1_ISG15

GAAGATCGGGGTGCCCGCCTTCCAGCAGTTCCTGGTGCCCCTGACGAATTCCATGCTGCT

AGCGGACCTGAAGCAGCAGATCACGCGGAAGATCGGGGTGCCCGCCTTCCAGCAGCGCCT

GGTCACGCACCCGGCCGGAACGGTGCTGCGGGACGGGGTCCCCCTCGTCGGGCAGGGCCT

>Locus19_72234_Transcript_1/1_RNaseL

ATCTTTACCAGATCGGAATTCCTCTTTTCCACTGCCAGAATCAGGGGTGTCTTCCCTTTC

TCTCCCCTCACATTGACATCAACCCCGTGGTCTAGCAGAAGGCGAACGATGGCCTCCATT

GTCTCCT

>Locus31_88_Transcript_1/1_IFNAR2

GGAGCGGCCACGCGAAACTGCCAAGATGTGACCGTCAAGAAAGGACTTATAAAATAACAA

AGATGCTTTTGAGCCAGAATGCCTCTTTGATTAGACCACTTAATTTGTATCTCATGGTGT

ACATTAGCCTCGTGTTTGGTACTTTGCAAGCTTTGCCTGATATTTCAGATGAGCCTTGCA

TTTTCGAGATAACGTTAAGAAATTTCCGGACAATTTTATCGTGGAAATTAAAAGACCACT

CCATTGTACCAACTCACTATACATTACAGTATGCGATCATGAGTGGACCAGAAGATGTCA

TTACTGTGAAGGACTGTACCAATATCACAAGGTCATTCTGTGACCTGACAGATGCGTGGG

TGAACATGTCTGAGACGTACACTCCCAGAGTAGTTGGACAACGAGGGAACAGGACGCTGG

TCGATTGTGAGGGCAGCTTATTCCCGTTAATGGATATGCCTTTGGAACCGCCAAATTTTG

AGATTGCTGGTTTCACCGACCACATTAATGTGACAGTGGAGTTTCCACCCGTCCTGCCCA

AGATAGTGTATAGCGAAGGATCATCGTTTTACTTGTCACTTGTCATCGAAGAGAAGTCAG

GGAACATTGTTAAGAAGCATAAACCCAAAATCAATGCAGACATCGCTGGGAATTTCATTT

ATGTCATCAACGAGCTAATTCCAAACACAAACTACTGTATATCTGTTTATTTTGAGCCTT

CCAATCTGGGAACAATAAATAGATCACCTGTAAAATGTACCCGTCTTCAACCCGAACAGG

AATCAGAGCCATCAGAATCTGCCAAAATAGGAGCATTGATTACTCTGTTTTTGATAGCAG

CTGTCATCATGAGCACCATAGTAACACTGAAACGGATCGGTTATATATGCTTAAGAAATG

AGTTCCCCAAAATGTTGAAGTTTGATAACTTCTCAGCCTGGATATTTCCTGAGCTGCCAC

CGCTGGAACTGGTAACCGTGTTGGAGGTCATTCCCATCAACAGAAAGAAGAAAGTATGGG

ATTATAACTATGATGAAAGTGACAGCGATGACGAAGCAGCCACCCGAGCAAGTGCAGGAG

GCTATACCATGCACGGACTAGCAGGCAGGACTCTGTGTCCGGCCTCCACCTCTTCTGCCA

CCTTGGAGGGCTGCAGCGACCCAGACGCCCCAGACGCCCCAGACGCCGAGGAAACTGACT

CGCCCGAGCCTGAGGCTGAATCTGAGTCTCTGATGGCACCAGGACCTGAGTCAGAATGCA

CATGTGAGAATGCCTACGAGAGGAGAGAGACTGTGCTGACGGACCTCTTTTCAGAGGAGG

ACAGCAGCTCCACTAAGGGATCCAGGGACAAAACTATCTTCAATGTGGATTTAAATTCTG

TGTTTATGAGGGTCCTCGACGACGACTCAGAAGTCCCTCCAGTGTTATCTCTTCCAGAAG

AGACAGTCGACCTAGAGGATCCCGAAGAAATGGAAACAAGCCTTCTGGTGGCCAGTGGAG

AAGGGTCACAACCATCCTTCCCCAGCCCCTCTGTGGAGTCCTGGTGGCATGAAGATACTC

CTTCTGAAAAAAGTGACACTTCCAAGTCTGATGTTGACACTGGGGATGGTTATATAATGA

GATGAATCCCAAAATATTGAATTAACTTGGACTGACGGATACCTCCAGGGTTATCTTTCC

TGTACCCTCACCCGCTCCTGCGGTCTGCAGGTGTTCTCTGGGGGAAGGACTTGGAGACCG

CAGAGTCGGCCCCATTTTTCCTGATGACATCACCTGTGCGAATTCCCAGTATGGGGGAAG

GAGAGGCGGGAGGGACACCTAGGATCAGTATAATTCTGTGC

>Locus31_1515_Transcript_1/1_IFNAR1

CCCTAGTGTTGGTCGCCGGCATGTCGTGGGTGTTGCCCGCAGCCGCAGGAGAAACAAACT

TAAAATCTCCCCAAAATGTAGAGGTCTACATCATTGATGACACCTTTATCCTGAAGTGGA

ACCAGAGCGATGAGTCTGTCGGGAATGTGACTTTTTCAGCCGATTATAAAACGCCTGGGA

TGGATAATTGGGAAAAATTACCTGGGTGTCAAAATATTACTAGTACGAAATGCGACTTTT

CTCCACTCAAGATAAATGTTTTTGAAGAACTTAATTTGCGTATAAGAGCAGAAAAAGGAA

ACAATGTGTCTTCATGGTCTATAGTTGACTCATTTATACCAATTAAACAAGCTCACATTG

GTCCTCCAGAAGTACATTTAGATCCTGAAGATAAGGCAATAATAGTAAACATCTCTCCTC

CTGGAACAAAAAACAGTACCATGTGGGCCATGGCTAGTACAAGCTTTATATACAGCGTAG

TCATCTGGAAAAAGTCTTCAAGTGTAGAAGACAGGACTGAAATTGTTATTCCCGGAAGTA

AAATTAATAAACTCTCACCAGAGACAACTTACTGTTTAAAAGTTAAAGCAGGACTACGTT

CACAGAGAAAAGTTGCTTTTTATAGTCCAGCGTATTGTGTAAATACTACAGTTGAAAATA

AACTGCCTCCACCAGAAAACATACAAATCAGTGCCGAAAATCAGGTCTATGTTCTTAAGT

GGAATTACACGCATGAAAACGTGACTTTTCAAGCTCAGTGGCTCCATGCCTTTATGACAA

AGATTCCTGAGAACTATTCAGGAAAATGGAAACAAATACCGAATTGTGAAAATGTCACAA

CTACCCAGTGTGTCTTTCCCCAAAATGTTTTCCCAAAAGGAATTTACTTTATCCGTGTCA

AAGCATCTGATGGAAATAACACATCCTTTTGGTCTGAAGAGGAAAAATTTGATACTGACA

GACAAATTGTCATACTTCCTCCAGTCATCGACATGAAGCCCATTAACAACAACTCGCTGC

GGGTCTGTTTCGGCACCCCAAAAGGCTCTCAGAACAAGTCTGTGAACCAGCATTTCCCAC

TAATTTATGAAATTTGGTTTTGGGAAAACATTTCAAATGCTGAGAGTAAAATTGTAAAGG

AAAGAGCTGATTTTACTTTTCCCAACTTGAAACCGCTGACTGTATATTGTATCAAAGCCA

GAGCACTGATCGAGACTGCAAAGTGGAATAAAAGCAGTGTTTTCAGTGATACTGTGTGCG

AGAAAACAAAACCAGGAACCACTTCCAAAACCTGGCTTATAACTGGAATTTGCGTCGCGT

TTTTGATCCCAGTTGTTCTTTATGTTGTGAAGGTCTTCTTGAAATTTATCAATTATGTGT

TCTTTCCATCAAGTAAGCCTCCTTCCACTATAGACGAGTGTTTCTCCGAACAGCCGCTAA

AGAATCTATTTTTTTCCACTTCTGAGGAAGAAACTGAAAGATGTTTTATAATTGAAAATA

TAGACACTATTTCTGTAGTAGAAGAAACTAATCAAATTGATGAAAATCACAAAATATATA

ATTCCCAAACCAGTCAAGATTCAGGAAACTATTCGAATGAAGACGAAAACAGTGGAAGTA

AAACAAGCGAAGACTTTCTACAGGAGGAAACTGAATGAACAGAAACGAGCTCTGTTAGAG

GGTTTCGTAGAAGTTATACTCGGAGCCTGAGCTCCTTGCTTCTCTCAGTAACTATCAAGA

GGATATTTGCTTGTTGGAGAAGAAAAAAACATCCGATCATAGGTCCTAAAAACACAGACA

ACCACTTACGACAAATGAAATTACAGGAATTTGGAAAAAAGTCTTCCACATTCTTTTCCC

TGTGTAATGTCTTATAAGACCTGTCGTTGGACCTGTCTTATTTAGATGAGATGAGTTAAA

TCCGAAAGATGCCCACTGTCGTTCTGATGACATGTGAAGTGCCACTGCTGTTTGTGGCGA

AAAGTAGTATTGTGTTCGATCAGTTAATGTCACGATGCTGCTGGTTGCTGCATGTGTCAT

GGATTCTCCATTCCCAATATTGCTCATTATTTTTCAGAAGATATTTAGAACATTTGGTCT

TTTCTTTTGATGCTAAAGCAGACCCTTAGGAAATTCTTTAGAAAAGCTGAATACCACTGC

CACTGAAGAAATTTTCTGGAAAATAGCTTGCTCTAGAAAACATCTGCAGATGCCAGAAAC

ATAGTCCTTGGGATTTGTCAGGGGAATTCTGTCAATTTGTCCCAAGAATATTGGCTCATA

CGTCATAGTAACCAGGTAATCTTTTACTATACATTAAGACTTCCTGATTCATAACTTATA

GAATCACTTCTGATTAGAATTTGCAATCATTGAATTTATAGAAAAATGGAAAATTGTCTA

ATAAGAGAAGTTACTAGTTTTAAGATGTGTAAGATGTTAGTGACACTGTATGCAAGAAGC

CTTGGTACCAGGAAATGCTACCGGTAGAAAAGACACAGACAGAAGTAGAATAATTCACTA

GGAAAGAAATAGGTGACTTTAGTGAAAAATAAGAATAAGGTGTATAAGCAGCTTTTAATT

ATTAACATGGGATTGTTTGTTTTTATGGCTTACCTGTCATTTTTAACATGAAGCCAGCTT

CTCTGCACTGTCCAGCAGTATTCCACTCTACCTGCATGTACTCAAGACAGTGCAAATTAA

TCAAGGTGAGGGGGAAAATCTAAGTGGGAAGAACACCTAATTTAAAAAATTATATGATGC

TTTTCAAAGGCACCTATAGCTTATTTTTATACTATTTGATATTTGGGGTTATTCGTGGAG

ATCCTTCACAGGAAGGAAGCCTAGGAAATCTAGGGCAAAATTGTCAACTTCAGTGGTTGC

CATAGTGACCAGAGAAAGAAGAAGGAGCTGCTCAGAGCATGGTGGGTCATGTGTTTACCG

AGGAGTCTCCGCTGAGGTCAGTTCCATCCGGGTGGGAAAAAAGGTGAAGGCAGCTGCCTG

GGGGAGGGCTGTGACACTCAGCGGGACCCTAGCATGCTCGCAGCTCTGCAGGGCCCAGCG

TGCGCTTTCCTGGACTTGTGGTCCAGGCTCCTCACAGGCATCATGGGGACCAGGGAGGGT

CTCCCCTTCTGAGACACAGGTCTCTGCTGGAGACTCCCTGTAGTGCCCAGAACACACTCA

ACCTTTTGAACTTTTTGACCAGAAACGTTTAGTTTAAAGCAGACCTTCCGGGTTGGCCCT

TCGTTTGGAAAGGCCTTGTCTGTGGAAATGAGCTTCTTTAAAGAGGACGTTAAGAGCGTG

GCATGACACCCTGCCACCACTGGCTTTCATGCAGTGCCGTCTGTCTAGCACACAGACCCT

CGATTTACCACTGGGAAAAAGCTGCTTCAGACTGGGTATCCTAGAAAGAAACGTTCCCTG

TGTGAGATGTGAAGGAGGGGAAACTCAGACGTTAAAAGAGACCAATGAAATCGTTAGGGT

GCGAAGACATGGGAGCACAGGTCTTCAAGTTGTAAGACCTCCCTGGCTTTCAAGGAACGT

GTATTTGGCGGCAGCAGCAGCAGCAAACAGGAAAATCCTCACTTTCACCTTGTCTTCCAG

ACAGGAAGTGCAGTTCAAGTCCGTTCTTCCTCATGGTCCACATGTAGCTTAATCAGATGC

GCTTGCTTGTAAGGTGGTCTTTTTTTGAAGAGTACTTGGAAGTACCGTGTCCTTGAGAGC

CTCCACATCACACTATTGATAGTTCTTGGAGAGGCTGGGTGTAACCTAAGAATTTATGTA

GAAGATCTACATCATTACAGCCAGAACTAATGTTACATTAATATGTAGTGTAGATTAATA

TGTGTGTCATAGTGACGGGTCTGTGAACACATCCGTGCGTGCTTACACCTACTACACATG

TATGCACTTGTGATACGGGCCACGTTCCATTCGTATAGCCATGAAGTACTTAGTATAGAC

TCTAGTTAGAAAATTAGAAATGCTGCTCAGAACTGTGAACTGTAGAGTCTGGCCAGAATT

TTGTAACCTACAAAAAACTTTTAAAATACAATTTGGAATTTGTATCTTTTTTTCAGGCTG

TGAGTTTTAAAGCAGCCTCAGATAACACTTAAGAATTTGAAGCATGTACAGAACCTTTCC

TTTCTCCGCCTTCATCCTCGTTCCCTTTGACTACCTGACATGATAGGTATTCGTTTTACC

TGGTGTCTGTCTGCCCCCAGGAGACTGGGAGCCCTATGAGAGCAGGGACTTTCTTGTATT

CATTACTGTGTCCCCAACAGCCGCAACAATGCGGTGCCTTCCACATAGAACATGCACCGA

GAATAACAAAGGAATGAAAGACAAAAATTTGAACAAAGTAGGTGTTTCTGACAATGTGTA

TATGTTTACTGAGTTGTGGGAGGACATAGTGTTTTCAGTGTTTCTGTTATATCTTCTATT

TCATTTGTATTTTTGTCTTAAACCCTATTGCTGTTAACAGTTTTATATAATAAACATCGG

GGCCA

>Locus21_7877_Transcript_2/2_IL10R2

TCCGATCTCCAGCCGGCGCTTGGCGGAGCGAGCGCGCGCGGTGGGGTGTGGGGGGTGCCT

GGCTCCGGCCCGCGCCCCTCGAGCGCCCGCCCGGGCTCCCCGCGCGGCCATGGCGCGCAA

CCGCTGGAGCTGGCTGGGCAGCTGCCTCCTGGTGTCAGCATTAGGAATGGTACCGCCTCC

TGAAAACGTCAGAATGAATTCAGTTAATTTTCAGAACATTCTCCAATGGGAGTCGCCTGC

TTTTCCCAAAGCGGGTCTGACTTTCACAGCTCAGTACCTAAGTTACAAGAAATTCCAGGA

TATGTGCACAAGTACTGCCCTGACGGAATGTGATTTCTCAAGTCTTTCCAAGTATGGCGA

CCACACCTTGAGAGTCAGGGCCGAACTTGCAGAGGAGCACTCAGACTGGGTGAACGTCAC

CTTCTCTCCTGTGGATGACACCATTATCGGACCTCCTGGAATGCAAGTAGAAGCGCTTGC

TAATTCTTTACATATACGTTTCTTAGCCCCCAAAATTGAGAAGGAACATGAAACATGGAC

CATGAAGAATATTTATAACTCATGGGCTTATAATGTGCAATATTGGAAGAACGGCTCTGA

TGAAAAGATTCTAGTTACTTGTCAGTATGACTTCGAGGTCCTCAGAAATCTCGAGCCGTG

GACGACTTATTGCGTTCAAGTTCAAGGATTTCTTCCCGATCGGAACAAAACCGGGGAATG

GAGTGAGCCCGTCTGTGAGCAAACCGTCAATGACGAAACAGCCCCCTCGTGGATCGTCGT

GGCCATCATCCTCACGGCCTCGGTGTGCGTGGCCTGCCTGCTCCTCCTTGGCTGCTTCGC

CCTGATGTGGTGCATTTACAAGAAGACCAAGTACGCCTTCGCCCATGGGAACAAACTTCC

ACAGCACCTGAAAGAGTTTTTGGGCCACCCTCATCACAGCAGGCTTCTGTTTTTCTCCTT

CCCCCTGTCTGATGAGAACGACGTTTTTGACAAACTGAGTGTCGTCACAGAAGTCTCTGA

AAGCAGCAAGCAGAATCCTGGGGACAGCTGCAGCCTCAGGACCCCAGCTGGGCAGGGCTT

GCTCTCCAGCTAGCTTCTGAGGAGCGGGGCACACTCTGCTGGGTACAGCGACCCCGCTCT

TCTCACGGCTGCCTCTGAGAGTGATCAGAGCAAAAACAAGGGCCGAGACCACCTGAGCAA

GCCCCGGTTGACATCTAAACAACCAAAGAGCTAGATTTTAAAGACTCGCTTGGCAAAAAT

ACTCCACCTTGGGAATTTGCCTCTTTATAAAGACTTTAATGATTAAAAACTAGTTGGCCA

CTAAGGGTATAATTTTCATCCTCTTATGTTCACTAATGTAAAGAATAAGTTTTAATGTGA

AAAATAAAACTATTTAACGTGATGAATAAAACCAAATGCTGTAAAATCTGACCAGACTAC

CAATAGACTGATCCAAAAAGCAAACTCAGCCAAAAATTCATCAGGAAACACTACACAGAG

TGCAAACTTTCGAGATATTCTCGGATACTTCAGCTGGGAGATCG

**Natural killer cell receptors and co-receptors**

>Locus27_12788_Transcript_2/5_CD94

TGAACCGCTCTTCCGATCTCCCTTCTGTCCTAAGAAACACTAAATAAGCTGTTGTTTACA

GATAAAACGCATTTGTTCCCTGCAAGGTTCATCCAAAAAACCTTCATTTGAGCTATATAC

TATGCAGTTCGATATATTTACAGTTCGAAGAAGTGAAAATAGAGTTTGGGAGAGAGCAGA

GCCATCCTCCCACAACCAGGCACTGCGTTCTTTATTGTAAGAGAGTCCGATCCAGTAAAA

CAATTCACTGATATGGTTCATGAAATCCAATTCATCTTTGTTTTGTAACTGAAGTAGACT

GGAATTCTTAGAAGCACAGAAATCTTTGCTTTCTTCCCAAGTTATCGTTTCATTAGAAAT

AAAATAACAGTTGCATTGGTACCCAGTCCACATTTTTTGGCAAGAAGAATCTTTCTGGGG

TTCTAAGTTGGGTCCTGGAGATGGTGTTTGTTGAATACTTTGTTGAGTAAATAAATTGTT

CCGCAACATTACCAAAGTAGCCATCAACACAAGGCACATTACTCCTAAGATGCCAGAAAT

CAACCTCCATGGGGTGGTCTGAAAACCTGCCATGAGAAATGGTGTTCCGAC

>Locus19_68150_Transcript_1/1_CD94

CATCTTAGGAGTAATGTGCCTTGTGTTGATGGCTACTTTGGGAATGTTGCTGAAATTCTCAGTCACTAAATCAAGTAGTCAGCCAACACCATCTCCAGAACCCACCCTAGAACCCCAGGAAGGCTCTGGCTGCTGTCCTTGTCAAGAAAAGTGGATTGGGTACCAACGCAACTGTTACTTCATTTCTAATGAAACAAAAACTTGGGCAGAAAGCAGGAATTTCTGTGCTTCTCAGAATTCCAGTTTACTTCAGTTGAAAAACAGAGATGAATTGAGTTTCATAAAGTCCAATATTGCTTTTTACTGGATTGGACTCTTCTACAATAAAGATCATCGTGCCTGGGTGTGGGAGGAGTGCTCTG

>Locus27_6364_Transcript_4/5_NKG2A

AACCGCTCTTCCGATCTCAGCATAGTCCCAGACACCAGTCAATTGCAGAGATGAGTAACC

AAGGAGTAACCTATGCAGAACTGAATGTGGCCAAGTATTCAAAGGGACAGCAACTAAAAC

CTAAGGGCACGAAAAGCTCCATCTCAGTAAGTGAGCAAGAAATAACCTACGCAGAATTAA

ACCTTCAAGATGCTTCTCAGGATCTTCAAGGGAATGACGAAAAATTCCACTGCAAAGCTT

TACCATCACCTCCAGAGAAGCTCATTGCTGGGATCCTGGGGGTCATCTGCCTTGTCTCGA

TGTCCACTGTGGTAATAATAGCTGTTATTCCCTCTACCTTAAACCCAGAGCAGAATAATT

CCTCCTCGGTAACAGGAATCCAGAAAGCGTATCATTGTGGTCGTTGTCCAAAGGAGTGGT

TTACATATTCCAACAATTGCTATTACTTTAGTACCAAAAGAAAAACATGGAATGAGAGTC

TGACGGCCTGTGCTTCTAAGAACTCTAACCTGCTTTATATAGATAATGAAGAAGAAATGA

AATTTCTGAATTCCCTATATCTTCCATCATGGATTGGAGTCTACCGTAAAAGCAGTGATC

AGCCATGGATGTCAAAAAGTGGTTCAAACTTTACACTAAAGATAGCAGGATCATCATCTA

GTCAACTTAATTGTGCTCTGCTATCCTCATCTGGC

>Locus31mer_10008_Transcript_2/3_NKG2A

TATTTTCGTTTCATGAAAAATAAAGTTACCCAACAGAATCCAAACTCAAGTGTTTTAACT

CTAAATCTTACGCTTGCAAAAAGACTTTTTTGGAGATCCACAATCACTTGCTGTGGGGCC

AGATGAGGATAGCAGAGCACAATTAAGTTGACTAGATGATGATCCTGCTATCTTTAGTGT

AAAGTTTGAACCACTTTTTGACATCCATGGCTGATCACTGCTTTTACGGTAGACTCCAAT

CCATGATGGAAGATATAGGGAATTCAGAAATTTCATTTCTTCTTCATTATCTATATAAAG

CAGGTTAGAGTTCTTAGAAGCACAGGCCGTCAGACTCTCATTCCATGTTTTTCTTTTGGT

ACTAAAGTAATAGCAATTGTTGGAATATGTAAACCACTCCTTTGGACAACGACCACAATG

ATACGCTTTCTGGATTCCTGTTACCGAGGAGGAATTATTCTGCTCTGGGTTTAAGGTAGA

GGGAATAACAGCTATTATTACCACAGTGGACATCAAGACAAGGCAGATGACCCCCAGGAT

CCCAGCAATGAGCTTCTCTGGAGGTGATGGTAAAGCTTTGCAGTGGAATTTTTCGTCATT

CCCTTGAAGATCCTGAGAAGCATCTTGAAGGTATAATTCTGTGTAGGCTATCTCCTGCTC

ACTTACTGAGGTGGAGCTTTCCGTGCCCTTAGGTTTTAGTTGCTGTCCTTTTGAATACTT

GGCCACATTCAGTTCTGCA

>Locus19_12325_Transcript_1/2_NKG2D

GAATGAGTAAATAGCACTAAACAAAGTTAAATCTCCAAAGTCATTAGAGACTTTGTCTTC

ATGTTTTATTTTTTCTCTTAGGCTCTTCTTGCCCAAAAATGTGGATTGGGTACCAATGCA

ACTGTTATTTTATTTCTAATGAAACGAAAACTTGGGAAGAAAGCAAAGATTTCTGTGCTT

CTAAGAATTCCAGTCTACTTCAGTTACAAAACAAAGATGAATTGGATTTCATGAACCATA

TCAGTCAACGGTTTTACTGGATCGGACTCTCTTACAATAAAGAACGCAGTGCCTGGTTGT

GGGAGGATGGCTCTGCTCTCTCCCAAACTCTATTTTCACTTCTTCGAACTGTAAATATAT

CGAACTGCATAGTATATAGCTCAAATGAAGGTTTTTTGGATGAACCTTGCAGGGAACAAA

TGCGTTTTATCTGTAAACAACAGCTTATTTAGTGTTTCTTAGGACGGAAGGGATGACAAG

GAGGTCCGGTGTTAATAAGAATGGTATGTTTACCTCTTATATTAGTACTAACTATCTACC

GCAGATCGGAAGA

>Locus25_4815_Transcript_1/2_CD244

TACCTCCTGGAGGTCACCAGAGAGGATGGGAGTATTTGCAGACACGAGTTCCAGATTTCC

GTATTTGATCACGTTGAGAAGCCTCGCCTGCTGGAGCAGTGGAAAGCCCTAGACAGAGGG

AGGTGCCAGGTGACCCTGTCCTGCTTGGTCTCCAGAGGTGGCAATGTAAGCTACGCCTGG

TATAGAGGGAGCGAGTTGATCCAGACGACGAGGAACCTCAGCGAACTGGAAGAGCAGGTT

GGCGTCGGAAGCCGGCACAAATACACCTGCAATGTCAGCAACCCTGTCAGCTGGGCGACC

CAGACGCTTGAACTCACCCAGGCCTGTCACCATGTCGACTTTCTTTCCATTTTGGTGATC

GTTTTGCTTCTCATACTCACCCTGACCCTCGGCAGCCTCGCCGGCTTGTGTGCGTGGAGG

AGGAGGAGGAGGAAGCGGTCACAGCCCATCCCAAAGGAATCTCTGACGATTTATGAAGAC

GTCAACAGCCTGACGACCAGGAGAAATCAAGAGCAGGAGCACAGCTTCCTTGGAGAAGAG

AGGACCATCTACTCCAGCGTCCAGGCCCAGTGGGCGAGGCCCGGAGAAGTTGGCAGAAGG

AGGAAGCTCGTCCTGGAACCCACCTCCGTGTTCCC

>s4_c417117 CD56

ACAAAGGTCGAGACGTCATCCTGAAAAAAGATGTCCGATTCATAGTCCTGTCCAACAACT

ACCYGCAGATCCGGAGCATCAAGAAAA

>Locus29_792_Transcript_3/6_CD16

TGTGGCAGCTGCTGTCACTCGCAGCCCTGCTGCTACTAGTGTTGGCCAGCCCGCAACCCG

AAGATCGCCCAAAGGCCGTGGTGTCCTTGCATCCTCAATGGGACAGGGTGCTCAGGCATG

ACAGTGTGACCCTCATGTGCCAGGGGGCCTACCCCCATGGGGACAATTCCACACAGTGGT

TTCGCAATGGAAGCAGCCTCTCCAATCAGACCTCCAGCTACGTCATCGCATCCGCCGAAG

TCGAGGACAGTGGAGAGTACAGGTGCCAGACCGGCCTGTCCACGCTCAGCGACCCTGTGC

AGCTCCAAGAGCACGCTGCCTGGCTGCTGCTCCAGGCCCCTCAGTGGGTGTTCCAGGAGG

GGGAGCCCATCCGGCTGCGGTGCCACAGCTGGAAGAACGTTACAGTGCGGAAGGTCCAGT

ATTTCCAGGATGGCAGAGGCAAGCAGTTTTCTTACGAGAACAAGAAATTCCACATTCCGA

AGGCCATGCACAGACACGGAGGCTCCTACTTCTGCAGGGGGATCATCGGGACCCGCAACG

TGTCTTCGGAGGCTGCGAACATCACTGTTCTAGGTCCGGCAGTTCCCTCCATCTCCCCAC

TCTTTCCACTGTGGCCCCAAATCACTTTCTGCCTGGCGATGGGACTCCTGTTTGCAGTGG

ACACGGGGCTGTACTTCTCTGTGCAGAGAGACCTTCAACAGTCCCTAAGGAACAAGAGGT

ATGGCAAAGTCACATGGAGCAAGAGCCCTGAGGACCCATACAAGCAATAAAAGCAGCATC

TCTGAACCTCTCTCTGGAGTCACAGCCCCATCGTCCCCAATGCTCATGGCTGGAGGCAGC

AGGAAAGTGACGCCAGAGTCCAGGCCCAGAGTCCTTCACCCAACTCTATTTTCTCAGATC

GGAAGAGCGGTTCAGCAGGA

**Adaptive Immune genes**

**MHC and related genes**

>Locus23_971_Transcript_3/5_MHC class I

CCCTCCAGGTAGGCCCTCAAGCGCTCCGCCCATCGGGTCTCCTCCCACTTGTGCCGGGTG

TTCTGAGCCGCCAGGTCGGCCGCGGTCCAGGAGCGCAGGTCCTCGTTCAGCGCGATGTAA

TCCGCGCCGTCGTAGGCGAACTGATCGTACCCGCGGAGGAGGCGCCCGTCCGGCCCCACG

TCGCAGCCGACCATCCGCTGTAAGCTGTGAGACCCTCGGGGTGACGCTCAGACCGGAGAG

TCGGGGCTATCCTGGGGAATAGGAGTCGTGACCTGGACCCGGGCCCCTCATCGCTCAGCG

GCCTCGCTCTGGTTGTAATAGCTGCGCAGGATGTTCAGGCTCCCTCGGGCAGTCCGTGCG

GTGTTTTCGTAGATCCGCGTGTTCCTGTCGCGATACCCCGGCTCCTCCTGCTCCACCTGC

TTCATCCACGGCGCCTGTGTCTCCACCCGCGGACTCGCAGCGTCGCTGTCGAACCGTTCG

AACTGCGTGTCGTCCACGTAGCCGACGACCAGAAAGCGAGGCTCCCCACTGTCGGGCCGG

GACATGGCCGTGTAGAAATACCTCAAGGAGTGGAAACCTGCCCAGGTCTCGGTCAGGGCT

AGCAATAGCAGGAGGAGGGTTCGGGGCGCCACGAGCCCCATGCTCGGGGTCTGGGGACAA

TCTGAGTCTGGCGATTTTATAATCTGGATCTGCGTTGACGCTGACTGGCTTCTCTAGAAA

GCGGACACGAAATGAGAGTGAGAACGGAGGCTGCGTCATGAGTATCCACGCGGAAAAGCC

TCACACAGGTTTCGCAAGAGA

>Locus31mer_4890_Transcript_1/2_ MHC class I

CTGACCGAGACCTGGGCAGGTTTCCACTCCTTGAGGTATTTCTACACGGCCATGTCCCGG

CCCGACAGTGGGGAGCCTCGCTTTCTGGTCGTCGGCTACGTGGACGACACGCAGTTCGAA

CGGTTCGACAGCGACGCTGCGAGTCCGCGGGTGGAGACACAGGCGCCGTGGATGAAGCAG

GTGGAGCAGGAGGAGCCGGGGTATCGGGACAGGAACACGCGGATCTACGAAAACACCGCA

CGGACTGCCCGAGGGAGCCTGAACATCCTGCGCAGCTATTACAACCAGAGCGAGGCCGGG

TCTCACACGTACCAGTGGCTTTCCGGA

>Locus23_2912_Transcript_1/1_ MHC class I

CCGAGGATGCAGGTCATGGGGCACCGAACCCTCCTCCTGCTGCTGCCGGGGGCCCTGGTC

CTGACCGAGACCTGGGCGGGTTTCCACTCTATGAGGTATTTCTTCACCTCCTGGTCCCGG

CCCGGGAGCGGGGAGCCCCGCTTCGTCGCCGTCGGCTACGTGGACGACACGCAGTTCTTG

CGGTTCGACAGCGACAATGCGAGTCCGAGGGCGGAGCCGCGGGCGCCGTGGATGGACCTG

GTGGAGCAGCAGGACCCGCAGTATCGGGACCGGCACACCCGGAACGCCCGGGACATAGCA

CAGGATTTCCGAGTGGGCCTGGACGACGTGCTCCGCTACTACAACCAGAGCGAGGCCGG

>Locus25_954_Transcript_1/8_ MHC class I

GGAGCCACTCCACGCACGTGCCCTCCAGGTAGGCCCTGTCGCGCTCCGCGTCATCGGGTC

TCCTCCCACTTGTGCCGGGTGTTCTGAGCCGCCAGGTCGGCCGCGGTCCAGGAGCGCAGG

TCCTCGTTCAGCGCGATGTAATCCGCGCCGTCGTAGGCGAACTGATAGTACCCGCGGAGG

AGGCGCCCGTCCGGCCCCACGTCGCAGCCGTAGCATCCACTGTATGGTGTGAGACCCGGC

CTCGCTCTGGTTGTAATAGCTGCGCAGGATGTTCAGGCTCCCTCGGGCAGTCCGTGCGGT

GTTTTCGTAGATCCGCGTGTTCCTGTCGCGATACCCCGGCTCCTCCTGCTCCACCTGCTT

CATCCACGGCGCCTGTGTCTCCACCCGCGGACTCGCAGCGTCGCTGTCGAACCGCACGAA

CTGCGTGTCGTCCACGTAGCCGACGACCAGAAAGCGAGGCTCCCCACTGTCGGGCCGGGA

CATGG

>Locus25_954_Transcript_6/8_ MHC class I

GGAGCCACTCCACGCACGTGCCCTCCAGGTAGGCCCTGTCGCGCTCCGCGTCATCGGGTC

TCCTCCCACTTGTGCCGGGTGTTCTGAGCCGCCAGGTCGGCCGCGGTCCAGGAGCGCAGG

TCCTCGTTCAGCGCGATGTAATCCGCGCCGTCGTAGGCGAACTGATAGTACCCGCGGAGG

AGGCGCCCGTCCGGCCCCACGTCGCAGCCGTAGCATCCACTGTATGGTGTGAGACCCGGC

CTCGCTCTGGTTGTAGTAGCGGAGCGCAGTCTGTAGGTTCACTCGGTAAGTCTGTGCGGC

GTCCCGGGCGTTCCGGGTGTTCCGCTCCCAGTACTGCGGGTCCTGCTGCTCCACCAGGTC

CATCCACGGCGCCCGCGGCTCCGCCCTCGGACTCGCGTTGTCGCTGTCGAACCGCGAGAA

CTGCGTGTCGTCCACGTAGCTGACAGAAATGAAGCGGGTCTCCCCGCTCCCGGGCTGGGA

CCAGGCGGTGTAGTAATACCTCAGGGAGTGGAA

>Locus25_954_Transcript_8/8_ MHC class I

GGAGCCACTCCACGCACGTGCCCTCCAGGTAGGCCCTGTCGCGCTCCGCGTCATCGGGTC

TCCTCCCACTTGTGCCGGGTGTTCTGAGCCGCCAGGTCGGCCGCGGTCCAGGAGCGCAGG

TCCTCGTTCAGCGCGATGTAATCCGCGCCGTCGTAGGCGAACTGATAGTACCCGCGGAGG

AGGCGCCCGTCCGGCCCCACGTCGCAGCCGTAGCATCCACTGTATGGTGTGAGACCCGGC

CTCGCTCTGGTTGTAGTAGCGGAGCGCAGTCTGTAGGTTCACTCGGTAAGTCTGTGCGGC

GTCCCGGGCGTTCCGGGTGTTCCGCTCCCAGTACTGCGGGTCCTGCTGCTCCACCAGGTC

CATCCACGGCGCCCGCGGCTCCGCCCTCGGACTCGCGTTGTCGCTGTCGAACCGCACGAA

CTGCGTGTCGTCCACGTAGCCGACGACCAGAAAGCGAGGCTCCCCACTGTCGGGCCGGGA

CATGG

>Locus27_2413_Transcript_4/9_ MHC class I

CTGAGGTATTTCTTCACGGCCATGTCCCGGCCCGACCGCGGGGAGAGCCTCGCTTTCTGG

TCGTCGGCTACGTGGACGACACGCAGTTCGAACGGTTCGACAGCGACGCTGCGACTCCGC

GGGTGGAGACACAGGCGCCGTGGATGAAGCAGGTGGAGCAGGAGGAGCCGGGGTATCGGG

ACAGGAACACGCGGATCTACGAAAACACCGCACGGACTGCCCGAGGGAGCCTGAACATCC

TGCGCAGCTATTACAACCAGAGCGAGGCCGGGTCTCACACGTACCAGTGGCTTTCCGGAT

GCGACGTGGGGCCGGACGGGC

>Locus25_12410_Transcript_1/1_MR1

GGACGTTCAAGATGGAACTGAAGCACCTGCAGAGTCACTACAATCACTCAGGGCTTCACA

CTTACCAGAGGATGATTGGCTGTGAGTTGCGGGAGGATGGAAACACCACGGGATTTCTGC

AATATGCATATGACGGACAGGATTTCATTATCTTCAACAAAGACACACTCTCCTGGATAG

CTGTAGATAATGCGGCTCACATCACCAAGAGGGCATGGGAGGCCAATCGGCATGAGTTAC

AATATCAGAAGAATTGGCTGGAAGAAGAATGTATCGCCTGGTTAAAGTGACTCCTGGAGT

ATGGGAAAGATACCCTACAAAGGACAGAAGCTCCACTGGTCAGAATAAATCGCAAAGAAA

CCTTTCCAGGGATTACAACTCTTTTCTGCAGAACTCATGGCTTTTATCCGCCAGAAA

>Locus25_854_Transcript_1/1_MHC class IIB

CTTCCACCAAAGTTTATTCCTCAGAGATGATCACCAGCAACTCACAGCCCTCTGTGGAGG

CCCCAGGAGGACCTTTAACTTCTTTCCCCAAATTATGTTCACCCCAGAAACATAGCCTCA

GACAATAACTTGTTTACAGAATATTCTCAAGAATGAGATCTCCCAAGTTACTAAGGTTTC

CATATGGGGTTACTGGGAAATTCAAAGAACTGGATGGAGAAACCATAGAATCCAAAAAAA

TGTGGAGGGTTTGAGGATGTTGGAGGAGCACTGCAGCATCACTAAGAGTTTGAATCTCCT

TCTCACTTGGAGTGGAAGTTGTAGAACCCTGCCTCTAGGAAATGTGATGACCTTCTGGAT

AATTGGACCCAGGGAGGGAAGTGTAACCAGAGGAGCCAGCTCTCCGCCAACTGAGCAAAC

CAAGAGAGAAGATGATGAGGCCCAGGCCCAGAGTCACTGCAGACACAGAAACCTTCAGTG

TCTGTACCGGGGACAGCCCAGGTGTCCAGTCCTGAAGGATGGGGTCGGGAGCCCCAATAT

GTTCCACCACACAGGTGTAGACATCCCCGAAAGAGGGGGTTGTGGCTAAATAAGAGATGG

TCTGGTATGTCCAGTCTCCATTGGGCTGGGCAGTCTTATGGGCGCTGCCATGAGGGAGGA

CAAGCTTCCCGTTCTTCCTCCACGTGATGGTCACATCCATTGGATAGAAGCCCCACACAT

AGCAGGCCAGCATCACGGGCTCCCTTGTGTTAAAAGGAGTGGTTTTGGCTACTTGCACAG

ATGGTGGCCGTGTCCTGTGGGTCAGTGATCCCCAGAAGGGCTTTGTGTGTGTGGCACAGT

TCTGGAGCCCCTCAGTCAAGCGCTGGAGCAGGTTTTCCTGCTGATTGAGGTAACTCGAGA

GGTAAGTGGCCAAGACATTCAGTGCCCCAAATTCACAAGGGGCCATTCTGTGCTCCTTTG

GATCCCAGCAGGTCAGCAAATCTTTGTTGAAGGATATACAATATGTGAATTCCTGTGGAG

TCCCATCATCATCCAACAGACAGGTGCTTTCCACATGGGCCACAAAGCCATCTGCTCCGG

TGCAGCCCAGGCCAAGGCCCAGCAGCAGCTGCAGGAGTATGGTCATGGTCTGCTGTGGGA

AGACACAGGCAATTCGGTCCCCTGGACCAGCTCCTCCAGGGGCCTTGAAACCTTGCCTGC

TCCAGAAACCCCAGTCTGGGTAGATGATCTTCAGACCTTGAGCGGAATATGGTATTACCC

GGGCCCCTTGATCCCTATTAAATGAGTGACTTAGAGCTACAGATCGGAAGAGCGGTT

>Locus27_1995_Transcript_2/5_MHC class IIB

CGGCCCCTTCCGCGCTCCCTTCCGTGGGCTTCGGTCAGAGCCGGGAGTTCACGGCTTTGC

CCGAGGGGCGACCGCGTCCCAAATTCTTGATAAAACGTGGGACTGACTCATAGGAGTGGA

GACAAAAAAAAAAAAAGGCTCACTCTGCTCGTCTCTGAGCTTGGGGCCACAGAGGACATT

CTGGTTTGTCTTCCAGGGTCTATCTTTATAATCCGGATGACACAGAAGCACCAACACTGA

GCTTATTATGCAGGGTCATGAAGCGTCCTGGGGTGGACGCGAGAAAACCACTGACAAGTT

CTGATTTCTAACTGCCACCCGAAACACTGGTGACCACACAGCTGTGTTACCGGCACCGGA

GTCCTCTGCTCGCGCACAGTGCTCTGCGAAAGTTAGCCGAGGCGATGCGACGAAAATGAA

GGGCTGAAACGTCTAGGAAACACGAAACACCTCCATCCCTCTCTTCCCTCCGAGTGCTCG

CTGAATCCTGTCCTCTCTGCTTTCCAGCATGGTGTGTCTGTTGTTCCCTGGAGGCTCCTG

GATGGCAGCTCTGACAGTGATACTGATGGTGCTGAGCCCTCCATTGGCTTGGGCCAGGGA

CACCTCACCACATTTCTGGTTTATGGGGAAGGCCGAGTGTCATTTCTTCAACGGGACGGA

GCGGGTGCAGTTCCTGGAAAGATATTTCTATAATGGGGAGGAGTACGCGCGCTTCGACAG

CGACGTGGGGGTGTACCGCGCGGTGACCGAGCTCGGGCGGCCGAGCGCGGAGCACTGGAA

CTCGCAGGCGGACATCTTGGAGCAGAGGCGGGCCCTGGTGGACACGGTGTGCAGACACAA

CTACCAGGCGGAGACCCAAACGTCCTTGAAGCGGCGAGTGGAACCCACAGTGACCGTCTC

CCCATCCAGGACAGAGGCTCTAAACCACCACAACCTGCTGGCTTGCTTGGTGACAGATTT

CTATCCAGGCCAGATCAAAGTTCGGTGGTTCCGGAACGACCAGGAGGAGACAGCTGGCAT

CGTGTCCACCCCCCTTATAAGGAATGGAGATTGGAACTTTCAGATCCTTGTGATGCTTGA

AATGATTCCTCAGCGTGGAGATGTGTATACCTGCCACGTGGAGCACCCCAGCCTCCAGAG

CCCTATCAAAGTGGAGTGGCGAGCACAGTCTGAATCTGCCCAGAGCAAGATGCTGAGTGG

CATCGGGGGCTGCATGCTGGGGCTGATCTTCCTCGGGCTGGGCCTTTTCATCCGTCACAG

GAGCCAGAAAGGTCTTCGTGGGTCTCCACCAGCAGGTATATTTTTCTGCTCTGATTCAAT

GGGGAATATGGGGACAGGTAAAAGAGAGAGGGGTATGAGGTAGATATACCTGGGCATGAT

GGTCTTAGTTTATGGCCTATTCCCTGCTATGGGGATCAAGAGTTATGGGAGGTTTGCCCA

GTTTCTGTAGGAAGATCCTGGGGTTGTTTCACTGAACGAGGGTGTAACTTTGGTGGCATC

TTTCTTTGGCGTTAGACATCGGGATTATGCCCACTTTGTACCACATGTTGTTGGTTACTG

TGGGCATTTATAAGTGAGTGAAAGAGGCTACTAGTAGTGGAAAGAAGGTG

>Locus29_225_Transcript_6/9_MHC class IIB

AGAGTGTCTTTTTTTTTCTTTTTTTTCTTCATTTTAGGAGATGGTTTTCTACGATGCTTG

TAATAGGTATCAGGACTAGGATAAGAAGGAAATATAGGATAGATGCTAGTTGGCCGATAA

TGATAAATGGGTGTTCGACGGGTTGTCCGCCGATTCAGGTTAGTGTTAGTAGGTCGGCCA

CTAAGAGTCAGAATATGCATTGGCTGAGTGGTCGGAATATTATGCTTCGTTGTTTTGATG

TGTGTAGGAGTGGCATTAGAATTAGGATTAGGATTGATAGGACTAGAGCTAGAACTCCTC

CAAGTTTGTTGGGGATAGATCGTAAAATGGCGTACGCGAACAAGAAATATCATTCTGGCT

TAATGTGTGGAGGGGTATTTAATGGGTTGGCCGGGATATAGTTGTCTGGATCTCCCAGGA

GATCGGGAGAAAATAGGACTAATATTAAGAGTGCTAGGACTATGGCTAATGCGCCTAGTA

TGTCTTTGATTGTGTAATATGGGTGAAATGGAATTATGTCTGAGTCTGATGGGATTCCTG

TTGGTGCTAGGCACCCCAGTGGCTGAAGGCAGAGACTCTCCACAGGATTTCGTGTACCAG

TTTAAGGGCTACTGCTACTTCACCAACGGGACGGAGCGGGTGCGGATGGTGACCAGATAC

GTCTATAACCGGGAGGAGTACGCGCGCTTCGACAGCGACGTGGGGGTGTACCGCGCGGTG

ACCGAGCTCGGGCGGCCGAGCGCGGAGCACTGGAACTCGCAGGCGGACATCTTGGAGCAG

ACGCGGGCCGAGGTGGACACGGTGTGCAGACACAACTACGGGGTTGTTGAGAGCTTCACG

GTGCAGCGGCGAGTTGAGCCTACAGTGACTGTGTATCCTGCAAAGACCCAGCGCCTGCAG

CACCACAACCTCCTGGTCTGCTCTGTGAATGGTTTCTATCCAGACGACATTGAAGTCATA

TGGTTGCGGAATGGCCAGCAAGAGGAGGCTGGGGTCATCTCCACAGGCCTGATTCATAAT

GGAGACTGGACCTTCCAGACCCTGGTGATGCTGGAAACTGTTCCTCAGAGTGGAGAAGTC

TACACCTGTCAAGTGCAGCACCCAAGTCGGACAAGCCCTATCTCAATGGAATGGAGAGCA

CAGTCTGAATCTGCACAGAGCAAGATGCTGAGTGGAATCGGTGGCTTTGTCCTGGGTCTG

CTCTTCCTTGGGGTGGGGCTGTTCATCCACTTCAGGAATCAGAAAGGACACTCTGGACTT

CAGCCAACAGGACTCCTGAGCTGAAGTGAAAATGGTGTCACTCAAAGAAGAAAGAACCTT

CTGTCCCAGATTCTTTGCAGCGTGAAAAGATTTCCTGCTTGGCTCTTATTCCACAAAGGG

GGTTCTTCCTCAGGATCTGGTTTGCTCCTGGTTCAGTGACCTTACAGAAAATATCCTCCC

TGATGGCTTCCTCAGCCTCTGCTCTTGACCTGGAAGTTCCCAATATTGATTGCAGTATCT

TGTCTTTATTCTTTCCCTGTCCCCTTTGTATTCAACCCTCAGTGCTGCCTGTGTATCTGA

ACTGGCCTTTTGCCCACATTTCTTTATGAAGATTTTCTCAAATAAACGTGAAGTAAAAAT

CCTCTATTTCATACACCTTCAAGGACAAGAAGTAGAAAAAAAAGAAGAGAGATTACATGA

AAATTCAACCATGGTTTTCATTTATATTCCTGAATTTTGGTCACATGTGGATCTGGAAGG

TTTCCTTGTTTTCTTCTGTGCCTTTTCATAACTTTCTAGTATTCTGTAAATAGGCATGTT

TTCCTGAGCTAGTAGAAAGCCCTTAATGAACCCATGTCATGGTGGTCTCCTACATCTTCG

TGTTCCCAAAGACAAACACACAGGTCCCTTCACACAACAGGGTACAAAAATGGGACACAA

ATATAAGAAATGCAGGTAACTTGTAAGGATGTTATGCCTTCAAAATTTTTATAAAGATGT

TCTTTT

>Locus23_36201_Transcript_1/1_MHC class IIB

CGCTCTTCCGATCTAGGGGGCGAAGCTTCTGTTAGGACTTTAGTATGTCAGTGGTGGCAG

AACAGATCTTGAGACCTTATCACCAGACAGCTGAGTCTCTGCATATCCTTTCTGAGCCCT

AAGGTGGACGGCGATCCCCACCAGAAGGAACATTAGTCCAAGCAGGAAGGCTATCACTCC

ACTTAGCATCTTTCTCCAAGAATATTCAGACTGAGCTCTCCATTCCACTGAAACAGGGCT

CAGCAGGCTGGCATGATCAACAAGGCAGGTATAGACATCTCCAAATTCAGGAGTCATTTC

CAGCATCACCGTTATCTGAAAGGTCCAGTCTCCATTCCTTATAAGGGGGG

>Locus31mer_8595_Transcript_1/1_MHC class IIA

TCCTGCTGAACCGCTCTTCCGATCTTGTTATAGCACCAGAAGGCAATGGTCTTCAGTGGG

GGGCTGGTCCTGGGACTCCACACCTTGATGGCGTTCCTGAGCCCCCAGGAAGCTGGGGCC

ATCAAGGCTGACCACATGGGCTCCTATGGACCAGCCTTCTACCAGTCCTATGATGCCTCA

GGTCAGTTCACGCATGAGTTTGATGGGGAACAGCTGTTCTCTGTGGAGCTGAAGAAGAGG

GAGGCTGTGTGGCGTCTGCCTGAGTTTGGCGACTTGACTCACTTTGACCCGCAGAATGGG

CTGGCCAGCATTGCGATGATCAAAGCCCATCTGGACATCTTGGTGGAACGCTCCAACCAC

ACCAGAGCCACCAACGTGCCTCCAAGGGTGACTGTACTCCCCAAGTCTCGAGTGGAGCTG

GGCCAGCCCAACGTCCTCATCTGCATCGTGGACAACATCTTCCCCCCTGTGATCAATATC

ACCTGGCTACGCAATGGTCAAACAATCACCGAGGGGGTGGCTCAGACCAGCTTCTATTCC

CAGCCTGATCATTTGTTCCGCAAGTTCCACTACCTGACCTTCGTGCCCTCAGCAGATGAC

TTCTATGACTGCCAGGTGGAGCACTGGGGCCTG

>Locus31mer_645_Transcript_1/1_MHC class IIA

AGGAGTTGGGATCTCAGGCTCCCAGTGTTTCAAAAGTGGCTTGTCCAGGCCCCAGTGCTC

CACTTTGCAGTCATAAATATCATCAGCAGAAGGGAGGAAGGTGAGGTAACTGATCTTGAA

GAAGGAATGATCATTCTTGGAGATGAAGCTGGTCTCAAAAACACCTTCTTTGACTGAGTG

CCCATTGCTTAACCATGTGATGTTGATCACAGGAGGAAATATGTTGTCCACAAAACAGAT

AAGGGTGTTGGGCTCACCGAGCATCATGGGAGACTTGGGAAACACAGTCACCTCAGGAAC

CTCATTGGTAGCAGGGGTAGAGTTGGAGCGTTTAATTAAATCGTCCAAGTTGTATTTTGC

TGTAGCTATTTCTCTCAGTCCACCCTGTGGGTCAAAACCTGCAAATGTGCTAAACTCAGG

CAGCCGCCAGACAGTCTCCTTCTTCTCCAGGTCCACATAGAACAACTCATCTCCATCAAA

TTCGTGGGTATACTGGCCAGAGGGACCATAAGCATGGTAGATGTTTATGCCATAGGAACC

AACGTGGTCAGCTATGATGTCTTCACCTCCACAGGGGCTCATCAGGGTGGTCAGGGTGAG

GGCCCCCAGAATCAGAGCTCCAAGATCGGAAGAGCGGTTCAGCAGG

>Locus27_784_Transcript_4/7_MHC class IIA

GCCCCAGTGCTCCACCTTGCAGTCATAAATATCATCAGCAGAAGGGAGAAAAGTGAGGTA

ACTGATCTTAAAGAAGGAATGATCATTCTTGGAGAGGAAGCTGGTCTCAGAAACACCTTC

TGTGACTGAGTGCCCATTGTTCAACCATGTGATGTTGATCACAGGAGGAAATATGTTGTC

CACAAAACAGATAAGGGTGTTGGGCTCACCGAGCATCATGGGAGACTTGGGAAACACAGT

CACCTCAGGAATTTTTATGGGAGCAGCGGTAGAGTTGGAGCGTTTAATCATGATGTCCAA

GTTGTGTTTCGATATAGCTATATTTCTCAGTGCACCCTGCGGGTCAAAACTTCTAAATTC

ACCAAGCTCAGGCAGCCGCCAGACGGTCTCCTGCTTCTCCAGGTTCACGTAGAACTCCTC

ATCTCCATCAAATTCATGGGTGTACTGGCCAGAGGGACCGTAAGACTGGTAGACATTTAT

GCCGTAGGAGGCAACATGGTCAGCCACAATGTCTTCACTTCCACAGGGGCTCATCAGGGT

GATCAGGGTGAGGGCCGAAGGGAAAAGCAAGAGCGTGTGACAGCATTCTGGACTTCTCAC

GGCACGCAGGGCGCCCCGTCCTCCCCCCGACTCCTGAGACAGCCCCCCTCACGTCCGAAC

CTGCATGCGCTCCTCCCGCCCGGTCCCCTGGACTTCGGTGACGAGACTCTCCATCCACGG

CAGCCTCGGGTCCCTTCCAGGCACACAGTAGGACTCGGGCAGTGAGATGGGGTTGTGGGG

GTGAGCAGGGGGGATCACAGATTTGTCGGGTGGGATGCTGGTGCTGTGGCCTCGTTAAAA

AATAAACCCCAACCCGTTAAAGGCGGGTCTGTGAGTGTTCCCGGCTGGAGCGATGTCATG

>Locus31_2258_Transcript_1/1_MHC class IIA

ACTGCCGTCACAGCCGTCTTCGGCTTCCCACAGGATCACCCCGACCTTCTCCATGAGCTC

CTTCAGCTGGGCCTCCCGCTCGTCCCCCTCCGCCCTGTTGTCGAAGCCACAGTGGCGCCG

CTCGCACACCACGTCCAGCATGACGAGGTCCTGGTTGTCAGTCTCGCGCACATACTCCTC

CAGAGAGCCGCCGGCCAGGTCCTCCTTCCGAGTGAACACCAGGACGGTGGAGGCCAGGAC

GCTCTCTCCGAAGACGTCCTGGAGGCGCCTGACCACCTCCCGGTCCTCTTTCGTGAACCG

CCCCAGCTGCGTCACCAGGAGCACCACGTGCAGCCCTGGGGAGGAGAAGCCAGTGGCTTC

ACGCAAGTCACGGGCTGCCACGTCCAGAGACACCGCAGGGGACAAGACATCGGGGGTGTC

GATCACCTCGAGCTCCTTCCCGTCCCATCCGCAGCTCTCTCGCTGGACGGCCTTGGTCAC

AGCCCTGGCACTGACTTTAGACTCAAAGGCTTTCCTGCCGAGGATGCTGTTTCCTGTGGC

GCTCTTCCCACTTCCCGATTTCCCCACCAGGATGAGCTTCAGTGTCTTCGGGGTCTGTTC

TGCTCCACACTTAGGCTCTACTGATTCCCATCAAACCACCCAAGAAGAAAATGGCCATAA

TTGGAATCCCGATGTTTGGATTTTTCATCACAGTTCTCCTGATGAAACTTCAGGAATCAT

GGGCTGTCAAAGAGAATCATGTGATCATCCAGGCTGAGTACTATCTGACCCCTGACGCAT

CAGCTGAGTTTATGTTTGACTTTGATGGTGATGAGATTTTCCATGTGGATATGAAAAAGA

CGGAGACGGTCTGGCGGCTTAAAGAATTTGGTGATTTTGCCAGCTTTGAAGCTCAGGGTG

CACTGGCCAATATAGCTGTGGACAAAGCCAACCTGGACATCATGATAAAGCGTTCCAACC

ATACCCCGAACACCAATGTACCTCCAGAGGTGACTGTGCTCCCAAACAACCCCGTGGAAC

TGGGAGAGCCCAACGTCCTCATCTGTTTCATCGACAAGTTCTCCCCACCAGTGGTCAATG

TCACGTGGCTTCAAAATGGAAAACCTGTCACCACTGGAGTGTCAGAAACAGTCTTCCTGC

CCAGGGAAGACCACCTTTTCCGCAAGTTCCACTATCTCCCCTTTTTGCCCTCAACTGAAG

ATGTCTATGACTGCAAGGTGGAGCATTGGGGTTTGGATGAGCCTCTTCTCAAGCACTGGG

AGTTTGAAGCACGAACTCCACTTCCAGAGACAAAAGAGAATGTGGTGTGTGCCCTGGGCC

TGGTTGTGGGTCTGGTGGGCATCATTGTTGGGACCATCTTCATCATCAAGGGAGTGCGCA

AAGGCAATGCTGTTGAACGCCGAGGACCTCTGTGAGGCACTTGCAGGTAATGGACTTTCC

TAAGAGAAGAATGAAAGATCAATGAAGAGATTTCTGCTTTAATATCTTTACAAACCTGGC

AATATTTTAGTTGTTTATCTCAGTGAAGACAACCATTCTTCAGCACTTTCCAGCCCTTTA

GTCAGTCTACGAATGATGATGCCTTCTAGATCTCTCCATGCTCTAACATCTAGTTAGGCT

TCCTCGTCTGTTACTCCTTCCTGTATCTGTTTTCCCTCCATTTCCCACTATCTTTTGTTG

GCATGCAATGGCTCTAAAATAGGCCCCATAAGATCCTTTTTCACTATGGAAACTTTAAAA

ATGTTCTATGGGAGCATCTCCTTTGTACTTATTGCTTGAGGTTTCTTCAAACTGTGATTG

TGATATTTCCAACTA

>Locus25_15339_Transcript_1/2_MHC class IIA

GCCTGGGTCAGCTGGTTGGTAGCTCCTAGACCACTGTGTGGATAAAGCTATGGATTATGA

ACTGAGCCAGGGAGCTGCGCTGCTATGGCTGCTACCCCTTCTGTGGCTGCTGCCTCACTC

CTGGACCGCCCCCCAAGCTCCTACTCCAAGATGGCAGGATGATCTGCAAAACCACACATT

CCTGCACACGATGTACTGTCAGAACGCGAGTCCCAGTGTGGCACTCTCTGAGAGCTACGA

CGAGGACCAGCTTTTCTCCTTCGACTTTTCCCAGAACATTCGAGTGCCTCGCCTGCCTGA

ATTTGCTGACTGGGCTCAGAAGTCTGAAGATATTTCCACCATTTTCTTTGACAAAGGATT

CTGCCACGCCATGATCCATGAAATCGGCCCACGACTTGAAGGGCAAATCCCAGTGTCTAG

AGGGATTCCTGTTGCTGAGGTGTTCACTCTGAAGCCACTGGAGTTTGGCAAGCCCAACAC

ACTGGTCTGTTTTGTCAGTAATCTCTTCCCGCCTGCATTGACAGTGAACTGGCAGCATCA

TTCAGCCCCTGTGGAAGGAGTCGGACCCACTTATGTCTCAGCCATTAATGGACTCAGCTT

TCAGGCCTTTTCTTACTTAAACTTCACACCGGCACCCTCTGACATTTTCTCCTGCATCGT

GACTCATGAAATAAACAGCCACACAGCAATTGCCTATTGGGTGCCCCAGAACGCACTGCC

CTCAGATCTTCTGGAGAATGTGCTGTGCGGCGTGGCCTTTGGCCTGGGTGTGCTGGGCAT

CATAGTTGGCTTGGTCCTCATTATCTACTTCCGAAAGCCTTGCTCAGGTGACTGAGTCTT

CCTGGCCAGAGTCTGATGCCAGCAACTTTGGCCATCCTAGCAGAGGGTGCTCAGGTTTCT

CATCTCCTGCTCAAGATTGCTTCTTCTCAGAGCAGAAGGCCTTGGGACTCCGTGTGTGTG

TGTATGTGTGTGTGT

>Locus21_26361_Transcript_2/2_ULBP

CTCACGGAGTCTAAAAATGAATAGCAGTTATCCAAGGCCGCCAATCAAAATTCACAGTCG

CTGAGACAGATACCCTGTACTGGGCTTCCTGAGTGCGTTGCTGGCCTGTTACTGCAAAGG

ACTTAGCCGGTGATTCCAAACATAATCTGGCAGGTAAGGGCCACAAGGAGAGTCCAAATG

GTGGATGTGACGGTCGTGGCCTTGGAATGGGCTGTGGCTGGGGCCGTGGTTGGTGGGTCT

GTTGTGTTCAACGCATTATCCTGGTGATAGCTCATATTCTTAGGCCAACTCTCACACATT

TCAGCTGAGTTTTTCTTCAGGAACTCATACACCTCTTTCTCATTGTTCAATGTGTTTAAC

TGCTCGCCTTCAGGAATCAACACTGTCCAGTTTCCGTTCTTTGAGTCAAA

>Locus19_5888_Transcript_2/2_FcRn

TCCAGAGGGAGGACGAAACGAACCCAGAGGGAGGACACGGGGCCCAGGACTCCTGGAGGC

TCAGAAATTCCAGGAGGTCTCACAGCATGAAAGGGACCTGACTACATTAGCTGGTAGCAG

CCAGGATGGAAGACGATCTGGCAGCCGCTGGGGATGCTTTTATGTCCTAAGAGTCAGCAT

CCTTGGGCAGGCCAGGCGTGGGGAGGAGGGCCCCTCCGTCGTCCGCACGGAGAGAGATCC

AAGGGGCTGGCATCCCGGTCTGCATCCTCCTCCACAGCAGAACTCCTCCTATGACCAGGG

CCACGAACAGTAAGACGCCGATGACGATTCCAACCACTGCCATGGAGGACCTGGCTGGTG

ATTCCAGTTCCACAGTGAGGGGTTGTGGCAGCCCCACGTGCTGCACCAGGCAGCGGTAGT

GGTGTTCGTCGCCACTTTTGACCGTTAGTGACGACCAGGCGTGGAAGGAGCCGTCGCTGT

TGGGGCCGAGGTGGCTCTCCCCGGAGCCAACCGCCAGCTCATTCCTTAGGAATCGCAGTT

GCAGCTCAGGAGGGTAGAAGGAGAAGGCACTGCAGGTGAGCACGGAAAAGCCGGGGCTGC

CTGGACGTGCCTTCAGGCGCATGGACGGTGGCTCCTTCCACTCCAGGTTGCCGCGGCCCC

TCTCCAGATGGCCCAGCAGGCGCTGGGGACAGGAGTACAGCAAAAACGTCTTCTCCTTGT

TGACCGCCTCCTCCTGATGCTTCCAATGCTCACTGATGGTCTGGGCCTCAGGCCAGTCTC

CATTCCAGGTGCCCGCCTTGGGGTCAAAGGTCATGAAGTCCTCGCCATTCAGGGCAAACT

TGGCCACCGGCACGGAGACGTTGTCAGGGCCCAACTCACAGCCCAGCAGGCCCTGTAGGG

TGTAGGAACCGCTTTTCTCTAAGACTGTGAAAGCTTCGAGAAAGAGCTTCTGCTTCTGCC

TCAGGTCTGTGGTCTCTTTCTCCCAATACCAGGACACCTGGTTCTCCCAGACCCAAGCCC

CGCACGGCTCGGCCTGCGCCCGCAGGTTATTGTAGCTCAGGTACTGCTGTGGACCCAGCC

AGCCTGACACCCAGAAGGCGGGGGTCCCCGGGGCCGGGGACGACACGCAGGTTAAGTGGT

ACAGGAGGGAGCGGTAGCTGTCTGCGCGCAGCGTCTGCGGCGGGAGCAGCAGCAGGATCG

GCAGCAGACAGAGCCCCCAGGGCTGAGGCCGGGGAATCCGCATCCTGAGAGGGCGACCTC

ATTTACCCCCAGATCTTCACTATCTCTTGCTACAAAGAAAGGTCTCCAGAT

>Locus31_165_Transcript_3/3_ beta-2-microglobulin

TGAATCTTTGGAGTACGCGGCTCGCCGTCTTGGCCCTGTTCGCGCTGCTTTTCCTCTCTG

GCCTAGACGGCGAGCCGCGTACTCCAAAGATTCAGGTTTACTCACGGCATCCAGCAGAGA

ATGGAAAGCCAAATTACCTGAACTGCTATGTCTATGGATTCCACCCACCTCAGATTGAAA

TTGATTTGCTGAAGAATGGACAGAAGATGAAAACGGAGCAGTCAGACTTGTCTTTCAGCA

AGGACTGGTCGTTCTATCTTCTGGTCCACACAGATTTCACTCCCAGTACAGTGGATGAGT

ACAGCTGCCGTGTGAATCACAGCAGTCTCGCTGCGCCCCATATGGTTAAGTGGGATCGAA

ACAACTAACCAGCATCATGGAGGTTTGAAGATGCCTC

>Locus19_11823_Transcript_1/1_HFE

TTCCGATCTCGGCTCCAGGCCTACTGGGCCCACAGAGGCCCGTTCTCTCCCCAGGGGCAC

ACTGTCTGCGCTACCTCTTCATGGGTGCCTCGAAGCCCGACCTCGGGCTGCCCCTGTTCA

AGGCCCTGGGCTACGTGGACGACCAGCTGTTTGTGTCCTACGATCACGAGAGTCGCCAGG

CTGAGCCCCGTGCTGCCTGGGTCAGGGGCAGGGTTTCCAGCCAGCTGTGGCTCCAGCTGA

GCCAGAGTCTGAAAGGCTGGGACCACATGTTCATCGTTGACTTCTGGACCATCATGGACA

ACTGCAACCAGAGCAGGGTCACGAATCTGGAGGTGGTGCCGGAGCCCCACATCCTGCAGG

TCGTCCTGGGCTGCGAGGTGCGCGAGGGCAACGGCACCAGGGGCTTCTGGGAGTACGGCC

TCGACGGGGAGGACCATCTCACGTTCCGCCCGGAGACGCTGGACTGGAGAGCAGCGGAGC

CCAGGGCCCAGGCCACCAAGCTGGAGTGGGAAGAGATCAGGATCCGGGCCAAGCAGAACA

GGGCCTACCTGCAGCGGGACTGCCCCGAGCAGCTACAGCAGCTGCTGGAGCTGGGGACAG

GGGTCCTGGACCAGC

>Locus25_352_Transcript_3/3_MHC class II invariant chain

CCAGCTTTGTGGCTTTCACTTCCACTTCTACCAGGTGAGAGGAGTGGCCTCCTGTGGACG

AATCAGATTCCTTCTCAACCCCAACTTCAAGAGGTGAGCCAGCAGTTCAGGGTCTCAGAT

ACGAAGAGAGCAGTGGCAGCAGCAGCAGCAGCAGCAGCAGTGGTAGCAGGAGGTGTGGGG

AGAACCAGAGGCCAGAACGATGGAAGACCAGCGAGACCTCATCTCCAACCACGAGCAACT

GCCCATGCTGGGCCAGCGGCCCGGAGCCCCGGAGAGAAACTGCAGCCGAGGAGCCCTGTA

CACAGGCTTTTCTGTCCTGGTAGCTCTGCTCCTGGCTGGCCAGGCCACCACCGCCTACTT

CCTGTATCAGCAGCAGGGCCGGCTGGACAAGCTGACGGTCACTTCACAGAACTTGCAACT

GGAGAACCTGCGCATGAAGCTTCCCAAGTCTGCCAAACCTGTGAGCAAGATGCGGGTGGC

CACCCCCATGCTGATGCAGGCTCTGCCCATGGACGGCGTGCTTCAGGGGCCCATGCAGAA

TGCCACTAAGTATGGCAACTCGACACTGGACCATGTGATGCACCTGCTCCTGAAGTCTGA

CCCCCTGAAGGTGTACCCACAACTGAAGGGGAGCTTCCCAGAGAACCTGAAACGCCTTAG

GAACACCATGGAGGGCCTGGACTGGAAGGCCTTTGAGAACTGGATGCATCAGTGGCTCTT

GTTTGAAATGAGCAAGAACTCACTGGAGGAGAAGCCCAAGCCCACTCAAGTTCCAACAAA

AGAGCCAGTGGACATGGAGGACCTGTCGTCCGGGCTGGGTGTGACCAAGCAAGATCTGAG

CCAAGCCATCAT

>Locus23_1612_Transcript_1/2_Tapasin

CACCTAACCAGAGCGGGGATTTCTATGGCCCCTCCTGCTGGCTTTTTGAAGAAAGCGAAA

GTGAAAGAGGGAAGAAGACTGAGGAGATCGCGGCGCCATGAAGCACCTGTCCCTGCTCCT

CGCTCTGGCTTTGGGCTTGGTGACCGCCGTCTCGGCGGGACCCACGGTGATAGAATGCTG

GTTCGTGGAGGATGCAGACGGGGGCCGCCTGGCCAAGAAACCCGCTGCATTGCTGCTGCG

CCAGGGCCCAGGGAGAATACCTCCCCGGCCGGACCTCGACCCCAAGCGCTACCTCAGAGT

ACATGACCCCACAGGCACCCTGCAGGCTGCCTTCAGGCGGTACCCCCGGAGCGCCCCCAC

GCCACACTGCGAGATGAGCCATTACATCCCACTCCCCGCCTCTGTGAATTGGGTCAAAGG

CCTGACTCCGGAGCAGAGCTGCCCGCGGGCCTTGGACGGGACTTGGTTCATGGTCAGCAT

GTCCAGCCCGGTCCTCAGCCTCTCCAGCCTCCTACAATCACAGACCGAGCCTCAGCCAGA

TCCTGGACTCATCACCATGGCAACAGCTGTGCTGACTGTCATCACCCACAACCCCACCAC

TAGAATCCAGCTGGGACAAGATGCTCTGCTGGACTTGAGCTTTGCTTACATGCCCTCCAC

CCCAGAGGCCACTACACCTCTGGCCCCAGGTCCCCCTCCCTTTGGGCTGGAGTGGCGACA

CCAGCACCTAGGGGAGGGGCACCTGCTCCTGGCTGCAACTCCTGGGCTGCGTGGGCAAAT

GCCAGCTGCCCGAGATGGGGCAGTGGCATTTGCCGCTTGGGATGATGATGAGTCATGGGG

TCCATGGACTGGAAATGGGACCCTCTGGCTGCCTGCAGTGAAGCCTTCCCAGGAGGGTGC

CTATCAGGCCACCATATACCTGCCATATCTGCAAGGACAGGTCACCCTGGAGCTTGCTGT

GCAGAAATCCCCCAAAGTGTCCCTGATGCCATCACCTCTTGTATGGGCTGCCCCTGGGGA

GGCACCCCCTGAGCTAGTTTGCCTTGTGTCCCACTTCTACCCCTCTGAGGGCCTGGAGGT

GGAGTGGGAGCTCCGAGGTGGTCCAGAGGGGAGCTTTCAGAAGGCCAAGGGGCAGAGTTG

GCTCTCAGCCCTGCACCACCACTTGGATGGCTCTGTCAGCCTCTCTGGGCACCTGCAGCC

ATCCCCGGTCACCACTGCACAGCACGGGGCGCGCTATGCCTGTCGTGTCCACCACCCCAG

CCTACCCGCATTGGGGCGCAGTGCTGAAGTCACCCTGGAGGTGGCAGGTCTCTCTGGCCC

CTCCCTGGAGGATGGTATAGGCCTCTTCCTGTCTGCTTTTCTGCTCTTGGGGCTCTTCAA

GGCACTGGGCTGGGCAGCTGCCTACCTGTCCACTTCCAAGGAATCAAAGAAGAAAGCACA

GTGAGGGCACTCAAGACCACCCTGGGGAAGCTACCATCATCTCTGACCTGAACTACTGTA

GTAGCTCCTCCAAACAGTATGCCATCACCTGCTCCTGCTCCTGCTCCTTCCAATCTCTCT

CCACGGTGGGAATGCTTTTTTAAAAAAGACACAAATCTATAGCATTCACCTTCTTAGAGC

TCTAAGGCTGTGGTTCTCAAAATTTTTGGTCTCAGGACATCATAAAAACGAAGGACTCTC

AAGAGCTTTTGTTTATGTGGGTTATAAACTAACTATTTATCATACTAGAACTTAAAACTG

AGAAAAGTTTAAAACAGAAGGATACACAGGCACACATTCCATTGGCTGTGAAGGAGATAA

TGTCAATACACATCATGTAGCTCTGGGAAATTTCACTGTACATTTTTTTAGAGAATGAAA

GTGAAAAAGGCAAATAACATTTTAGTGTTATGAAAATAGTCTTAACTTCGTATGCGCAGT

GAATTAGTCTCAGAGATCTCTGGGGCAGGGACCACACCAAGAGAACGGGTGCTCTAGAGG

CCAAATCCATTCTTCTTGCCCTTGGCACATGAATTTACGGCGCTGGAGCCACTGCTTACC

TCTTCAACCCAATCTGGTGCTCTCCAGCACTCTCAATACTCATCACACCTACAGCTCTTC

CTTTCCTCCAATGGCCTCTCACCTTCCCTAGGTCCCAGAGTTCTGTTCTCTCTGCCTGGT

CCACCCTCGCTCTCCCTGGGCTCCAGACCAGATCAGCCAAGTCATTCCCCTCAGACCGCC

GCGTGTCCCTCTTGTGCGGTTCACCACGGTTGTACTTAAGTGATTGTGCGAGTTGTTAAA

GCCCGTTTCCCGGACTCTCAGCTCCTCGCAGTTGCAAAAACAAGTCTGTATGTCCACTGC

TTTATCTCCCGCATCGGCAGTGGGCCTGGCGCCCACTGGAGGGCGCTCGGTATAAGAAGT

TATAGTCCTTTTTTTCATCCTAAGGATTCAAAAACAGGTATAGGGTCAAGTAACTACTGG

ACGAAGGAAGCGGCGACTTAAGGACCTCCGAGGGGCAGGACCGTGGGCCAGACCGACGCG

GGGAGCGAGTCTACTCGGTGGGAATAAAGTTGTGTTCCAGTGAGCAAAAAAAAAAAAAAA

AA

>Locus23_128_Transcript_1/2_calnexin

GCGGAGGGCGTGCCCGGGCGTCTGCCTCCGCCCCCTCCACCGTGTCTCGGCACATGCTGC

ACACAAGCGCCCCTGTGCGTGTTGAGGTGCAAGGCAGCGCCCAGCCTGCCCTCTGCCCCG

TCCTTTACTGTGTGGATGGGAACGCAAGTAAGAAGGAAAGGCTAAGCCTGGACTGCAGCT

GGCCTGCCAAGTCAGGAGGGAGCGTGGATCCTGGGATCGGTTTTTATAGAAATCAAGACA

GTTCTGTGGTTAAGCCTACAAATTAAAGGGAAATGGTTGCTGTGTATGTTATTGGTCCTT

GGAACTGCTGTCATTGAGGCTCATGAGGGACATGACGATGATGTGATTGATATAGAAGAT

GACCTTGATGATGTCATTGAAGAGGTAGAAGACTCAAAACCGAAACCAGAGACCGGCACT

CCTCCATCTCCAAAGGTCACCTACAAAGCTCCAGTTCCAACAGGGGAAGTGTATTTTGCC

GATTCCTTTGACAGAGGAACTCTGTCAGGGTGGATTTTATCCAAAGCCAAGAAAGATGAC

ACTGATGATGAAATTGCCAAATATGACGGAAAATGGGAGGTAGATGAAATGAAGGAAACA

AAGCTTCCAGGTGATAAAGGACTTGTGTTGATGTCTCGGGCCAAGCATCATGCCATCTCT

GCTAAACTGAACAAGCCCTTCCTGTTTGATACCAAGCCTCTCATTGTTCAGTATGAGGTT

AATTTCCAAAACGGAATAGAATGTGGTGGTGCTTATGTGAAACTGCTTTCTAAAACTGCA

GAACTCAACCTGGACCAGTTCCATGACAAGACCCCTTATACGATCATGTTTGGTCCAGAT

AAATGTGGAGAGGACTATAAATTGCACTTCATCTTCCGCCACAAAAACCCCAAAACGGGT

GAATATGAAGAAAAGCATGCTAAGAGACCAGATGCAGATCTGAAGACCTATTTTACTGAT

AAGAAAACACATCTTTATACATTAATCTTAAACCCAGATAATAGTTTTGAAATATTAGTG

GACCAGTCTCTTGTGAACAGTGGAAATCTACTAGATGACATGACTCCTCCTGTAAATCCT

TCACGTGAAATTGAGGACCCAGAAGACCGGAAGCCCGAGGATTGGGATGAAAGACCAAAA

ATACCAGATCCTGATGCTGTCAAACCAGATGACTGGGATGAAGACGCCCCTGCTAAGATT

CCCGATGAGGAAGCCACGAAGCCTGAAGGCTGGTTGGATGATGAGCCTGAATACGTAGCT

GATCCCGATGCGGAGAAGCCAGAAGATTGGGATGAAGACATGGATGGAGAATGGGAGGCT

CCTCAGATTGCCAACCCTAAGTGTGAATCAGCCCCCGGATGTGGTGTCTGGCAGCGACCT

ATGATTGACAACCCTAATTATAAGGGCAAATGGAAGCCACCCATGATTGACAATCCTAAC

TACCAGGGAATATGGAAACCCCGGAAAATACCAAATCCAGATTTCTTTGAAGATCTGGAA

CCTTTCAAAATGACTCCTTTTAGTGCCATTGGTTTGGAGCTGTGGTCCATGACCTCGGAC

ATTTTTTTTGACAATTTTATCATTTGTGGTGATCGAAGAGTCGTCGATGATTGGGCCAGT

GATGGATGGGGTCTGAAGAAGGCCGCTGATGGAGCTGCCGAGCCAGGTGTCGTGGGCCAG

ATGCTCGAGGCAGCTGAGGAGCGCCCATGGCTCTGGGTGGTCTATGTTTTGACTGTAGCA

CTGCCTGTGTTTCTTGTTATCCTCTTCTGCTGTTCTGGAAAGAAACAGTCCAGTCCTGTG

GAATACAAGAAGACTGATGCTCCTCAGCCAGATGTGAAGGAGGAAGAAGAGGAGAAAGAA

GAAGAAAAGGACAAAGCAGATGAGGAGGAGGAAGGTGAAGAGAAACTTGAAGAGAAGCAA

AAAAGTGATGCTGAAGAAGATGGTGGCACTGTCAGTCAAGAGGAGGGAGATAGCAAACCT

AAAGCGGAGGAGGATGAAATTTTGAACAGATCGCCAAGAAACAGAAAGCCACGAAGAGAG

TGAAACTATCTTAAGAACTTGATCTGTGATTTTCTCCCTCTCCCTCCCCTGCAAGTGTGG

TCCTAGGAGAGGACCGAGCACACCTTAGGTTGAAACTCAGAAAACCTCCAGATGTCGCCT

TCAACAGGTTTCAGTCAAACACTAGCCCGTGTAATTTTAAACATCTAGCAGTACATGCAA

TTGTGACATCAAGGACCCTGTTTCTGTAGAAAGAAAGCATTTAGCATAATGGTTGTGAAA

TGTAACATGAAGCAACTAACTTCTATTTTTTTTTTAAACATCTTTGTTTTTTAAAATAGA

GTGATAGAACTTTGCCAGTCTTTAAAAATCTTGGCTTAATTTAATATATTAATCTATCCC

TGCAGAAATAACACCAACCTTTAGAAATGCTAGGGGAATGAATACAGTTTTCGTAACCAA

TTTTGTTTAAGTTTGGTATTACAGAACGTTCAAGTGTCTCTGTCCCTTAAAATTGATAAT

CATGTTTAAAGTGCAGTCATTTGTGGTTATAATCTTGTGTCGCTTGCTTCCATGATTCAG

TTCCTCCTTAGGAAATTGAGAGGGATTGGATGGAAGCCCAAATTTATATAAAGTTTCTGT

TTAAATTGTATTAAAAATAGACATATAAAAAGAAAAAAACTTTTCAGTTGATGTTGGTTA

GACCATAAAGCGTGTGTGTTCTGTTGCCCTTGAAACAGCTCAGTTCCCAGATATCTTTGC

AGTTAGTGGAGGAGGTGGCATAATGTAGCACGTGAGCCTTCATACTCTACACACTGGGCG

CGTCTGGGCTCCAGCCTTTTTGAGCCAGTGCTTTTTTGAGGAACGCTACCAGGACATGTG

AGCTCCAGAGTAGATGCAGATGTTTCATCGCATCTTCCACTGTGTTGACACTGTTTTCTT

TTCCTTCCCCAAACTCCCAGCTTTCTCCTCTGCTATGCATTTTCTTCACAGCGCAGCTTG

CAGTCCGTTGCTGAAAATGATTATAAACTCTGCATAGTGTTAAGCTTTATTGTGATTACG

TGTATGTTTCTTCTTCCTTTTTTTAAAGCAGACCCACACCTTTCAAGGGTCAGAGTACAG

GATAGGATACAATCTTTCATTTTCATTGGTTTCTTTTACTCTGTGTAAAGACTTTAGAAG

TCTAAGCCAGAGGCGAGCCAATTCAAAATTGACTGTAATTGAACACAGGCTAAAAGTATT

GATGGGAGGAGTGACATAGAGCATGAGTTATCTGATTTTTGTAGCTGCTAAACCTTTTAC

GTTTTCATTTGCGGTTCACGTAATGTTGTGTCTTAACTTACATGATAAAGCAGTCCTGTT

CAAAAAATTTTTTTAAGTATGTGGCTTACAGAATTTTTCAAAAAGTGATGTTAGGTTTGT

TTTTTCACATGGAATGCAGATGGGTGCTATCAGAGCCTCTCCCCCATCACTGTAGTGTAA

TAATAATATTATTACATCACAATGAAGTGTATTCAGAATTAGGTGTTCTTTTAACTTTAT

TCTTTCTTCAGAGGTGTTTCAAATGTTACCGATGATACTGTTCCTTGCACTGAATATATA

AACACTCTAGAGTGTTTATATCGGGGAGATACTTGGGGAGGAAGTATATTTGTAAAAGAT

GAAGGCTGTATCTGTTTATTTTGTTTATCTTCAATTTTTTACTGGTTCACTTCAATTCTT

ATGAGGGTAGGATGCATTTTCTTGCTGTTCAGTACTTCATTTTCATCTGTTCTGTGGTCA

CAGTGACCTTAGCTATGTAGCAGACTTTCCCAAATGTATTGAGTGCAAATAAATAGTTAC

TTAGCAAGATCTGAAAATATGTCTGCAGGTTTATCCTTGAAGCAAATGTGTTTGGAATTG

TTGCATTTTTAGAAATCAGCCTTTTGCATTTTCCATTGATAATTCATTTTTCTCACAGTC

TCTTCCTGCTGAGCTGTGGAATGACAGGTGTAAATCGGAGTGTTCATTTGCTTTTCCAGG

GTTAGTTAGTAAAGTTTGTCTAAATCAAGTCTTTTCTGAGTTCTCTTTTTCACTCTTGTT

GAAGATGCAGCCTCCTGATTAAATGTCTGATACATTAATGAATGATCAGCAGCAGTTTTC

AACTCTTTAAAAGGCACTTACGTTGTGATTTGACATGCTAATTATCTGTCCCATGGTTGT

AAAATGCCCACTCCATCAGATGTTTGTCACTTTCTCTTGTCCACAAAATACTCTCCACTT

TTCTCAAGTTGTCGCATTGCTAAATGGTATTACATTAAAGCCCTGTGTTAAGCATCTGCT

TTTTGACTGAAC

>Locus31_286_Transcript_1/2_TAP1

CTGGTCTTGTTGGTCCTCTCCTGTCTTGGTGAGATGGCCATTCCATTCTTCACTGGCCGC

CTCACTGACTGGATTCTGCAAGAAGGGGCAGCAGCTGCCTTTACTCGAAACATAACTCTC

ATGTCCATTCTCACCATAGCCAGGTCTGGGGGCTGAAGATGGAAGGCTGAGGATGGGGGA

CTCTGAAAAAGAGGGAGTTGGAAGTTAGGGCTGCTGTCTAAAGTACACTTCTGTGGGGAC

CTTCTCTCTGTCTCCTGAACACACCCTGACTCTCCATCTCTCTGTCTCTAGTGCAGTGCT

GGAGTTCATGGGTGATGGGATCTATAACAGCACCATGGGCCGTGTGCACAGCCACTTGCA

GGGAGAGGTGTTCCGGGCTGTCCTGCGCCAGGAAACGGAGTTTTTCCAACAGAACCAAAC

AGGTGCCATCACATCTCGGGTAACAGAGGACACATCCACCTTGAGTGAGTCTCTGAGTGA

GAAGTTGAGCCTATTGCTGTGGTACCTGGTGCGAGGACTTTGTCTCTTGGGGCTTATGCT

CTGGGGGTCACTATCCCTCACCATGGTCACCCTGGTCTTCCTGCCTCTGCTTTTCCTTCT

GCCTAAGAAGCTTGGAAAATGGCACCAGGTACTGGCAGCACAGGTGCAAGAATCTCTGGC

AAAGTCCAACCAAGTGGCCATTGAGGTTCTGTCAGCTATGCCTACAGTCCGGAGCTTTGC

CAATGAGGATGGAGAGATCCAGAAGTTCAGGCAAAAGCTGCAGGAAATGGAGACACTCAA

CCAGAAGGAGGCCCTGGCCTATGCAGTCAACCTCTGGACCACCAGTATCTCAGGGATGCT

GCTGAAGGTGGGAATCCTATACATCGGTGGGCAGCTGGTGACAAGTGGGACTGTAAGCAG

TGGGAACCTTGTCACATTTGTTCTTTACCAGATCCAGTTCACCACAGCTGTTGAGGTCCT

GCTGTCCACCTATCCCAATGTACAGAAGGCTGTGGGCTCCTCAGAGAAAATATTTGAGTA

CTTGGACCGGACCCCTTGCTGCCCAGCCAGTGGTGTACTGACACCTTCAAATTTGGAGGG

CCTTGTTCAGTTCCAAGATGTCTCTTTTGCCTACCCAAACCGTCCAGATGTTCCAGTGCT

GCAGGAGTTGACATTCACCCTATGTCCTGGTGAGGTGATGGCACTGGTTGGACCCAATGG

GTCTGGGAAGAGCACAGTGGCTGCCCTACTGCAGAATCTGTACCAGCCTACCAGGGGGAA

GCTGCTATTGGATGGGAAGCCCCTTCCCGAATATGAACACCGCTACCTGCACAGACAGAT

GGCTGCAGTGGGACAAGAACCACAGCTATTTGGAAGAAGTTTCCAAGAAAATATTGCCTA

TGGCCTGATCCAGAAGCCAATTATGGAGGAAATCACAGCTGCTGCAATAGAGTCCGGAGC

CCATAGTTTCATCTCTGAACTCCCTCAAGGCTACAACACAGAGGTGGGCGAGGCTGGGGG

CCAGCTATCAGGGGGTCAGCGACAGGCAGTGGCCTTGGCTCGAGCATTGATCCGGAAACC

ACGTGTACTCATCCTGGATGATGCTACCAGTGCCCTGGATGCAAACAGCCAGTTACGGGT

GGAGCGGCTCCTGTATGAAAGCCCCGAGAGGTGCTCTCGGT

>Locus19_6654_Transcript_1/1_Cathepsin S

CTCTTCCGATCTAGTCAATGTAGCTGAAGTCACTTTGCTGGCTTTGAACTTTTAGAGAGG

ACCCACCTTCAAGCACTCCTAGTATGGGAGCACCTGCTGTTTCTAGCATAATGAAATGGC

TGGCTTGCGTGCTCCTGGGGTGCTCCGCAGCGGTGGCGCAGCTGCAGAGGGACCCCACGC

TGGATCGCCACTGGGACCTCTGGAAGAAGACTTACAGCAAGCACTACAGGGAAAAGATTG

AAGAAGTGGCACGGCGTCTCATCTGGGAAAAAAACCTAAAGTTTGTGATGCTTCACAACC

TGGAGCATTCAATGGGAATGCACTCATATGACCTCGGCATGAACCACCTGGGAGACATGA

CCAGTGAAGAAGTGATTTCTTTGATGGGTTCCGTGACAGTTCCCAGTCAATGGCAGAGAA

ATGTCACTTACAAGTCAAACCCTAATCAGAAATTGCCTGATTCTCTGGACTGGAGAGACA

AGGGGTGTGTTACTGAGGTGAAATACCAGGGTTCCTGTGGTGCTTGCTGGGCTTTCAGTG

CTGTAGGGGCCCTGGAAGCACAGCTGAAGCTGAAAACAGGGAATCTGGTGTCTCTGAGTG

CACAGAATCTGGTGGATTGCTCAACTGAAAAATACAGCAATAAAGGCTGCAATGGTGGAT

TCATGACAAGAGCTTTCCAATATATCATTGACAACAACGGCATCGATTCAGAAGCTTCCT

ATCCCTACAAAGCCCAGGATGGAAAGTGCCAGTATGACTCAAAATTTCGTGCTGCCACAT

GTTCAAAGTACACTGAACTTCCTTTTGGCAGTGAAGAGGCCTTGAAAGAAGCTGTGGCCA

ATAAAGGACCGGTGTCTGTTGCTATAGATGCGAGCCATCCTTCTTTCTTCCTCTACAGAA

GTGGTGTCTACTATGACCAGTCCTGTACTCTGAAAGTGAATCATGGTGTCTTAGTGGTTG

GCTATGGTAACCTTGATGGGAAAGACTACTGGCTTGTGAAAAACAGCTGGGGCCTGAACT

TTGGTGACAAAGGATATATTCGGATGGCAAGAAATAGTGGAAATCACTGTGGGATTGCCA

GTTACCCATCTTACCCAGAAATCTAGAGGATCTCTTCATTTTATAACAGGTTAAGAAAGA

TGAAACACTTTCTCTTCACTTAATTTTACCTGCTGTATCCAGTAGACATAAATGTGTCAT

GATCAATGTATATTTACTGTACTAACAGAATATATAGTTTGACTCTTCTACTTTTAACTT

TGCAGATCTTGGAACAAAGTTTACTAAGTAAAAATTAATAATTTACTGTAGATATAACTG

TATGAGAGTTGGTCAACCTAAGACAATCTGTTATGTTTATCATTGTCTTATTTTACACTC

TACGTCTTTTTAAGTCCCCTGATGTCCTTTTGTAACTTGACGGCATATAAATGTTTAATA

AATACCTGTCTTTTCAACCTTGTACCATTATTACATACCA

>Locus25_3030_Transcript_4/10_CD1a

TTTTGTTGCAGACAGTGACTAAAGGGGAGGTTTGTCTGTTGGTAGAAGAAGGAAGTCAAA

ATAGAGGTGTTGTGGGGTAGGTTTTTTTAAAACAGAAATCAAAGATAGACTTTTCTGAGA

GAAGAAATAACACCTGTAAATGAAATGCTGTTTCTGCAACTTCCATTACTAGCAGTTCTC

CTGCCAGGTGGTAACAATGAAGATGTCTTCCAGGAGCAGATCTTCCAAATCATCCACACC

TCATCTTTTTACAACCGTTCCTGGACACAAAGTTGGAGCTCGGGTTGGTTGGGAGACTTG

CAGACTCATGGCTGGGAGAGGAACTCGGGCAGAATCATTTTCCTGAGGCCTTGGTCAAAG

GGAAACTTCAGCAAGAAGGAGATGACAGAAATGGAAGGGCTTTTCCGCAGACTCTACATT

GAATTGTATCACATATTTCACAACCATGCCAGCCAATGGAAGTTTGAATATCCCTTTGTG

GTACAGATGGCAGCAGGCTGTGAGCTGCACTCTGGGGAAGCCAAGGAAGGCTTTAAGCGG

TATGCTTATCAAGGATCAGATCTCCTGAGCTTCCAGAATGATTCATGGTTGCCATCTCCA

AAGGGTGGAACTAGAGCTCAGCAGGTGTGCGGACTATTTAATCAGTACCCGGTTGTCAAT

GAAATAATACACAGGCACATCAGTGACACTTGTCCACGTTTTCTCTTGGGTCTACTTGAT

GCAGGGAAGGCAGATCTCCAGAGACAAGTAAGGCCAGAGGCTTGGCTGTCCATTGGCCCC

AATCCTGGTTCTGACCATCGGATGCTAATTTGTCATGTCTCTGGCTTCTACCCAAAGCCG

ATTTGGGCTATGTGGATGCGAGGTGAGCAGGTACAACAAGGCACTCAGCAAAGCGATGTC

TTGCCCAATGCTGATGGGACATGGTATCTCAGGATTTACTTGAAGGTGGAAACCATTGAC

ACATCTGGCCTGTCTTGCCGGGTGAGACACAGCAGTCTAGGAGGTAAGGACATCATCCTC

TACTTGGGTAAGAAGGAACTGGGGCCCAATTGGGAATGGGAGTAGGTGGTCCTCAAGCAG

AGCAGGAGAGGCAAATGAAAAATTGGGGATTTATGGACTCGAGACCAAAAGGAGTGAACA

TAATTAATTCAAGAAATAAAAGAGTTTAAAATTAGAGACCCCGCATATGTCGGAGAATTT

TGAGAGTTAGGTGAATGATTCATCCCTCAAGAGGAATGAGAGAAGAAATAGGAAGAAAGG

TGCTTGATGAAGTAGACACAGAAATGGAAAAAGTAATACCAGAGAAATGTACTAAACAGG

GGGTGGTTTTGAAATGATACCC

>Locus31_267_Transcript_5/11_CD1b

GAGTACTCTGGCAGCCCCCAGCATGGGTGTAGGGGGTCTGGATAAAAGCATGCTGCACTT

ACCCCTATGGTTCTGGGTCCTCCTGTATCCAGCCCTCCTCTCGTTCTGTAGGTCGGGGAT

GGCTTCTGAGGCCCTGCACAGGCAAATGTCTCAGGATTTATTTTGTTCTTCAGACTTTGG

GTTCTTTTGCTCTAGATCTTTTCTGTTTTCATTTTTTAAAAAATAATCCCTCCTGCTTCT

TTTCTCCTCACTTTTTTTTATCCTTCTACTACCCACAGCTTTCCAGGGACCAACTTCTTT

CCATGTCATTCAGATATCATCCTTTGCCAACAGCACCTGGGCACAAAATCAAGGCTCAGG

CTGGTTGGATGATTTGCAGATTCATGGATGGGATAGTGACTCAGGCACTGCTATTTTTCT

GAAGCCCTGGTCCAAGGGCAACTTCAGTGATGAGGAGGTGACTGAGCTGGTTGAGCTATT

TCGAGTCTATTTAATTGGATTCATCCGGGTAATACAGGACCACGTCAGTGAATTCCAGAT

GAAATACCCCTTTGAGATCCAAGGCATAGCAGGCTGTGAACTGCATTCTGGTGAGGCCAC

AGTAAGCTTCTTGAGGGGAGCTTTAGGAGGACTGGATTTCCTGAGCTTGAAGAATAATTC

ATGTGTGCCTGCCCCAGAGGGCGGCAGCAGGGCACAGCGATTCTGCACGCTCATCGTTCA

GTACCAAGGTTTCTGTGATATCGTAGAGAAGCTCCTCTTAGAAACCTGCCCTCGATATCT

CTTGGGTGTTCTCAATGCAGGGAAGGCGGAACTGCAGAGGCAAGTAAAGCCCGAAGCCTG

GCTTTCAAGTGGACCCAGTCCTGGGCCTGGCCGTCTGTTGCTGGTGTGTCATGTCTCAGG

ATTTTACCCAAAACCTGCATGGGTAATGTGGATGAGGGATGAGCGGGAGCAGCCAGGAAC

TCAGCAAGGTGACATCCTTCCCAATGCTGATGAGACATGGTATCTCCGAGTAACCCTGGA

TGTTGTGGCTGGGGAAGCAGCTGGTCTGAATTGCCGAGTAAAGCACAGCAGTCTAGGAGG

CCAAGACATTATCCTTTACTGGGGAAGCCCCACCTCCATCGGAGTGATATGTTCAGCAAT

AATAGTGCCTTCCTTGATTCTTTTGATATGTCTTGCATTATGGTTTTTGAGGCGCTGGTC

ATATCAGAATATCCCGTGAGCTTTCATCATTTCTCATTAATCATTTGGAATAAGTACTCA

GAAGACCAGAATCTCAAGTTTTTGGCCCAGGAGTCAATCTCATCATATTTAATCAAATAA

TCATCATATTTGATCAACTCAGAGTTCTCATAGGTTGTGAAATAAATCGTAATTTATACA

CCAGCAGAAAAATAATTAAGGACTGTGAATTTATTATGAGATTTTATCAATACTAGAATC

CACTCAGATTTTATAGATGTGAAATGTGAAGAAGAATGTATCTCAGAATAAATAAAATAA

AGTACATATGGTCTTACTTTTTTGCAACTTTAAAAAAGTAAAACCATCTTACAATTTTAC

AACTTTATAATGGCGTGAAAGTGATACACAATTCAGTAGAAATCATACTTTGAATTATGA

TTTTTTCTGGGCTAGTGATATGATGCTGGGCAGTGGCAGTGAACCACAGCTCCCACTTGA

TCAGGGGTGTCAACAACTGACACTCTATGATGTGTTCATTGTGTTAGATGATTTTGCTTA

ACTGCAGGCTAATGTAAGTGTTCTGAGCATATTTAAGGTATGCTAGGCTAAGCTGTGATA

TTCAGCAGGCTAGCCGAATTAAATGCATTTTTGACATTCAATATTTTCAATTTACAATGG

ATATATTGTTATGTAACCCTATCATAGGTCAAAGATCATCTGTATACAACTCAATGGTGA

CAGCACCTTGACTTACTATTTTAAAAAGTTTTTTTTTCTGCTTCTGCTGAAATATTATTT

GTCAAAGTAAGGTTACTATAATTATGCTGAAATAATTGTATTTGCAGAGATGCTGACCAT

TTTTTAATATAATTCTCTTTATCTGCCTGGATGACAACCCTCATGGCCCATCTGTGATGC

TTCTTTCTCTGAGAAGCTTTACCTAATGCTATAGTGAAAGTAACCCTCCCTTGGGTATTA

CACAAAGATCTCCTTTACTCTGTGCTCTCAAAATTGCAGTTATTAATGTTTTTATTTCTC

TTCTACTTGCAAGTCCTTTGACAGAAACCTTGCTCTAGGTGGATCCCTGGCAAGATCTTT

AACTTGAATATGTATCTGTTCAGTAAATATGATCTGAGTTAATATAACTGCTATGTTAAT

GGTCTTTTCTCAAAATCATTGTCTTCCTCTTTTTTTTCCTCTCTAGTTAATATTCTAGAT

TTCCCTTTTATTAAAATAGTTCTCACTTAGCAAAAAAAAAAAAAAAAAAAAAAAAAAAAA

A

>Locus27_926_Transcript_5/5_CD1d

CGGGAGCAGCCAGGAACTCAGCAAGGTGACATCCTTCCCAATGCTGATGAGACATGGTAT

CTCCGAGTAACCCTGGATGTTGTGGCTGGGGAAGCAGCTGGTCTGAATTGCCGAGTAAAG

CACAGCAGTCTAGGAGGCCAAGACATTATCCTTTACTGGGGTGAGAAAGAACTGGGGGCC

AGCTGTAAATGGGAGAGGATGATCCTCAGGCATTGAGGGAAGGACTAGAAAAATGATGAG

ACTTAATGGAGAGAGAGAGGGAGATGAGAGAGAGAGAGAGAGAGAGAGGA

**T cell receptors and co-receptors**

>Locus25_3929_Transcript_3/3_TCRA

ATATCACAGACCCCGATCCCGCCGTGTACCAGCTTAGAAACCCTAAATCCAACATTTCCG

TCTGCCTATTCACCGATTTTGATTCTGAAACCAATGTGTCACAAAAGACGGGGCCCACAG

TGTTCAGCACAGACAAAACCGCGCTGGACATGAGAGCCACAGGTTCCAAGAGCAACGGGG

CCCTGCTCTGGAGCAACAGCAATGATTTTGAATGCCAAGGCGCCTTCAACGAGACCTTCT

ACTCCAGCTCAGTCTTTCCCTGTGATGCCACATTGGTTGAGAAAAGCTTCGAAACAGACA

TGAACCTAAACTTCCAAAACCTGTCAGTGTTCGGGTACCGCATCCTCCTCCTGAAAGTGG

TCGGGTTCAACCTGCTCATGACGCTGCGGCTGTGGTCCAGCTGAGGTCACCAAGAATGCG

AGAGCCGAGTGCTCCCTGCCCCCGACTCCTCACCGCCCTCCTCTGCCTCTTTAAGCAGAG

AGGCACGCCCTCAGCCTCCATGAAGGAGAAGGCTCCCTCTGCAGCTCTGGCAATGCCACC

AACTGGATCCTACCAGATATTTGTGATTGAGATGCTGGAGAGCTACCAAGCGCTGCTGCC

ACCCCTCTGTTCCCTCACTGCTGCTTGTCACTGCCTAACGTTCCCGGCAAAGGCAGGGGC

TGCTGCAACCTCTCCTGGCTGTGGAGACGGCCCCTACCCCTCCCAGAGACTGCTTCTGAT

GTCCCACAGTGCGATGGATCCCCAGGGGCTTCTCCCAAGCTCTAGCTCCTGGAGAATGTT

GTGAGGAGCTTATATTTTTTAAATAGTGTTCATAAAGAAAGACATATTACCCTTTCTCCC

AAGATGTGGGGAAAACTCTCTCATTATCTAGACCCTGCTATGCTGTGTATCCGAGAGATC

GGAAGAGCGGT

>Locus31_1797_Transcript_1/7_TCRB

CGATGACCTGAGCAAAGTGAACCCGCCCAAGGTCACCGTGTTTGAACCATCCGAAGCAGA

GATCGCCCGGACACAGAAGGCCACTCTCGTGTGCCTGGCCACAGGCTTCTACCCCGACCA

CGTGGAGCTGAGCTGGTGGGTGAACGGCAAGAAGGTCCAGAGCGGGGTCAGCACGGACCC

TCAGGCCTACAAGGAGCAGCCCAGCCAGAGCGACTCCAGATACCGTCTGAGCAGCCGGCT

CAGGGTCTCCGCCACCTTCTGGCACAACCCCCGCAACCGCTTCCGCTGCCAAGTCGAGAT

CCACGGGATCAGTGACGAGGACGCCCGGAGTTCGGGAGACTCCGAACGCGTCACCCAGAA

CGTCAGTGCAGAAACCCGGGGCAAAGCAGATTGTGGCTTCACCGCGAAGTCCTACCAGCA

AGGGCTCCTGTCTGCCACCATCCTGTATGAGATCCTGCTGGGGAAGGCCACGCTGTACGC

GGTGCTGGTCAGCGCCCTGGTGCTGATGGCCATGGTAAGGAGGCCGGCAGGTTGGAGGGA

GAAGGTGGAACATCACATGTGGGATATGGGGGATCTCAGAGCCTGCCTCAGCTTATCCTT

TCATCTCGAGGAATTCGAAAGGGACCAGCAGCCTGGGGAGAAAGTGCAAAGATCATTACC

AAATGCAGCATCGGAGGATGGGGAGAATCAAACAGCTCTCCCCTAAATCAGGCCCGGAGT

CGGCCCCCTCTAACCTACTCCTGCTTGTGGCCACTGGGACCCTGACCATGTCCTTCTTCT

GCAGGTCAAGAGGAAGGATTCCTGAGACCGGCT

>Locus27_23431_Transcript_1/2_TCRG

AGAGAAGCAGCTGGTTGAGCAGTTAAGCCTAATCTCTTCTTTTCCTGCTCTTATTCTTTC

CATTTTCTATCAGTGCTTTTCAATGATTACTGCCTTGGTAGAAAATAGACATTTAGGTCA

ATTTCTCTGAGGCAAACTTATGAAACATCAGATGTCATGAAATGTATATTCGAGGACTTC

CAAAGGAACCCAGAAGATCAAATTGACAGCATATAGATTTTTCATTTCTGGTCATGGAGA

AGGGTGAGATTTTTGTAGGAGCTGTAACCAGTGTGATAGCTCAGGATGGATCAAGATATT

TGCCGAAGGAACTAAACTCATAGTAACTCCTCCTGATAGAAGGCTTAATTCAGACATCTC

TCCCAAGCCCACTATTTTTCTTCCTTCAATTGCTGAAATAAACCTACATAAGGCTGGAAC

GTATCTTTGTCTTCTTGAGAAATTTTTCCCTGATGTTATTAAGGTATCTTGGAAGGAAAA

GGACAGCAATACAATTCTGGAATCCCAGCAGGGAGATACCATCAAGATTAATGACACATA

CATGAAGTTCAGCTGGCTGACCGTGACTGCAAAGTCAATGAATAAAGACCACAAGTGTAT

CGTCAAACATGAGAATAATAAAAGAGGAGTTGATCAAGAGATTCTTTCTTCTTCAATAAA

GGAAGAGGTCGCTGCTATTAATAATGCAAAAGAAGCTTGTCTGAAAGACGAAAGTGATAT

GCTGCAGCTGCAGTTCACCAACACTTCTGCCTACTACACTTACCTCCTCCTCCTACTCAA

GAGCATGATCTACTTTGCCATCATCGCCTTCGGCCTGTTAAGGA

>Locus19_5150_Transcript_2/2_TCRD

GCCGGAGGCAAGGCACACACAATGTGCTTTAATAAATGTTAACAGAGATAGCGTCAGAAA

AGAATGGCAGAAAACCATCAACACTAACCCAGGTGAGAGTCAGTGTCCGGTGACCTCCAA

CTCCCTGCGTACAGATTGGTAGGCAAGAGATAGTAAAAATGCCGTTTTTACAGGAAACTC

TAGGGATCTCATTTCTATCCCTCTCTCCAAAAACACTTGGATGGTGTCTGGAACTTGGAG

TCAATTCATGTTTGTTAAATCAATTAATAGAAAGAAGTACATTTCCTCTGTATTTTACTC

TGATCTCTACTAATAGCTTCAAAGATATTTAGATAAGATTTTAGAAACCCCATTGCCTAC

ATGAAGAGTGTTGGAAAGTAGTGGGAGGGCAGAAAGTACTTTTGTGAATGAGACGCCAGT

TTAGGGTAAATCTAGTGCTGGGTAGAGCAGAGCCTTTCATGTTTAGGTAAAGGACGCCTA

CTCTTCTTCCCCCAGGGTCTGCTTTTCTCTTGGAGAATGGAGCTGGGGACCTGTTGGCTC

CCACTTTCTACTTCTGAAGGCAAAGAAAGTGAAAGGAACAAAACACCATTTTCAAAGCGT

ATTAGTTGTAGGAATTAAAAAACGAGCCCTCCATCCCTTTGTATCTTTATATCTATTTAT

AAAGCAGCATATAAAGCACTTAGGACCATAAACTCCTACCCCCCAGGACTTTTGTTTTTC

CATCAGGCTGGAGGATGAGCTGGGGTGGTGCCTTCAAAGGAAAACTGGTTTGGATAGCAG

TCATTTAAGCATATGAGAAAATAACTTGAATCACTGAGAGGTCCAAAGCCTAAGAGATAG

TATCTGGGTAGGCTTTTAGTTTCCTTCATGGAGTGTAGCTTTCTCATGCTGGTCAGCCTT

CAAAGAAAACTAACTTGACAGTCAAGAGAAAATTGATGGCAACACTCTTGGCAAATAGCA

TTCGCAGTCCTAGTACTGTGAGGGACATCATGTTCACCTTCCCGGGATGCACTTTGGGGT

CATGGCAGCTCTTTGAAGTCTGCTGTATGTCTTCAGGTTCCATTTGTTTTAGATCATCTG

AAGAAGTTGTATTCGGTTCAAAGTCAGTGGAGTACAAGATTTTATTGTTGTGTTGAACTG

AACATGTCACTGAATTAGAATCTTCATATTGACCAAGCTTGACAGCACTGTACTTCCCAC

TGGGAGAGACTACGATAGCGGGGTCAAATTCTACTATTCTCTTGGACGACTGGAGACTTA

TAGCTACATTCTTGGGGTAGAAATCCTTCACCAGACAAGCAACACTCGTCCCATTTTTCA

TGATGAAAACGGATGGTTCGGTAAGAGGCTGACTTTGGGGCTCCACGAAGAGTTCGGTTC

CAGCTCCAAAAATCATCTGCCGGGTGTCCCAGGAGCACATTGTGACACTGTTTCACAAGT

AACCAGCCTTGGGCTGGC

>Locus31mer_3631_Transcript_3/3_TCR zeta

TTCCTGCCTGTGAATCGAAAGGGGGTGTGAGGTGCTTGGCCCCGCGGGCTGGTGCCTGTC

TTCTCAAAGGCCCACGGTCTCCACTTCCTGAGGAGGCAGCACAGAACAAAGCCCGCAGAG

ACTCCCTGCCCTTCCTTGTCCCAGCCTCGGCGGCCACAGCCCCTGCCAGCCGGCGCTGTC

CTGGAGGAAGGGGCGAGATGAAGTGGACGGTGCTTGTGGCCACGGCCGTCCTGCAGGCTC

GGCTCCCAGCTGCAGATGCACAGAGCTTTGGCCTGCTGGACCCCAAACTCTGCTACCTGC

TGGATGGGATCCTCTTCATCTATGGCGTTATCATCACCGCCCTGTTTTTGAGAGTGAAGT

TCGGCCGCATCGCGGACGCCCCCGCGCACCTGCAGGGCCAGGGCGCGAACCAGCTCTATA

ACGAGCTCAACCTGGGGCGCAGAGAGGAGTACGACGTTTTGGATAAGAGATGGGGCCGAG

ACCCCGAGATGGGAGGGAAGCAGAGGAGGAAGGCCCCTCATGAAGGCGTGTACAACGCGC

TGCAGAAAGACAAGATGGCGGAGGCCTACAGCGAGATTGGCATGAAAGGCGAGAACCAGC

GCCGGAGAGGGAAGGGGCATGATGGCCTTTACCAGGGGCTCAGCACAGCCACCAAGGACA

CCTATGATGCCCTCCACATGCAGACGCTGCCCCCTCGCTAGCAGCTCAGGGATCCCACAC

TCACTGGCCAGCCCTGCAGGTGTCCAGATGGTTAGAGACCCAGGAAAAGCGTTTACGGCC

CAGCTCGCTCATGTTTCTCCACCACCAAAGTGGATGCTTCAAGCTGTGACATTTGGTCCC

GTCCAGTCCCAGACTGTCAAACACAGGGTGTTGTCCCCGGGCTATGGCCGGTCCTGGAGG

AGCCCACTGCAAGTGTGACACGTTCCGTCTTCTTGGTCGCTGGGGACCGTGGCTCTTACC

TGGAGGCGGGCACACCCCGCACAGCCCAGTCGTCAGTCCTGTGCTCTGCGACGTCCCCAG

GGGAACCCCAAATGTTAGCGGTCTGCGCTGCTCTGTCGGTGTTTGAGTGGCTTCGCTCCT

GCTGTAAATTTGGCGTCTGCTGTCGACTTTCTCCTCGTTTCTCGGTAGCTTGTCATTGGG

CCAAGGGGCTGTGCTTCCCGGGGCAGGTCGGGCGTGGGGGGGATGGGACAAGCCCCAGCG

GGTCCGGGTCCGCAGCGGCCAGAGCGCTTGGGAACCTGGGAAGGCCGTGCCAGAGTCCCG

GGCACGCGCCCTCCCCTGGGGACTGACAGATGAGCAGGCTGCCCCGCCCTCCGGAGAAGC

GAGCTGTGAACGAACAGTGTCACCGCAGCATGGACTGAGCGGGGTGGCAAGGCCACGTTC

ACCTCACAGTGACGGCAGGACAGACCCAACACAGTGAGGGCGTCGAGGGAGACGCCCCGG

CCGGCCGCTGGGAGCCCGCAGCGCAGCAAAGGGCTCGCGGCCCGGGTGGACACCTGTCCG

CCATGCTGGGGTTCGCTTTCTGCCACTCTGCTACAGCCTTCCCTGCACAATAAACGCCAG

ATCGGAAGAGCGGTTCAGCAG

>Locus25_2194_Transcript_5/6_CD3D antigen, delta polypeptide

CAACCCAAAAGACTATCAACAGATGAATCGATAAACAACTGTGGCATATCCATAAATGGA

ATACTACTGAACAACAAAAAAGGAATGAACTATTGAAAGATGCAACAAGATGGATGAATA

TCAAAATAATTATGCTGAATGTAAGAAGGCAGACCAGAAAAGGGTACATAATATATTATT

TCATGTATATTAACTATAGAAAAATGAGAACTAATCTGTAATGACAGAAAGTGGATCCGT

GGTCGCCTGGAGGGTGTGGAGATAAGGAGAGGGAGCGGGGAGGGATTGCATGCAAAGGGG

CACAAGGAAACTGTTCAAAGTGTTGGATACATTCATTTTCTTGATCGTGGTAGTGTTTCA

CAAGTGGGCTACATATGCCAAAGTTTGCCAAATTTTACGCTAAATATATGCTGTTTATTT

GTGTCAGTTTACCTCAGTAAATCTGTTTTTTAAAAGCACACAAAAAAATAGAGCCATAAG

GGACACAAGATTCTAGAAGGGCTCAGATGGAGGGAGGGCAGTTTTTCTCAACTTTGGCTT

CCTGTTAAAATCGACTTAAGTCCTGATGCTCCAGACCAATAAAATTAGAATTCCTGGGGG

TGGAATCACCCAGAAACCAGTATTTTTAAAAAGTTCCCAAGAGTTTCCAACATATGGCCA

ATGTTGAGAACTAGTGAGAGGTATGGTGGGATATAAAGGCATATGAGGAATCAGGTGAAA

GGAAGTGTGCAGTGTGTGAAAAGGATACAAGCACTGGTACAATACAAGCCATGAAGGGTT

GCTCAGTGGAGGTACTCAGTGTGAAAGCAAGATCTAGGGTTAGAAGGACTATGGTAGTAG

GGATATAAAGGGTAGGAATAAATCATAACCATGAATGCAGCATCTGTTGTAAATTGGAAG

TTCATTCTGTCTCTACCAAAATTGAGGTCTGTGTATATCGTGAAGAGATAGTGGTCAGAA

TCATCCAGATCTCTGGCAATATAGTTTTTAATTCTTTTTTCTTTGGGGAACCCTGGTCTA

GAAAATGCTTACTCTCGTTTAAATGCCCTGAAGTTCACTAAACTACTCTTACTCAGAGAA

TAAAAGGAGAGGTTCCCACCTATGGTAGACAGGAACAGGCATGAAGGACACAGAGTAAGG

GACCTCAGTTCTTCATTTAAACCAAGGACTCTGAATTGGGGTTGTTTCAGAATTACTCAG

GGACTACATGCCTAAAGTGGGGCCCACGTGTCTATATTCTTAACAATTCCCTCAGGCTCT

CAAAGATTCAGAACCACCACTAACCCACTAACCAAAGAAACCAAAGATGACATCCATTGA

AAATATCCTAAGTCCCCACCTAGTCTCAAACTCCAGCTTTCCAAGTGAGTGGAGAGGAAT

GCATTTTAATTGATGAAGGGTTTCTGTTGCCAGTTTATCCTGCAGGAGACAGGGGCAAAG

GTTCTGGGGAAAGTAAGATAAATGGTATTAAAGGGGTCTAGGCTAAGACTTCTCATAGCT

AGTGGCTACTGCACAATGAGGTTAAAAAGCCAAGGCCAGCCCCTTAGCAGAAGTGGGACT

TTCTCACACACATTCTCTGCATCTTGATAAGAAGTGAGTCTCAAGGCCTTTGATGTCTCT

ACCACCTCCGCTCGTGATGTCATGGAAACTTTTGTGCGGCTGAGTCCCTGAACACAGCAG

CCACCCTCAGCTGTGGTGAAGAAGCCAAAACCCAGGAAGGGTTACAATGGGGGGCTAACT

GGAGATAACAAATAGCGAGGGAGTACGTGGGGGCAGATCCTGAAGAGAGTGCACCAGGCC

ATAAACACCAGTGAGGCAGAAAGTGATGAGAAGCTGTCCATTAGACTGTTCATGTGGGGC

AGAGGACGGGAAGCAATGAAGGAATGATACAGATTGGCAGGGAGAGCAAGGACCCAAACA

AGGCAGGACAATTCCCATGCCTTGCTCAGGAGGCTTGAAAAGTCCAGGCGCCTGCTGAGT

GAAAGAAGACATATTTATTGACTGAACAAAGAGAGAAGGTGTGGTTGATAACTGATGCTC

CTATAAGCCACCAATCTCAGGTTCACTTGTTCCGAGGCCAGTTTTCACCAAGACGGCTGT

ACTGAGCATCATTCCGATCACGAAGGGGCTGATAGAGCTGGTCATTACTCAATAAATGTT

GAGTCTCAGCAGCCCTAGAGAGCCTTCCAGTCTCATGTCCAGCAAAGCAGTAGACTCCCA

AAGCAAGGAGGATAGTGGCAATGATGTCAGTGACAATGATGCCAGCCAGGGTAGCTGAGT

CCAGCTCCACACAGTTCTGGCACATTCTATAATATACTTGTAAAGTATATAATTTGATGT

CGTTTTCATATGTCTCATTGCACAGATACACTCCTCGTGGGTCCAGGATGCGTTTTCCCA

GGTCCAGACTTTGGTCGTTTGAGGACAGTTTTCCCCTTGTTCCCTGTAGCCATATGGTGC

TTCTATTGCAATTCAAAAGCACTTTGTCCTCAACTTCATCCACAAGATTCTGGGAGAGGC

TCACTTGGGAGAGAAGGGCAGCCAATATCAGGCCAGCCAGAAACCTGCTGTGTTCCATCT

TCCAACAGAATTCATTCAGTAGATAGACACAACCCACGCTGCAAGTAAATACAAAAGCCG

CCCACGGTGCATACAAAAAGGGCGGGTTATATAGTGTCAAAAGCTCCTGAAAACTCCTTC

ATCTGCCAGGCACTTAGGATGAGGAAAGGAGAGAAGGGTGGGGAGGAGGCAT

>Locus31mer_804_Transcript_3/9_CD3E antigen, epsilon polypeptide

GGTCGAGGGTAAACCAGCATTCCATCCCAGTCTTAGTTGCTCGCTTCGGAAAATGAAGTA

ATAAGCCAACTGGCCTCCACCATCTTAGGTAGAGTAAGTATCCAGTGAGAGAAGAATGCC

TTCGGGAAATCTCTGGAGAGTTCTGGGACTCTGCCTCTTATCAGTTGGTGCTTGGGGGCA

AGAAGATGAATCTTCTGTTATACTGAAAATATATAAAGTCTCCATCTCCGGGACAAAGGT

AATGCTGACATGCCCTGAGGATTCTGAATCTGGAACAATAATATGGGAAAAAGATGGTAA

ACCACGACGCACTAACGAAGACACGCTATTATTGGAGAATTTTTCAGAAATGGAGGATAA

TGGTTATTATTCCTGCTACACAAGTGCCTCTAAGAACAATCGCAATTTGCTCTACCTGAA

AGCAAGAGTATGTGAGAATTGCATGGAGGTGGATCTGATGGCAGTGGCCGTAATCATTAT

TGTTGACATTTGCATCACTCTTGGCTTGCTGTTGCTGGTGTATTACTGGAGCAAGAATAG

AAAGGCCAAGGCCAAACCTGTGACCAGAGGAGTGGGTACTGGTGGCAAGCCCAGGGGACA

AAACAAGGAGAGGCCACCACCTGTTCCCAATCCAGACTATGAGCCCATCCGGAAAGGCCA

GAGGGACCTGTATTCTGGCCTGAATCAGAGAGGCATCTGACAGCTCCCAAGGACACTGCT

TCCCATTGGCCCAGACCTGGCTCCTCTTCAGGCACCTGTTCCCTGGGCAAGTCTTGGACT

CCACAGAATTGTTCCTCTGCTTCTTGTGGAACTCACACCTGCAGCCTTGTCCCCAGCTCC

CTCCTTCCTGCCTTCTCTGCTAGTGCACAGTCCTGGGATATTGCTGCCTTATTGTCCTTT

GAAATATCATTGCTACTCACCCCTTCACACCTGGCCTGTACCTTTGTCTGAATATTTATT

TCTGCTGTTCATCACCGCCCCCAATTCCTGTCTTCCCTGCTCTCTCTTTGGCAACCCTCT

GTGGGAAT

>Locus31mer_3730_Transcript_3/3_CD3G antigen, gamma polypeptide

TTCAAGGTATTATTGCCCAATCAGAACAAGGAGAACTTTCAATAACAGTGGATGAGAATC

AAGAAGATGGTTCAGTACTTCTGATTTGTAAATCGAAAGATAAAAATATCAAATGGTATA

AAGATAGAAAGGAAATAATTTCACTATCTCAAAAAAAAAATAACGATACATGGAATCTGG

GAAGTCGTTTGAAGAACCCTCGAGGAACATATTGGTGTTCAACAGCAAACAACAGTTCAC

AACCACTCCAAGTGTATTATAGAATGTGTCAGAGTTGCATTGAGCTGAGTGCAGCCACTG

TGTCTGGCTTTGTCTTCGCTGAAATCATCAGCATTTTTCTTCTTGCTGTTGGGGTCTACC

TCATTGCTGGACAGGATGGAGTTCACCAATCAAGAGCTTCAGACAAGCAGACTCTGTTGT

CCAATGACCAGCTCTATCAGCCCCTCAAGGATCGAGAAGATGATCAATACAGCCACCTTC

AAGGAAACAAACTGAGGAAGAACTGAGCTCAGGACTCAGAGTAGGTGTTCTTTCTACTTA

GTTTTCAGAAACAAAGTGGTATATTTAGGAGCACTCCTAGCAGAGAAACTTTCAGGCCTA

AATCTAGACTCAAAGTTCCCAGAGGTGACAAATGGAGAAGAAAGTCCACCAGAGCAAATT

TGGGGTTTTCTCAAAATAAAATAAAAGTAAAAAATATGTATGGTGTTTCAGGAGTGCCAC

CTATTGGGAGGTAATAGTTTTGTAAAAGAAAAATGAAAAGATCAATAACTCCATTTGAAT

ATTTTTTTTTACATTTTACTTTTTATTTACTTTTTGCATGTCATAATTCACATGGGTCAA

ATATGCACTGAAAGATCTCCCTCCACCTACATTCCCAGTTGCCCTTTCCAGAGGCAAACA

ATATGCTGTTCTTTATATATCCTCATAAGACATATAATGTATCATATATCAGAGGTATTC

TAGGCATACATAAGCAAATTCTTAAGTATACATTTTTCTTCCTTCCCCTTTCTCACACAA

GTGGTATCATACTATATATCTTGTACTTTTCACCTAATGAAACTTGAACATGTTATTTTA

TACCAGTATATAAATTGGTTCCAATCTGTTGCTCTTAACAATGCTGCAATGACTAATATT

GTATAAATGTCATTTTTTAAAATA

>Locus31mer_1813_Transcript_3/4_CD4 antigen

AACCGCTCTTCCGATCTGCTCTCCCCAGTGGGCTCCTGGTGGCAGAGCTCCCAGCCCTCA

CTTAGGCACACCTGTGCAAGAAGCAGAAGCAGAGAGGACAAAAGACATAGGCTCAGAGGC

CCCGCCCTGTCTGCAGGGGAAGGCCCAGGATCTGCCTGCCTCAGCAAGGCCACAATGAAC

CTGGGATCCTCTTTCAGGCACTTGCTCCTGCTGCTGCAACTGGCGCTTCTCCCAGCCATC

ACTCAAGGAAAAGAAGTGGTGCTGGGTAAGGCAGGAGACAAAGCAGAGCTGCCCTGCCAG

GCTTCCCAGAAGAAGAGCATGAGCTTCAGCTGGAAATATTCTGGGATCATGGTTCTGAAC

TTCTTTCCGGCATCAACATTCTTGATGATAGGTTCCTCCTGGCTGAAAACACGTGTTGAA

TCAAAGAAAACCCTATGGGACCAAGGATCCTTTCCTCTGGTCATCAAGGATCTTGACATG

AAAGACTCAGGGATTTACATCTGTGAAGTGGAGGACAAGACAAAAGAGGTGGAATTGCTG

GTGTTCAGATTGAATGCTGATTTGGACATCCGCGGTGGCAGCATCCACCTGATGCCTGGG

GAGAGACTGACCCTGACCTTGGAGAGCCCGCCTGGTAGCAACCCTTCAATAGTATGGAAA

GGTCCAGGGAGTAAAAAGTATAATGGGGACAAGAGCCTCTCACTGTCCCAGCTAGGGTGG

CAGGAGAGTGGTACCTGGGAATGCATCGTCTCCTACAGCAAGAAGACACTGGTGCTCAGC

ATAAACATCTTGGTGCTGGCTATCCGGAAGGTCTCCAACACAGTCTATGCAAAAGAGGGG

GAGAAGGCGGAGCTCTCCTTCCCACTTACCTTTGAAGATGAAAACCTGGAAGGTGAGCTG

AGGTGGCAGCCGGAGGGGACTGCCTCCCTTCAGTCCTGGATCGCATTCTCCTTGGATAAC

AAGAAAGTATCTGTGAAGAAGGTTCACCCAAACTGCAAGCTCAAGATGAAGGAGTCGCTC

CCTCTCCTCTTCAGCCTGCTCCAGGCCTCGCCTCAGGATGCTGGCTCTGGAAACCTGACC

CTGTCTCTCAGAAAGGGGCAGTTGCATCAGGAAGTGAACCTTGTGGTGATGAGAATGACT

AAGTCCCAGAACCTTTTGACCTGTGAGGTGCTGGGACTCAGATCCCCCAAGCTGACACTG

AGCTTGAAGCTGGAGAACCAGACTGTGAAGGTCTCAGAACAGCAGAAGCAGCTAGTGGAG

ACGCCGGACCCTGAGGCAGGGACATGGCAGTGTCTATTGAGTGACGAGGACAAAGTCCTG

CTGGAATCCAAGACTGAAGTTCTGTCTGCAGGTTTCACTCAGGCCTGGCCAAAGCTCCTG

GTCATTGTGCTGGGGGGGATCCTGGGCTTTCTGATTTTCACCGGGATCTGCATCTTCTGC

TGTGTCAAGTGCCGGCACCGCAGGCGCCAGGCAGAGCGGATGTCTCAGATCAAAAGACTC

CTCAGTGAGAAGAAGACCTGCCAGTGCCCGCATTTGTCCAGGTGGCTGAGCCTCATCCCC

ACACGCCTTGAGCCTTCCAGAAGATCCCCGACTGGTCAAGAACCAGAGGAAGAAGAGACC

TGCAAGTCCTGAGATCGGAAGAGCGGTTCAGC

>Locus23_2175_Transcript_1/3_CD8A antigen, alpha polypeptide

TGTTCGCCCTCGCATCTTGAAATCTCTAATCTCTCTTTATCCTTCTGTCACTCCCACAAG

AGTGCCATTTTAAAGGACCATTTCCAAGCCTTTTCCCTGGTTCTCAAATCTGCAGTTTTC

AACTGCCCCCAGCCGCCGACAAGGTTGCCTGGATGGCTCCCATTCATGGGCCCCAGCTCC

CTCACTGGGCTGGAAGGTGGCAACCGCGAAAATGAGAACGACTCTATCCTCAGCGGGACT

TTAGATGACATCACATCCTCAGAACGCAAAATTGGACTCGGCAGCCGTCCGGAGGGCGCA

ACTTCCCGGCTCCGTATTTCGTTGTCTCCTGCGCGTTCTACTCGCGTCTGAGCTGAAAGC

TGAGCCGCGCCGCAGGGGTGCGCCATGGCCTTGCCGGTGACCTGGCTGCTCCTGCCCCTG

GCCCTGCTGCAAGCTGCCACGACCTTCGGGCAGCTGTCGTTCCGGATTTCGCAGAAAGGG

CAGAGTCACCTGGGCAAGCAGGTGGAGCTGCGCTGCGAAGTGCTGCTGTCCAGCCTGGCG

TCGGGCTGCTCGTGGCTCTTTCAGCCTCCTGGAGCCGCCACCAGCCCTGTCTTCCTAATG

TACATCTCCAAAATCCGGATCAAGACGGCCGAGGGGCTGAACAGCAAACAGATGTCGGGT

CAGAGGATCCAGGACACCGTCTTCACCCTCACCCTGCACAACTTCCGCGAAGAGGAGCAA

GGCTACTATTTCTGCTCGGTTGTAGGCAACTCGATACTGTACTTCAGCCCCTTCGAGCCC

GTCTTCCTGCCAGCGACACCCACCACGACGCCCGCGCCGAGACCACTCACGCGAGTCCCC

ACCAAAGCATCGCAGCCAGTGTCCCTGAGCCCGGAGGTGTGCCGGCCGGCAGCGGGCAGC

GCAGCGGACACGAGGGAGCTGCACTTCGCCTGTGTGATCTACATCTGGGCGCCTCTGGCT

GGGATCTGCGTGGTCCTTCTCCTATCTCTGGTCATCACGATCATCTGCAATCACAGGAAC

CGAAGACGTGTTTGCAAATGTCCCAGGCCTGTCGTCCGACCAGGAGGCAAGCCGAGCCCT

TCAGAGAGATATGTCTAACATGGCGATGGGCAGTTCAAGACTCCAACCTGAGAACTCCCC

TTACAAGGAGAGCAACGGTCTTCTTTTGGTTTTCATTCTCATTATTATTTTCGTGGGTGG

GGGTGGGAGGAAAGATTACTTTTTCTTTATGCCTTTACCTTGACACAAAGCAAAACTATA

TCATGTCTACAGTACACCGGAAGGATTACAATACCATTATGCACATGTATGGGCGGAAAG

GGCTGTGTTCTCGGAATCGGGCCGTTAGAGCTGGTGGGGGCCTCAAGGCCCCTTGGTGCA

AATCTTTTTCCCACCTATTTTACAAAGAAGAAGGCTGAAGTCCAGAGATTGGGAGAAGCT

TGACCAGAGTCACAGCAAGGCCAGAGCTGTTCCTCCGCACCGCTCAGGCCCTTCTTCCAG

GTGCCTCTGTCTCAGCACGGGGTTCTGTGTTTCAAAGCGCAAGGGAACAGGTCATTTCTT

GAGCACATGTGATAGACCCATTACTGCACACAGAACCCTGAGACGGTAATGAAATATGAA

AACTTCTGCCTTTCACAGAGTTCTATAATGTAGTCGAATGGTACCAGACTTTTTTTTTTT

ATTAATCAAGCATGAAATTGTATAGTTATTATATAAACAAGCATGAAATTCTTCAAATAA

AGTGGACGGGGTAAACTGAATCCTGGAAAATGTATAACTCGATCTCTAAAGGAAATCTCT

GAAATCAAGAAGTGGAGGCAAAATTGCTCTCCAAAACTTCATTGAAGAGATCGGAAGAG

>Locus25_14699_Transcript_1/1_CD8B antigen, beta polypeptide

GCTCTTCCGATCTCAGGTGTCCAGGCCAAGATGCAGCTGCGTCCCTGGCTCCTCCTCGCC

GCACAGCTGGCCGCTCTCCATGACAGCTCTGCACTCCAGCCAATTCTTGAGTACAAAATG

ACTGAGACCAACAGTAAGACGGAGCTGTACTGTGAAACCAAATACTCCTCCAGCAACACA

CGTATCTACTGGCTGAGACAGCGCCAGGCCCCAAGCACAAAGAGTCACCATGAGTTCCTG

GCCTTCTGGGATCCCACAAAAGGGGCTGTGTACGGTGAGGGTGTGAAACAGGAGAAGATA

ATTGTGCTTCAGGAGAAAAGCCAGTCCATTCTCAATCTCACAAGTGTGAAGATTTCCGAC

AGTGGTGTCTACTTCTGCATGACCATCGGGAACCCCGAGCTGACCTTCTGGAAGAGAATT

CAGCTGAGTGTGGTCGATGTCCTTCCCACCACTGCCCAGCCCACCAAGAAGTCCACCCCC

AAGAAGAAAAGGTGCCGGCTCCCAAGCACAGTGACCCAGAAGGGCCCACCTTGTGGCCCC

GTTATCCTTGGTCTGTTGCTGGCTGGTGTTCTGATTCTTCTGGTGTCCTTGGGTGTGGCT

ATCCACTTATATTGCCTGCGGAGGAAAGCCCGGCTTCGCCTCATGAAACAGTTTTATAAA

TGAGCAGAGAATACGCTTTTAGTGTCTTGCTACAAAGATAGTGTTGGTCAAGTAATGAGC

AAGATCTGGAACAAAACAGAAAAGGAACACACTGAATGGAAATGTCACTGACCTACAAGA

ACTGCCTGCTTTTAACTACTGCACATTCTTTCTGTGGGCCTCGTACATGGAAGCAACTTG

ATAAATGGTCGTCAGGAGACTTAGAGAGAACGCTTCAGAGCCCAGGGGCACTTCCTGCAA

CTCAGGGGCACACTGGAAGATTGAGCAAGAAATGCCACCCGTGATACCACTTCTAGCTCC

TGTATCCACCCTCGCTGGGCTGGGACCTTTGGTGTAGGCTCTCCAGCCACCGCCTAGCAA

GTCGCTTTGTTCTCAGCAGTGACATTGAGTTCTGAGTCATGGGATTGTGCTCGGATGACT

TCCCCTTGTTCTTCTCAGGCTGGGGTGGGCTGACTTCACAGAGAGCTTCGGAGACCAGAC

CCAGCCTGAAAATGAAGCTCGCAGGAAGGGCTGACCTAGCTCTCACAGTCCCAGAGGACT

TCCTCTGGGACTGAGAACTTGTGGCTTTGGGTGCAGGA

>Locus31mer_5135_Transcript_2/2_CD28 antigen

CTGGCCTCAATAGGTGCATCTTCAGCCTACCTCACACTTTGGGCTCCTCAGGGAGGAGGG

GCTGCAGCCCTAGCCCACCGTTGGCACAATGATCCTCAGGCTGCTCCTGGCTCTCAACTT

TTTCCCCTCAATTCAAGTAACAGAAAACAAGATTTTGGTGAAGCAGTCGCCCATGCTTGT

GGTGTACAACAATGCGGTCAACCTTAGCTGCAAGTATACCTACAACCTCTTCTCAAAGGA

GTTCCGAGCATCCCTTTATAAGGGAGCAGACAGTGCTGTGGAAGTCTGCGTTGTGAATGG

AAACTACTCCCATCGGCTTCGGTTTCAGTCAGCTCCAGGATTCAACTGTGATGGGAAATT

GGGCAACGAAACAGTGACATTTTACCTCCGGAATTTGTATGTTAACCAAACGGATATTTA

CTTCTGCAAAATAGAGGTCATGTATCCTCCTCCTTACATAGACAACGAGAAGAGCAATGG

AACCATTATCCACGTGAAAGAGAATCACCTTTGTCCAGCTCACCAGTTTCCTGACTCTTC

TAAGCCATTTTGGGCACTGGTGGTGGTTGGTGGAGTCTTGGCTTTCTACAGCTTACTAGT

AACAATGGCTCTTTGTATTTGCTGGATGAAGAGTAAGAGGAATCGGATCCTTCAGAGTGA

TTACATGAACATGACGCCCCGGCGGCCGGGACCCACCCGCAGGCTTTACCAGCCCTATGT

CCCAGCGCGTGACTTTGCAGCCTACCGCTCCTGACATGGACGCCTATCCAGATGCAAGCC

GGCTGGCACCTCTTCACCTGCTCAACACCACTGCTCTGGATAGGAAAGGACAGCCTCGTC

TTCAGCCAGCCATCTCAGGCTCCTATTAGGCCATCAATGCCGATTTTCCTATAATGACTG

GACCAAAGAACATCATTTTGAGACTCTGAAATGAAGTTAAAGAATCCTCCTGTGGCAGGC

TAAATTTTGTAGTGCCATGACCAATATTCAAACTTACTATGTATTTATTGACTTGATTGA

GAGGTTAGCTTTAAAATTTGCTTCCAGTTTTACTCCTTTCTCACTCATGTGCACATTGTG

GTCGAATAAAGTATGATATCGAAAGACATTTAATGGAGAAGAAAAGTTAGAAAATCATTC

CCTTTGGTTAAATGGGGGTTTAGAGTAGGAATAGATTAGAATGGGAGTAAGGAGACTTAA

ACATTTTTAAAACCATTATAACACTGTCTTTTACTCATGAAATGAGCCACTTCATTGATA

TTTAGTGCTGTTTTCCTTTTGGTTTAGAAAGATGTAGATATAGTCTTTGATGTATTCTGG

TCATATTCAGTTATTTTGACCGATGGAATACCTTCAAAACAATGCAAGGCAAACCAACTC

GCTTTCCTTACTCCCTGTGATGGGAGATCGGAAGAGCGGTTCAGGAGGAAT

**Immunoglobulin and B cell co-receptors**

>Locus29_86_Transcript_8/16_immunoglobulin heavy chain gamma (IgG)

CCGGAGTGTGGGAGCTGGATTTCTGGGTGTAGTGATTGTGCAGAGCCTCGTGCATCACCG

TACACGTGAAGTCCTGTCCTCGCTGCCACCTGCTCTTCTCCACGGTGAGCTTGCTGTACA

GGAAGAAGGACCCGTCGGCATCCAGCTGGGCCGGGGTCGTTTGGTACTTGGCCTCGGGCT

CTGGCTGCTCGTTGCTCTCCCACTCCACGTTGATGTCAGACGGGTAGAAGCCTTTGACCA

GGCAGGTCACGCTGACCACGTTCTTGGCCAGCTCATCTGGGTGTGGGTTCAGGACGTATA

CATGCGGCTCCCGGATCGGCCCTTTGGCCTTTGTGATGGTCCTCTCGATGGGGGCTGGGA

GGGCCTTGCTGTTGACCTTGCACTTGAACTGCTTGCCGTTCAGCCAGTCCTGGTGCAGGA

TCGGGAGGACGCTGACGATGCGGTAGGTGCTGTTGAACTGTTCCTCCGGCTCCTTCGTCT

TGCCCATGTTCGACTGCTTGCCGTCCACATACCAGGTGAACTTGACGTTGGTGTCATCCT

GGCTCACGTCCACCACTACACACGTGACCTCGGGCGTCCGGGTGATCATGAGGGTGTCCC

TGGCTTTCGGGGGGAAGATGAAGACCGAGGGTCCTCCCAGGAGCTCGGGAGCGGGACAGG

GGTTGCTGCCGGTGCCGCCGGTCCCGTCGATGCCGCCAGTGCCTCTGGTGCCTCCGGTGC

TGTCCGTGTCGCCGGTGCCGCCTCTGTTGCCTCTGCTGCCTCTGCTGTCGCTGCAAGTGT

ATTCCGCAGATACGATCTTGTCCACCTTGGTTTTGCTGGCCGGGTGGGCTACGTTGCAGG

TGTAGGTCTGGCCGGACGCGCTGCTTTTGGGCACTGTCACCATGCTGCTCTGAGAGTAGA

GCCCCGAGGAGTGCAGGGACGACGGGAAGGTGTACACGCCGCTGGTCAGGGCACCCGAGT

TCCAAGAGACGGTCACCGGCTCAGGGAAGAAGCCAGAGACCAGGCAGGCCAAGGAGATCG

TGGAGCCGGATGAAGCCTCTTTTGGCGGGGACAGAGGGAAGACCGACGGGGCCTTGGTGG

TGGCTGAGGAGACGGTGACCACGGTTCCCTGGCCCCAGAAATCAAAGTTATGAGTGCTAT

GTCTTGCACAGTAATATGTGGCCGTGTCCTCAGGCCGCAGACTGCTCAGTTCCATGTAGG

CTGTGCTGCTGGACTTCTCTGCGGTCATGGTGACTCTGCCCTGGAACTTCTGCGCATAGC

TTGTGCTACTATCTTCAGGATCAATTCTTCCCATCCACTCAAGCCCTTGACCTTGTGCCT

GTCGCACCCAGTGTATAAAATAGTTGCTGAAGGTGTATCCGGAAGCCTTGCAG

>Locus21_3346_Transcript_1/1_ immunoglobulin heavy chain mu (IgM)

CGTACACCTGCGTCGTGGGCCACGAGGCTCTGCCCCACTCGGTGACCGAGAGGACCGTGG

ACAAGTCCACCGAGGGGGAGCTGGGCGCCGAGGAGGAGGGCTTCGAGAACCTGAACGCCA

TGGCCTCGACCTTCATCGTGCTGTTCCTCCTCAGCCTGTTCTACAGCACCACAGTCACGC

TGTTCAAGGTGAAGTGACCACCCCCCAGAGCAGTGGAGAACAAGGAGACGCCATCAGGAG

GGGCCCACCGCCTGGAGCCGCCTGGCCTGCAGGCAGCGCCACCTGTGCTCAGATCTCCCC

GCACCAGCCCGCAGCCCCCTGGACACCGTCCGGCCTGGACACCGCCCGGCATTGCTTTGT

GACGTACCCGTGTGTCGCCTCAAATAAATGTGTATATTTTATCTTGTGAAACTGCTTC

>Locus19_10578_Transcript_2/2_immunoglobulin heavy chain epsilon (IgE)

ATCCACCCAGGCCCCGGCCGTCTTCCCCTTGGTCTCCTGCTGCGAAGGCACCAGCACCGC

CGCCCTCTCCGTGACACTGGGATGCCTGGTCAGGGACTACTTCCCAGGCCCAGTGACCGT

GACCTGGGATGTGGGGACCCTGACCAAGAACACTGTGACCTTCCCTGCCACCCTCCACTC

GACCTCCAACCTCTACACCACCATCAGCCAGGTGACCGCTTCGGGCGAGTGGGCCACACA

GAAGTTCACCTGCCAGGTGGAGCACCCCGGGTCCGCCGCCGTCAGCAGGAGCTTTCGTGT

GTGCGCCACGAACTACACCCAGCCCACCGTGAAGCTTTTACACTCGTCCTGCGACCCCAG

TGGCGACACCCATGCCACCGTCCAGCTCCTCTGCCTCATCTCTGACTTCACCCCAGGTGA

CATTGAGGTCACCTGGCTGGTGGACGGGCAGAAGGCCGAGAACGTGTTCCCATACATCAG

CCCCCACAAGCAGGAGGGCAAGCTAGCCTCCGCCCACAGCCAGCTTAACATCACCCAAGG

CCAGTGGGTGTCCCAAAGCACCTACACCTGCCGGGTCAACTACCTAGGCTTCATCATCGA

GGACCATGCCCGCAGCTGCCCAGAGTCCGAGCCCCGCGGCGTGAGCGTCTACCTGATCCC

GCCCAGCCCCCTCG

>Locus21_3437_Transcript_2/2_Immunoglobulin heavy chain alpha (IgA)

TGGTCACCGTCTCCTCAGAAGAGCAGACCGACCCCACGCTCTTCCCACTGAGCCTCAGCA

GCGTCGACGGCTCCGGGAACGCGATCATCGGCTGCCTGGTCTCCGGCTTCTTCCCATCGC

AGCCTGTGAGTGTGACCTGGAGCCACACCGGGCAGGGCACGTCCGTCACGAACTTCCCCG

CCGTGCAGACAACCTCCGGAGGCCTGTACTCCATGACCAGCCAGCTGACCCTGCCGGCCT

CCCAGTGCCCGGCTGAAGAGAAAAAGACATGCCAAGTCCAGCACCTGTCCAGCTCCAAGT

CTGTGGACGTGCCCTGCCAAGTCAGTAGCCCAACCCCATCACGGTGCCAGCCGAAGTTGT

CACTGCACCGGCCCGCGATCGAGGACCTGCTCCTGGGCTCCAATGCCAACCTCACGTGTA

CGCTGAGCGGCCTGGATGACGCCCAGGGGGCCACCTTCACCTGGGAGTCCTCGGGAGAGA

AGGAAGCCATCCAGGGCACCCCGCAGCGTGATGCCTCCGGCTGCTACAGCGTGTCCAGCG

TCCTGCCAGGCTGCGCCGAGCCATGGAACCGCGGAGAGAACTTCTCATGCACCGTCACCT

ACCCTGGGTCCACAAGCCCACTGACGGCCACCATCGCCAAAACCCTAGGAAACACCCTCC

GGCCCCAGGTCCACCTGCTGCCGCCGCCGTCGGAGGAGCTGGCCCTCAACGAGCTGGTGA

CGCTGACGTGCGTGGTGCGGGGCTTCAGCCCCGAGGAGGTGCTGCTGCGGTGGCTGCATG

GGAACCAGGAGCTTTCCCGGGAGAAGTACCTGGTCTGGGGACCCCTGCCAGAGGCCAGCC

AAAGTGCCACCACGTTCGCCGTGACCAGCATGCTGCGTGTGGAGGCTGAGTCATGGAAGA

GTGGGGACAGCTATTCCTGCATGGTGGGCCACGAGGCCCTGCCCATGGCCTTCACCCAGA

AGACCATCGACCGCCTGGCCGGTAAACCCACCCATGTCAACGTGTCCGTGGTCCTGTCCG

AGGTGGACGGCGTCTGCTACTGAGCTGCCTCCACCTGCCCACCCACCCTCCAATAAACTC

CATGCTCGCCCAGATCGGAAGAG

>Locus31_13381_Transcript_1/1_CD79A antigen

GGCCCAACGAGGTAGGATCTGGTACCCCACGACTGCTGCCAGCCAGACCCACCAACCCGC

TGGGAGAAGATGCCTGGTGGTGCCAGACTCCTTCAAGCTCGGCCTGCCATTGTCTTCTTC

GTCTTGCTTGAGGCCGCTGGCCTGTGTACTGGCCTGGGCACCAAGGACAAGACCCTGCCG

GTCCTTGACCACTGGGACCCGCCATCAATGGTCGTGATCTTGGGGGATGATGCCCGCCTC

CCATGCCTGCACAATAGCAGCAACCCCAGCAGCCATACCAATATCACATGGTGGCGCATT

CTCCACGGCAACTACACCCACACGTGGCCACCTCAGTACGAGGGCGAGGGCCAGGGCCCC

ACGGGTGAGATGACCATCCCGTCAGTGAACAAGAGCCACGTGGGCATGTACAGGTGCAAA

GTCCACTATGGTAACAGGAGCGTACAATCCTGTGGCACCTACCTCCTCGTGCGTGAGCCA

TACCCCAGACCCTTCCTGAACATGGGGGAGGGCACCAAGAACAACATCATCACAGCCGAA

GGGATTATCCTGCTGTTCTGTGCGGTGGTGCCCGGGACACTGCTGCTGTTCAGGAAACGA

TGGCAGAATGTGAAGTTTGGAATAGACGCCCAGGATGACTATGAAGATGAAAATCTTTAT

GAGGGCCTGAACCTCGATGACTGCTCCATGTACGAGGACATCTCCCGGGGCCTCCAGGGC

ACCTACCAGGACGTGGGGAGCCTCCGCATCGGAGATGTCCAGCTGGAGAAGCCGTGACCG

TGACCAGCTGGGCTGGCCCTGCCTGCTGTCCTCCCAACTCCCGTGTCTCTCCCGCTCCCT

AGAACCCGCTCTTTTCACAGTCTTTCCTGGGAGTGTCCCGCACTGCCTTCCCCCTTGGGA

GTGTCTGTTCTTCTTCCCTCTGCACTGTCCTCATGTCTGGCACACACCCTAACTCGGTCC

TCCCCCTGCTGCCTTTCCCTGGCTCCCACCCACCCAGCCAGCCGGTAATGAGCCCTTAAT

CGCTGCCTCTAGGGGAGCTGATTGTAGCAGCCTCGTTAGTGTCGCCTCCCCCCTCCCTGC

TCTGTCATGGCCACTTAGTGATAATAAATCCTTCCCC

>Locus25_36773_Transcript_1/1_CD79B antigen

GGGCTGGTCTCTCACTCCTGGCCTGGGTGCTCACCCACTGACCACTTCACCTCCCCTGTC

CGCAGAGTCACTATGTCCTCGTAGGTGGCCGTCTGGTCAATGTTCAGGCCCTCGTAGGTA

TGATCTTCCTCCATTCCAGCCTTGTGGTCATCCTTGTCCAGCAGCAGGAAGATGGGCACG

ATGATGAAGACGATGATGAGCAGGGTCTGGATCAGGATGATGCTATCTTTCAGTGTGTTT

CGCCGCTTCAGCTGCGCCAAGGTGCTGAACCCCATGACTCGAAGTTCGGTGCCACAGCCC

CTCTCAGGGGAGCCTTCTGGGCACTCTTGCTGACAGAAGTAGATGCCATTGTCCTGATAC

TGGATGTTACGGATGATGAGGGTGGCGTTGGTGGCGCTCTGGGTCTGCTCTATGTGTCCC

TGCTCCAGGTGCAGCAGCTTGGGCTCCGAGTCCGCAGCCTGCTTCCGGAGCCAGTTCACG

TTGCCCAGAATCTTCATGAAGCACTCTATTGTCACCGTGGAACCCCGTTTCTTGGCCATG

AAACGTGGGTGCTGCCAGATCTGGGAACAAGTGCTTCCTTCAGGATTCCTGTACAGGTCC

TTTATTTGGGCTTCCGGCACCATCTCACCTGCTGGAAGAAGCAGC

>Locus29_10528_Transcript_1/4_CD72 antigen

CTAAGGCTGTGCAGCCTGGTCCAAATACCTCCTTCTGAGCCTGCTCCTCACCTGCCTGCT

GTTAGGGATGGCTGCCATCTCCTTGGGAGTGCGCTATCTGCAGGTGTCTCAGCAGCTCCA

GCACACCAAAAGGGTTCTAGAAGCCACTAACAGCAGTCTGAGCCAGCAGCTCCGCATAAG

TAACAGCCAGCTGGGGCAGAGGGAACAGGATCTACAGGGGGCCAGGAGGGAGCTGGCCCA

GAGTCAGGAAGCACTACAGGTGGAACAGAGGGACTGCCAAGATGTCAAAGAGCAGTTACA

GGCCTGCCAGTCTGACAGGGAGAACACGAAGGAGACCTTGCGAAGTGAGGAGGAACGGAG

GAGGACCTTGGAGGAGAGGCTGAACCGCATGCAGGACACACTGAAGCCCTTGTTTATATG

CCCCTCACCAGATACCTGCTGTCCTGTGGGATGGATACTGAACGAGAGGAATTGCTTTCT

CATCTCACCTGTTAAAAGAAGTTGGAGAGAGAGCCAAAACTATTGTAAATCTCTGTCCTC

CAACCTGGCTGTAGTCAATGACTATTTTTCAGATTATCGCAGCAGCTTAAATAAGATGTT

AACTTCCAATGTTTTGTCTGATACATATTGGATTACCCTCAACTCTAATAAGGACCAGCA

GTGGACTAATGGTGTAAAAGACTCTGGGTCTTATGCTCAAAACCAAAGATGTATCAAGAT

ACAAGGCTGGTGGCCAAAAGTAAACCCAGCGGAGTGTAATAATCGTTTTCCGTGCATCTG

TGAGATGGCAGCTTTTAGGTTTCCAGATGGGGACCACTCTTTGCACTGAGCTGGGTAAGA

GTGAGGGATGGGACTGAGGCATGGGAACCTGAGGGGAGTGGGACTCAGCAGGGGCCCAGA

GGTCAGGGGGAAGCTTGTTCCTGGGAATGACAGTCATGCCCACAGCCTCCAGGCACTTGG

TCTGCTTATGTCAAGGAGGTTGCACAGGGAAGGCTGGGAGATGAAGGGAGGCCAGTGATG

GGGTTGTCTAGGGAGCTGGGGAGACAGAGTGAGCTGGATGAGGGTTTCTTAGTGACCATC

AACTTTGGGGTTCGCTGATGCTTTACTCTTTCAGATACTTATGCCAATAAGAGCCCATGC

CCCTCATCTCTAATCTGCAGCCTGCAGGTCCCAAGATCATCTCCCTCCAGCGTTTCCTCA

TTCTTCTTGAACAGTTCCCGGCCAAGGGCTAATCCATGCTCAACACTTCTAGCCAGTCTG

CTGCCCGCTTGAAATCCTATCCCAGAAACTGGACTGTTCCTGGGAAAAGGGGGAAGAAAC

CTCTAGAAGAGATTTTGGCGTCCCCAAGAACCTCCCATAGTGGAATGGGGGGTTGGGGGG

AGGAGGGCGCACGGGCTGAGTGGATAGGGGCAGCCCGGAGCCAGCCAGGCAGTTTTATTG

AAATATTTTTAAATAATCTGCATGTGTTAGTCTCGTGTGTCAGCAGTGCTGTGTCTGGTT

CAGTGATCCCCACGGGATAGCAAGCCCAGGGGTTGGATAGGAAATGTTCTGTTCCTTGAG

TCCATGCTGAGATTCCCCTATAGGGGCAGGATAGCCGGTCAGCCAATCTGGGGCTGGCAC

TGTCCAGCATGTGCTCTGTCCTCACAGGGCATCCTGAAGGCTAAAGTTGGAGGCCTGAGA

CCACAGGCTCCATTGCTAAGAGCGTGCCCCAGGCAGCTGCAGGCTAGTTGTGGGTGGCTT

GGCATGGTGCCCTCGGTGGCAGAGAAGACTGCCCGCCTCACAGTTCAGGTTGCGGTAGCG

GGCAACAGCTG

>Locus19_59024_Transcript_1/1_CD22 antigen

TGACTCCATCTCTCCAGAAGATGCTGGAAGTTACAACTGCTTGGTCAACAACTCCATAGG

ACAGAGCACATCTGAGACCTGGATGCTCCAGGTGCTATATGCACCTAGGAGGCTGTGGGT

GTCTATGAGTCCGAAAGACAGTGTGATGGAGGGGAAGAGGGCAGTCCTGACGTGTGAAAG

TGATGCCAACCCTCCCATCTCCCAGTACACCTGGTTTGACTGGAATAACCAAAAGCTCCA

CCA

>Locus25_18394_Transcript_1/1_CD19 antigen

AGAGGCCCCATCTCCTGGATCCACGTTCACCCCAAGAAGCCTAACATCTCATTGCTGAGC

CTAAACCTGAGTGAGGATGCCACAGTCAGAGAGATGTGGGTCCTGGGCACACTCAGAGGA

GGGGCTGTTCTGTGGCTGCACCGGGCCAAAGCTCAAGATGCTGGCACCTATCATTGTTAC

CATGGCAATACAACCATTAAGATGCAGCTGAAGGTCACTGCCCAGTCAGCAATAAGGCAT

TGGCTTCTGGAGACTGGTGGCTGGAGAGTCCCTGTTGCGACTTTAACTTATCTGATCTTC

TGCATGGGTTCCC

>Locus27_24872_Transcript_1/1_NLRC5

GAAGGTGGATCTCAGGTCCCTGTATCATGTGACCTTGCACTTCAGATCTAACGAGGAGCA

GAAAGTAGGGCGCTGTGGCAGGTTCACGGGCTGTGGCCTCAGCCAGGAGCACGTGGAGCC

ACTGTGCAAGTTGCTGAGGAAGTGTGAAGACCTCAGCCAGCTGGACCTCTCAGCCAACCA

GCTGGGTGATGATGGACTCAGGTGCCTCCTGCAATGTCTGCCTCGGGTGCCCATCTCTGG

ATCACTTGATCTGAGTGACAACGGCATCTCCCAGGAAAGCGCC

>Locus29_6360_Transcript_1/1_NLRP3

CCCATCTCTGCCAACCTGCGGGGCCTCTGCTCACTGGCCGCAGATGGGATCTGGAACCAG

AAGATCCTGTTTGAGGAGGGGGACCTGAGGAGCCATGGCCTGCAGAAGGCGGATGCATCC

GCATTCCTGAGGGTGAACCTGTTCCAGAAGGAGGTGGACTGCGAGAAGTTCTACAGCTTC

ATCCACATGACCTTCCAGGAGTTCTTCGCTGCCATGTACTACCTGCTGGAGGAGGAGCAG

CACG
